# Highly Acidic Electron-Rich Brønsted Acids Accelerate Asymmetric Pictet–Spengler Reactions by Virtue of Stabilizing Cation–π Interactions

**DOI:** 10.1021/jacs.4c09421

**Published:** 2024-10-03

**Authors:** Manuel J. Scharf, Nobuya Tsuji, Monika M. Lindner, Markus Leutzsch, Märt Lõkov, Elisabeth Parman, Ivo Leito, Benjamin List

**Affiliations:** † Max-Planck-Institut für Kohlenforschung, Mülheim an der Ruhr 45470, Germany; ‡ Institute for Chemical Reaction Design and Discovery, Hokkaido University, Sapporo 001-0021, Japan; § Institute of Chemistry, University of Tartu, Tartu 50411, Estonia

## Abstract

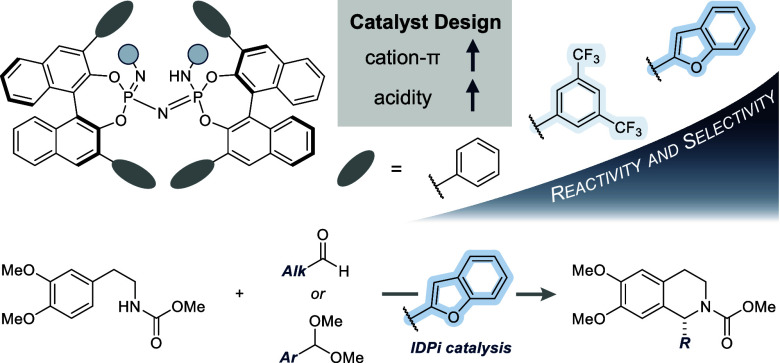

Electron-rich heteroaromatic imidodiphosphorimidates (IDPis) catalyze the asymmetric Pictet–Spengler reaction of *N*-carbamoyl-β-arylethylamines with high stereochemical precision. This particular class of catalysts furthermore provides a vital rate enhancement compared to related Brønsted acids. Here we present experimental studies on the underlying reaction kinetics that shed light on the specific origins of rate acceleration. Analysis of Hammett plots, kinetic isotope effects, reaction orders, Eyring plots, and isotopic scrambling experiments, allowed us to gather insights into the molecular interactions between the chiral Brønsted acid and catalytically formed intermediates. Based on rigorously determined p*K*_a_ values as well as the experimental evidence, we propose that attractive intermolecular forces offered by electron-rich π-surfaces of the chiral counteranion enthalpically stabilize cationic intermediates and transition states by way of cation–π interactions. This view is furthermore supported by in-depth density functional theory calculations. Our deepened understanding of the reaction mechanism allowed us to develop a method for accessing 1-aryltetrahydroisoquinolines from aromatic dimethyl acetals, a substrate class that was thus far inaccessible via catalytic asymmetric Pictet–Spengler reactions.

## 1 Introduction

The catalytic asymmetric Pictet–Spengler reaction is a powerful redox-neutral method for the construction of saturated six-membered *N*-heterocycles from β-arylethylamines and aldehydes.^1,2^ Due to the prevalence of the corresponding molecular frameworks as central structural elements in plant alkaloids,^3−5^ the reaction finds broad application in the field of natural product synthesis.^6−10^ Advances in method development over the past 20 years have continuously broadened the scope of accessible product classes. On the one hand, biochemical approaches for asymmetric Pictet–Spengler reactions usually leverage the promiscuity of the two most prominent natural Pictet–Spenglerases, strictosidine,^11^ and norcoclaurine synthase,^12−16^ respectively.^17^ In the field of chemical synthesis on the other hand, asymmetric organocatalysis has emerged as a particularly well-suited technology for accessing tetrahydro-β-carbolines (THBCs) from tryptamine precursors.^10,18−23^ Anion binding catalysts such as (thio)ureas or squaramide hydrogen bond donors (HBDs), as well as chiral phosphoric acids were successfully employed for this purpose within the conceptual blueprint of asymmetric counteranion-directed catalysis (ACDC).^24,25^ Accessing the product class of tetrahydroisoquinolines (THIQs) via this route, however, has proven a particularly challenging task, due to the reduced relative nucleophilicity of the reacting dopamine derivatives.^26−28^

**Figure 1:**
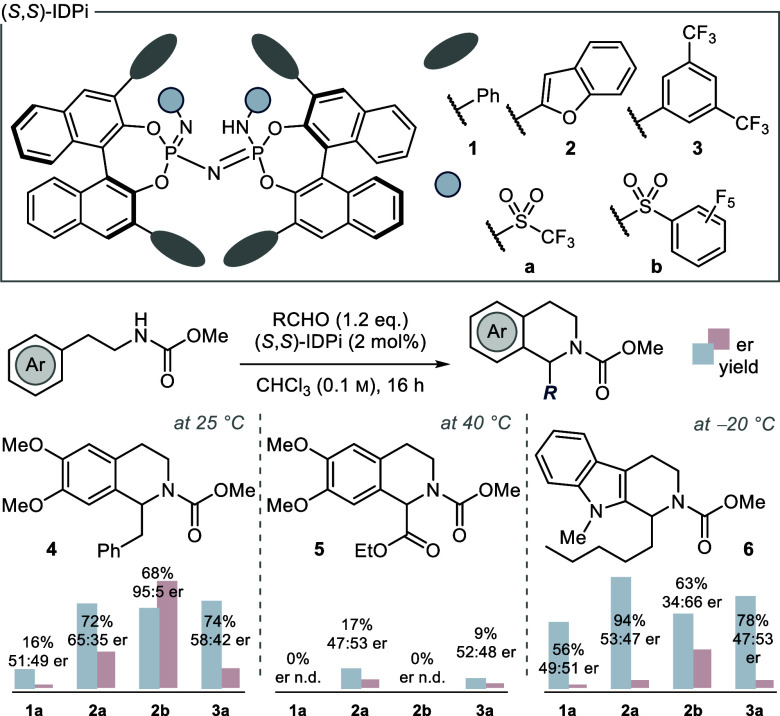
Key discovery of reactivity and selectivity enhancement by 2-benzofuran-substituted IDPi catalysts **2a** and **2b** across three different substrate classes. Yields were determined by ^1^H NMR spectroscopy of the crude reaction mixture using CHPh_3_ as internal standard.

We recently disclosed the Brønsted acid-catalyzed asymmetric Pictet–Spengler reaction of *N*-carbamoyl phenethylamines toward THIQ products and their biomimetic elaboration into divers natural product classes.^29^ The previously unprecedented transformation of poorly nucleophilic methyl carbamates that lack a free hydroxy substituent as activating and directing group was enabled by the design of electron-rich heterocyclic imidodiphosphorimidate (IDPi) catalysts. We discovered that the introduction of 2-benzofuranyl substituents in IDPis **2a** and **2b** enables a crucial rate- and selectivity-enhancement in comparison to electron neutral catalyst **1a** (Figure 1). The observed rate acceleration was found to be paralleled only by that of highly electron-poor catalysts such as **3a**—IDPis that are well-known for their high reactivity, purportedly by means of strong Brønsted acidity.^30−32^ We suspected the origin of the rate-enhancements with IDPis **2** and **3** to be of different nature, due to the electronic dissimilarity of the substituents. Consequently, we became interested in understanding the specific molecular interactions enabled by 2-benzofuranyl catalysts **2** fundamentally.

**Figure 2:**
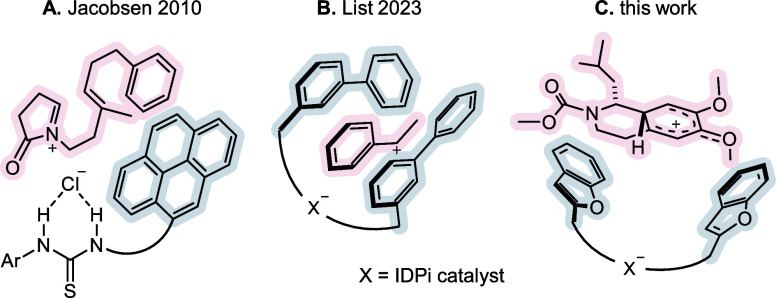
Selected examples of catalyst–substrate cation−π interactions in ACDC. (A) Polycyclizations.^37^ (B) S_N_1 reactions.^38^ (C) Pictet–Spengler reactions (this work).

The stereochemical outcome of asymmetric organic transformations has historically often been rationalized by invoking three-dimensional models, which focused on molecular interactions by means of destabilizing (“shielding”) the pathways toward the minor isomer. Recently, however, attractive noncovalent interactions (NCIs) are increasingly recognized as a fundamental principle in stereoselective processes.^33−35^ They have consequently emerged as a vital catalyst design strategy for the development of asymmetric methods.^36^ In the fields of Brønsted acid catalysis and ACDC in general, the purposeful introduction of π-donors for the stabilization of cationic transition states (TSs) has guided catalyst-development campaigns both in the pursuit of optimizing reactivity and selectivity. In 2010, Jacobsen presented the catalytic asymmetric polycyclization of cyclic *N*-acyliminium ions (Figure 2A).^37^ A positive correlation of reactivity and selectivity upon extension of the π-surfaces in the HBD catalyst was observed. The substituents allegedly serve as donors in cation−π interactions within the ion pair and thus guide stereoselectivity not by shielding, but rather by directing the cyclization through NCIs. Our group has recently contributed a range of catalytic asymmetric S_N_1 reactions, which demonstrate control over secondary benzylic carbocations.^38^ Computational studies illustrate stabilization of the cationic intermediate by NCIs with the chiral counteranion (Figure 2B). Even though specific π-complexes of benzofuran have been studied computationally,^39^ the 2-benzofuranyl unit has to the best of our knowledge previously not been recognized as a π-donor in asymmetric catalysis. We hypothesized that the strong catalytic Pictet–Spengler reactivity with IDPis of the type **2** originates from attractive NCIs between the 2-benzofuranyl substituents of the counteranion and the high-energy intermediates and TSs formed in the catalytic cycle (Figure 2C). Specifically, we suspected cation−π interactions to play a vital role in the reaction mechanism. By analysis of the relevant TSs through kinetic rate experiments and computations, we anticipated insights into the specific substrate–catalyst interactions within the ion pairs. We herein wish to present our findings.

## 2 Results and Discussion

**Figure 3:**
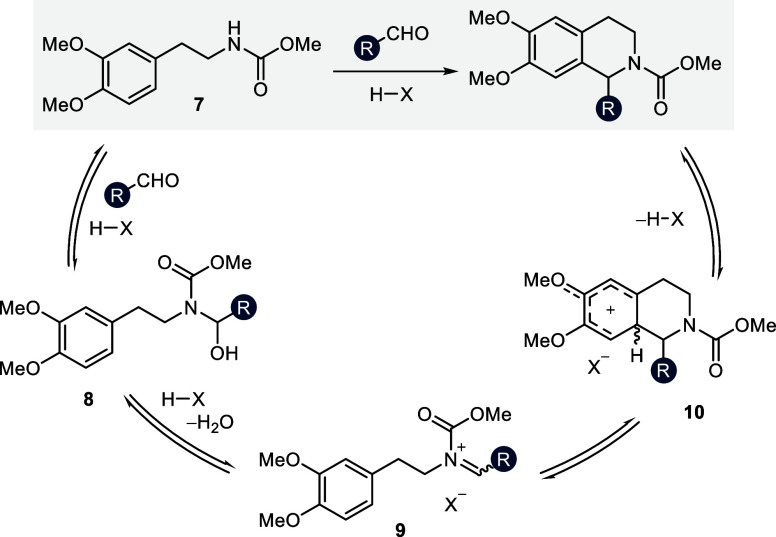
General mechanistic hypothesis for an acid-catalyzed Pictet–Spengler reaction.

The acid-catalyzed Pictet–Spengler reaction proceeds via a multistep mechanism, including several potentially slow pathways, which could theoretically be rate- and/or selectivity-determining (Figure 3). The reaction is initiated by the Brønsted acid-mediated nucleophilic attack of *N*-carbamoyl homoveratrylamine **7** onto the protonated aldehyde. The resulting hemiaminal **8** might be classified as a “fleeting chiral intermediate”.^40^ Subsequently, extrusion of water leads to the destruction of the stereogenic center and formation of the key *N*-acyliminium ion **9** paired with the catalyst counteranion. Nucleophilic attack of the aromatic ring furnishes arenium ion **10**, from which deprotonation by the counteranion leads to product formation and regeneration of the catalyst. Compound **10** can be formed as two diastereomers. While the relative configuration in **10** might be inconsequential to the absolute stereochemistry of the THIQ product, the relative stability of the four possible isomers in proximity to a chiral counteranion is decisive for the observed enantioselectivity, by means of their respective rates of formation and conversion. Similarly, the diastereospecificity of the nucleophilic cyclization with regard to the iminium ion geometry in **9**, as well as the stereoselectivity of iminium ion formation from hemiaminal **8** could theoretically lead to a translation of stereoinformation throughout the full catalytic cycle. All of these factors should be taken into consideration, when discussing the rate- and selectivity-determining factors in the catalytic asymmetric Pictet–Spengler reaction under study.

### 2.1 Acidity Measurements

**Figure 4:**
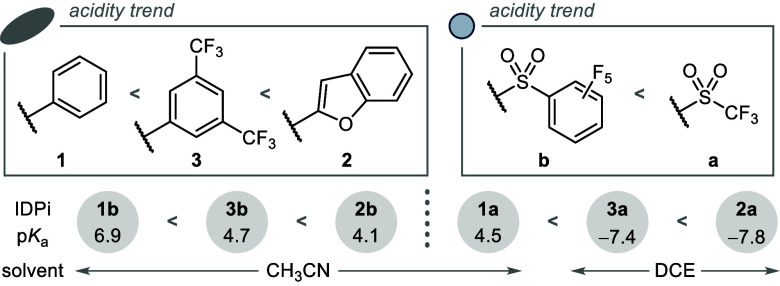
Experimental p*K*_a_ values and general structure-acidity relationships in IDPi catalysts.

The p*K*_a_ values of IDPi catalysts **1**–**3** were investigated by means of UV–vis spectrophotometric^41−45^ and NMR spectroscopic^46,47^ titration (Figure 4, see the Supporting Information for further details). Although we did not anticipate a strong acidifying effect upon installation of 2-benzofuranyl substituents in IDPi catalysts, we were pleasantly surprised to recognize catalyst **2b (**p*K*_a_ = 4.1 in CH_3_CN**)** as highly acidic in comparison to the parent phenyl-substituted IDPi **1b (**p*K*_a_ = 6.9 in CH_3_CN**)**. We attribute the observed acidification to the withdrawing inductive effects of the four benzofuran substituents on the BINOL backbone. Indeed, a similar acidity trend has been detected by comparison of benzofuran-2-carboxylic acid (p*K*_a_ = 2.79 in H_2_O) with benzoic acid (p*K*_a_ = 4.20 in H_2_O).^48^ We furthermore recognize the possibility for stabilizing effects of the 2-benzofuranyl units on the counteranion via an intramolecular hydrogen bonding network including polarized C–H bonds of the benzofuran substituents. The measured acidity of 2-benzofuran-substituted IDPi **2a** (p*K*_a_ = −7.8 in DCE) even surpasses that of electron-poor catalyst **3a** (p*K*_a_ = −7.4 in DCE). For these highly acidic catalysts, the basicity of the solvent prevented p*K*_a_ measurement in CH_3_CN. The titrations were therefore conducted in DCE instead.

The qualitative order of acidity was further substantiated by cotitration of catalysts **2b** vs **3b**, confirming the order of catalyst acidities based on their respective 3,3′-substituents as Ph < 3,5-(CF_3_)_2_–C_6_H_3_ < 2-benzofuranyl and thus establishing IDPi **2a** as one of the most acidic chiral Brønsted acid catalysts prepared in our laboratory. Nevertheless, the most selective benzofuran-substituted catalysts for catalytic asymmetric Pictet–Spengler reactions feature a perfluorinated aromatic core. The following discussion will therefore largely focus on IDPi **2b**, which shows an acidity profile comparable to that of parent IDPi catalyst **1a** (p*K*_a_ = 4.5 in CH_3_CN).^49^ Importantly, based on comparison of the p*K*_*a*_ values of catalysts with SO_2_CF_3_ (**a**) and SO_2_C_6_F_5_ (**b**) cores, IDPi **2b** can be estimated to be about two p*K*_a_ units less acidic than the electron-poor benchmark **3a**.

### 2.2 Hammett Plots

**Figure 5:**
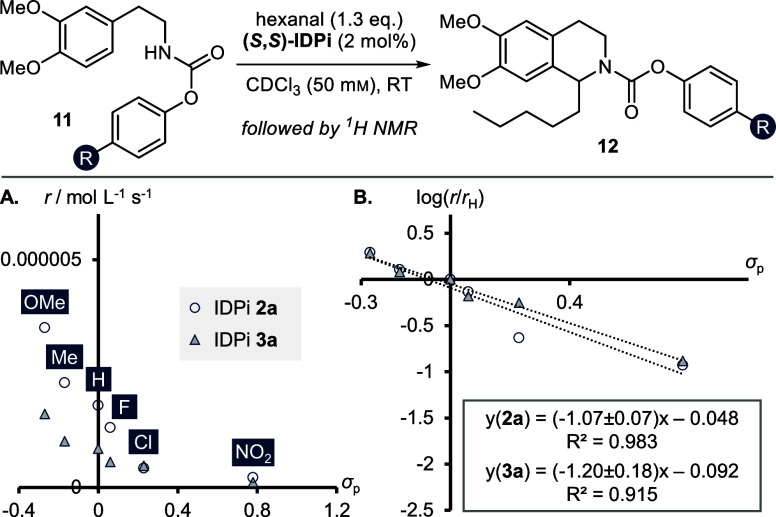
Hammett study with *para*-substituted aryl carbamates **11** and hexanal. (A) Early rates of individual experiments with IDPi catalysts **2a** and **3a**. (B) Hammett plots. Errors are given as the error of regression.

We synthesized *para*-substituted aryl carbamates **11** to examine the electronic influence of the protecting group on the overall reaction rate. We conducted individual rate experiments of both 2-benzofuranyl IDPi **2a** and 3,5-(CF_3_)_2_–C_6_H_3_ catalyst **3a**, allowing for direct comparison of the effect of the catalyst 3,3′-substituents, with six electronically divers carbamates **11** (Figure 5). Following product formation by ^1^H NMR spectroscopy allowed us to extract the reaction rates via initial rate approximations (≤30% yield). A Hammett plot of log(*r*/*r*_H_) against σ_p_ showed a good linear correlation for both catalytic systems **2a** and **2b** (with the *para*-chloro substrate being the only notable exception). We thus extracted large negative slopes of ρ(**3a**) = −1.07 ± 0.07 and ρ(**2a**) = −1.20 ± 0.18. These Hammett values are indicative for significant buildup of positive charge in a slow mechanistic step and hint toward either the nucleophilic attack of the carbamate onto the protonated aldehyde, or formation of *N*-acyliminium ion **9** contributing significantly to the overall reaction kinetics. Importantly, the critical cyclization step appears to be fast in comparison, as it would be accelerated by electron-poor carbamates.

### 2.3 KIEs

**Figure 6:**
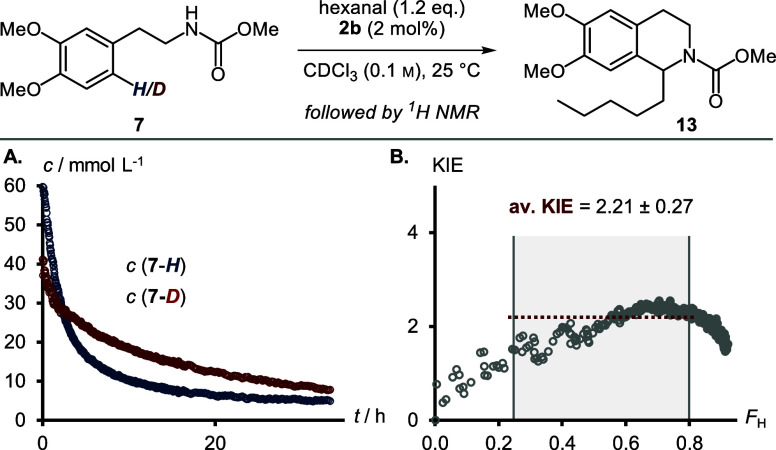
Competition KIE experiment using partly deuterated substrate **7** and hexanal (**14**). (A) Concentration profiles. (B) Calculated KIE according to eq 1. The average KIE was determined at fractional conversions of the protonated starting material 0.25 < *F*_H_ < 0.80. The error is given as the standard deviation.

The formation or breaking of C–C and C–H bonds in the rate-determining step (RDS) of a reaction can be probed by examination of Kinetic isotope effects (KIEs). We decided to measure the relative reaction rates of substrate **7** upon deuteration in the nucleophilic position of the aromatic ring by means of a competition KIE experiment (Figure 6). The deuterated and protonated starting materials were allowed to react with hexanal under the influence of IDPi catalyst **2b** in a single flask. Following the reaction progress by ^1^H NMR spectroscopy allowed us to determine the conversion of **7-H** and **7-D** at every single point of measurement. By examination of the respective concentration profiles (Figure 6a), it becomes qualitatively apparent that **7-H** is converted more facile than **7-D**. In order to quantify this effect, we utilized Singletons (eq 1), where *F*_H_ is the fractional conversion of the protonated substrate, *R* is the proportion of deuteration in the starting material, and *R*_0_ is the deuteration at the beginning of the reaction.^50^
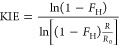
1

On the one hand, eq 1 is highly sensitive to small deviations in the concentrations at low conversions. On the other hand, as the reaction approaches full conversion of the protonated starting material, the accuracy of the calculated KIE is reduced by error of the NMR measurement at low remaining concentrations. We therefore averaged the calculated values at fractional conversions 0.25 ≤ *F*_H_ ≤ 0.80 and obtained a KIE of 2.21 ± 0.27 (Figure 6b). This value constitutes a pronounced primary KIE, which reveals a slow deprotonation of the arenium ion intermediate in the catalytic cycle. Indeed, a significant primary KIE has also been measured in comparable catalytic asymmetric Pictet–Spengler reactions: The conversion of unprotected tryptamines into THBCs catalyzed by Brønsted acids and chiral HBDs reportedly shows a primary KIE of 4.4 in an analogous competition reaction.^21^ This high value is consistent with the high nucleophilicity of a primary amine and an indole fragment, which accelerates the mechanistic steps prior to the final deprotonation and thus provides a more pronounced contribution to the overall rate. The biocatalytic reaction of dopamine and 4-hydroxyphenylacetaldehyde under the influence of NCS proceeds with a smaller primary KIE of 1.7.^51^ Computational studies have nurtured the view that the final deprotonation step is not only rate-, but also selectivity-determining in both catalytic systems.^12^

In light of the observed pronounced KIE, our measured Hammett values of ρ < –1 must be interpreted in terms of more subtle effects on the mechanism prior to the final deprotonation. The rate of *N*-acyliminium ion formation is positively influenced by electron-donating carbamates. As the subsequent nucleophilic cyclization is fast in comparison, this translates into a higher concentration of the arenium ion, the deprotonation of which determines the overall rate of the reaction. Furthermore, in line with the concept of microscopic reversibility, we recognize the possibility for the formation of all possible stereoisomers of hemiaminal **8**, iminium ion **9**, and arenium ion **10** in a dynamic equilibrium. The irreversibility of the final deprotonation step would establish a Curtin–Hammett scenario, where the enantioselectivity of the overall reaction is determined only by the relative rates of deprotonation within said equilibrium of intermediates.

### 2.4 Determination of the Reaction Order

**Figure 7:**
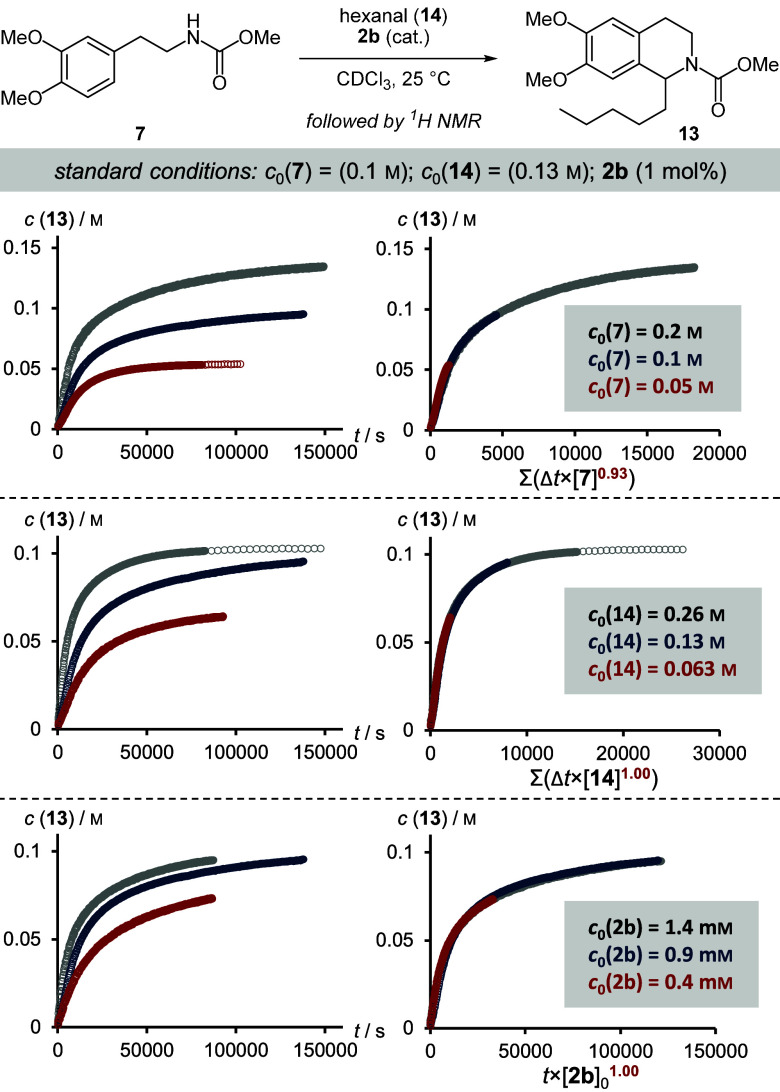
VTNA of the catalytic asymmetric Pictet–Spengler reaction of carbamate **7**, hexanal (**14**), and IDPi catalyst **2b**. Product concentrations were followed over time in separate experiments with variable initial concentrations of substrates and catalyst. Normalization of the time axis was visually optimized by varying the respective order.

The order of the reaction with respect to the starting materials and the catalyst was determined by variable time normalization analysis (VTNA).^52−54^ For a catalytic intermolecular reaction, the rate law can be summarized as d[P]/d*t* = *k*[A]^α^[B]^β^[cat]^γ^. To elucidate the order in each component of the reaction, the time scales were normalized analogously to ∑[A]^α^ × Δ*t*. The respective exponents were varied until an optimal visual overlay of the reaction profiles was achieved (Figure 7). We utilized different initial concentrations of carbamate **7**, hexanal (**14**), and IDPi catalyst 2**b**, and found the reaction to be first order in both substrates and catalyst. However, a slight deviation from 1 was measured for carbamate **7**. This effect might be rationalized by invoking competitive binding of either starting materials or product to the Brønsted acid catalyst. Nevertheless, we assumed an overall second order behavior for the following discussion.

### 2.5 Variable Temperature Studies

**Figure 8:**
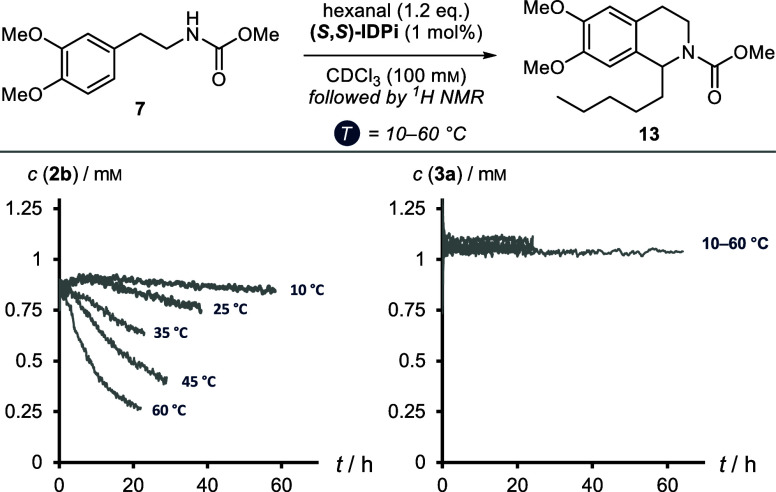
Hydrolytic stability of IDPis **2b** and **3a** under the reaction conditions at different temperatures.

When we followed the reaction profile by ^1^H NMR spectroscopy, we noticed partial decomposition of IDPi catalyst **2b**. In fact, the deactivation pathway could be confirmed to be hydrolysis of the iminophosphate functionalities to the corresponding iminoimidodiphosphate and imidodiphosphate structures. When we examined the reaction profile at different temperatures, we saw significantly accelerated decomposition of IDPi **2b** at elevated temperature (Figure 8). Catalyst **3a** on the other hand was found to be stable toward hydrolysis at all temperatures.

In order to obtain the overall kinetic rate constants for the reactions at different temperatures, accurate quantification of the active catalyst concentration is imperative. For a catalytic second order reaction, the integrated rate law (eq 2) allows for graphical linearization and extraction of the kinetic rate constant *k*.
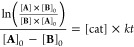
2

**Figure 9:**
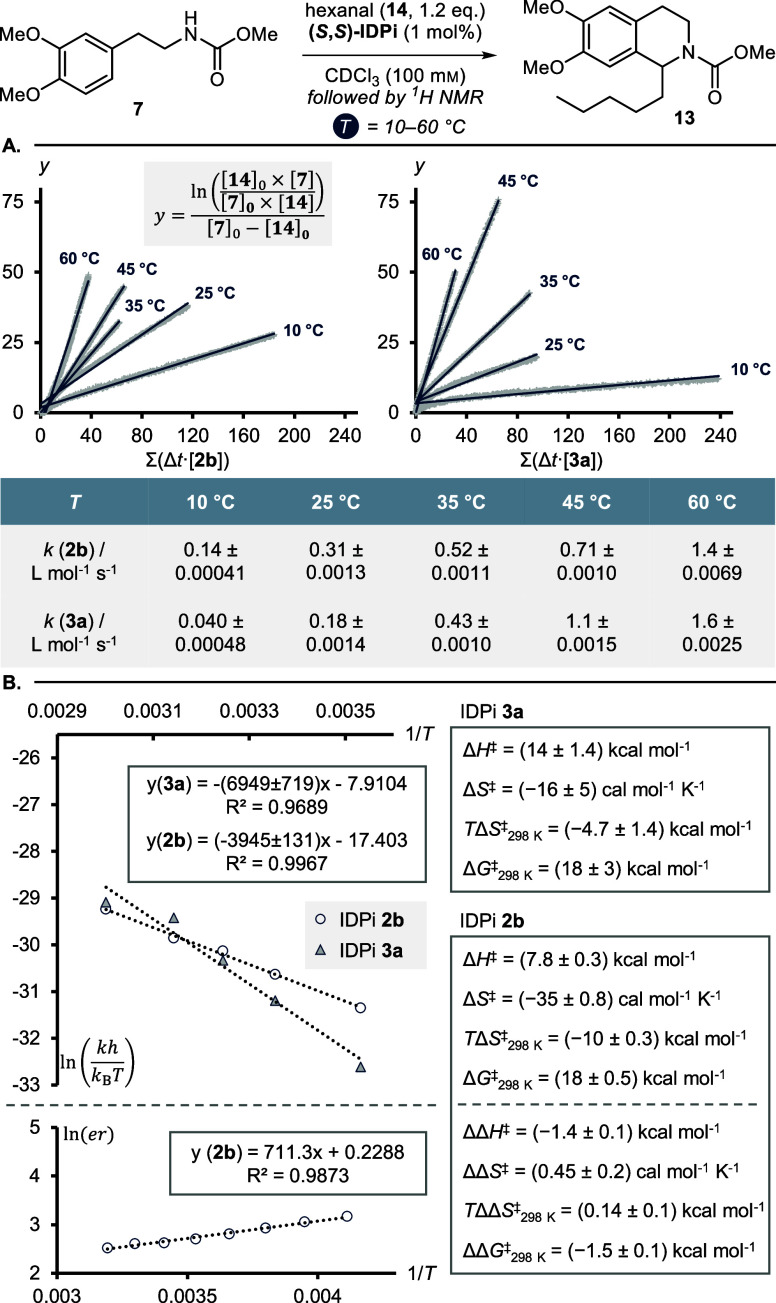
Variable temperature studies. (A) Second order rate constants. (B) Eyring plots and thermodynamic data for the absolute activation barriers (top) and the relative diastereomeric reaction barriers (bottom). Errors are given as the error of regression.

However, as the active catalyst concentration of IDPi **2b** is changing over time (see Figure 8), the *x*-axis has to be manually adjusted to display the integrated catalyst concentration until each point in time. Fortunately, integration of the catalyst signals in the ^1^H NMR spectra was sufficiently accurate, and normalization of the axis to ∑(Δ*t*[cat]) delivered linear profiles at each temperature for both catalysts **2b** and **3a** (Figure 9A). We were thus able to obtain the kinetic rate constants *k*. While catalyst **3a** is slightly more reactive at temperatures above 35 °C, 2-benzofuranyl IDPi **2b** facilitates faster product formation at low temperatures. From the qualitatively observable difference in temperature dependency, we extracted the respective thermodynamic activation parameters. We utilized the kinetic rate constants *k* in an Eyring plot according to eq 3.
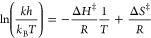
3

Again, we saw a good linear fit for both catalysts **2b** and **3a** (Figure 9B). Importantly, the dissimilar temperature dependency is reflected in vastly different thermodynamic contributions to the free enthalpy of activation. IDPi **3a** shows a relatively large enthalpy of activation (Δ*H*^‡^ = 14 ± 1.4 kcal mol^–1^). In contrast, 2-benzofuranyl IDPi **2b** facilitates the reaction through a drastically reduced enthalpic barrier (Δ*H*^‡^ = 7.8 ± 0.3 kcal mol^–1^), which is however counterbalanced by an increased entropy of activation (*T*Δ*S*^‡^_298 K_ = −10 ± 0.3 kcal mol^–1^). In a similar approach, we measured the enantioselectivity with catalyst **2b** at different temperatures. We were thus able to obtain the difference in thermodynamic contributions in the diastereomeric TSs leading to the two possible enantiomers. Fascinatingly, the stereoselectivity with 2-benzofuranyl IDPi **2b** seems to be controlled solely by the difference in activation enthalpy (ΔΔ*H*^‡^ = −1.4 ± 0.1 kcal mol^–1^) rather than entropy (*T*ΔΔ*S*^‡^_298 K_ = 0.14 ± 0.1 kcal mol^–1^).

By comparison of the thermodynamic data for both catalysts, it becomes apparent that both high reactivity and selectivity in the catalytic asymmetric Pictet–Spengler reaction with 2-benzofuranyl IDPi **2b** are enabled through enthalpic stabilization of the relevant TSs. This observation can be interpreted by invoking the possible effects of NCIs on the reaction barriers. Strong attractive cation–π interactions lead to more ordered TSs and ion pairs, due to energetically favored conformations. This however comes with an entropic penalty, due to overall reduced degrees of rotational and vibrational freedom. Catalyst **3a** offers low-energy reaction pathways by means of stabilizing solely the counteranion via electron delocalization and the inductive effect of the fluorinated 3,3′-substituents. The electrostatic interactions within the ion pair therefore only play a minor role. Consequently, the reaction proceeds through relatively dissociated ion pair intermediates, which are entropically favored. The measured dominant enthalpic stabilization in both the absolute and relative free enthalpies of activation with 2-benzofuranyl IDPi **2b** are thus in good agreement with the hypothesized stabilizing cation–π interactions from the 3,3′-substituents onto the cationic reaction intermediates and TSs.

### 2.6 On the Relevance of an Off-Cycle Enecarbamate

**Figure 10:**
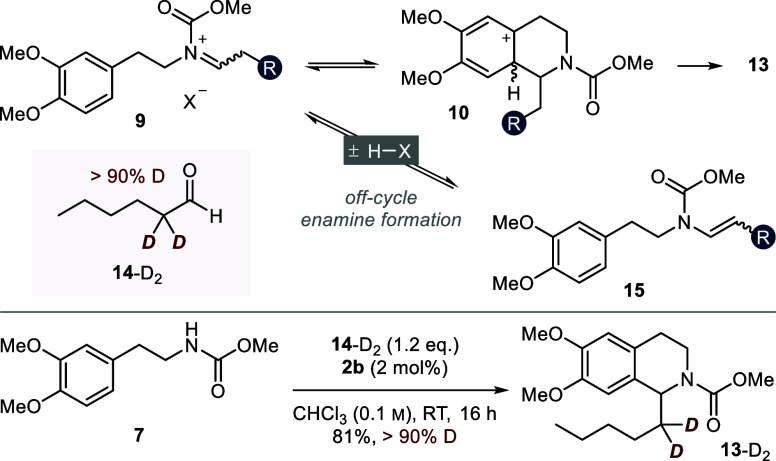
Theoretical pathway toward an off-cycle enecarbamate intermediate **15** and an isotope scrambling experiment using α-deuterated hexanal (**14**-D_2_).

As the developed catalytic asymmetric Pictet–Spengler reaction involves aliphatic aldehydes, we recognized the possibility for the formation of enecarbamate **15** by deprotonation of the reactive *N*-acyliminium ion. Similar compounds have been isolated by Hiemstra and co-workers, while studying a catalytic system using (*S*)-TRIP as organocatalyst.^27^ Presumably, the relatively strong basicity of the counteranion facilitated deprotonation of the iminium ion, which led to accumulation of an isolable enamine. We could however not observe the buildup of any side products or intermediates in significant amounts by ^1^H NMR spectroscopy, which does however not conclusively exclude the formation of a reactive enamine intermediate under catalytic conditions. We therefore decided to probe the formation of **15** experimentally through a deuterium scrambling experiment (Figure 10). If enamine **15** was formed under the reaction conditions, dissociation of the neutral compound from the Brønsted acid catalyst could lead to proton incorporation upon reprotonation toward iminium ion **9**. The H_2_O, which is necessarily formed under the reaction conditions, would serve as the proton source in this scenario. When we subjected carbamate **7** to a reaction with α-deuterated hexanal (**14**-D_2_), we could isolate THIQ **13**-D_2_ with >90% deuterium incorporation, as determined by ^1^H NMR spectroscopy and mass spectrometry. We thus conclude that the formation of enamine **15** is unlikely under optimal reaction conditions. Nevertheless, we cannot exclude it in the case of more α-acidic phenylacetaldehydes.

### 2.7 DFT Studies

**Figure 11:**
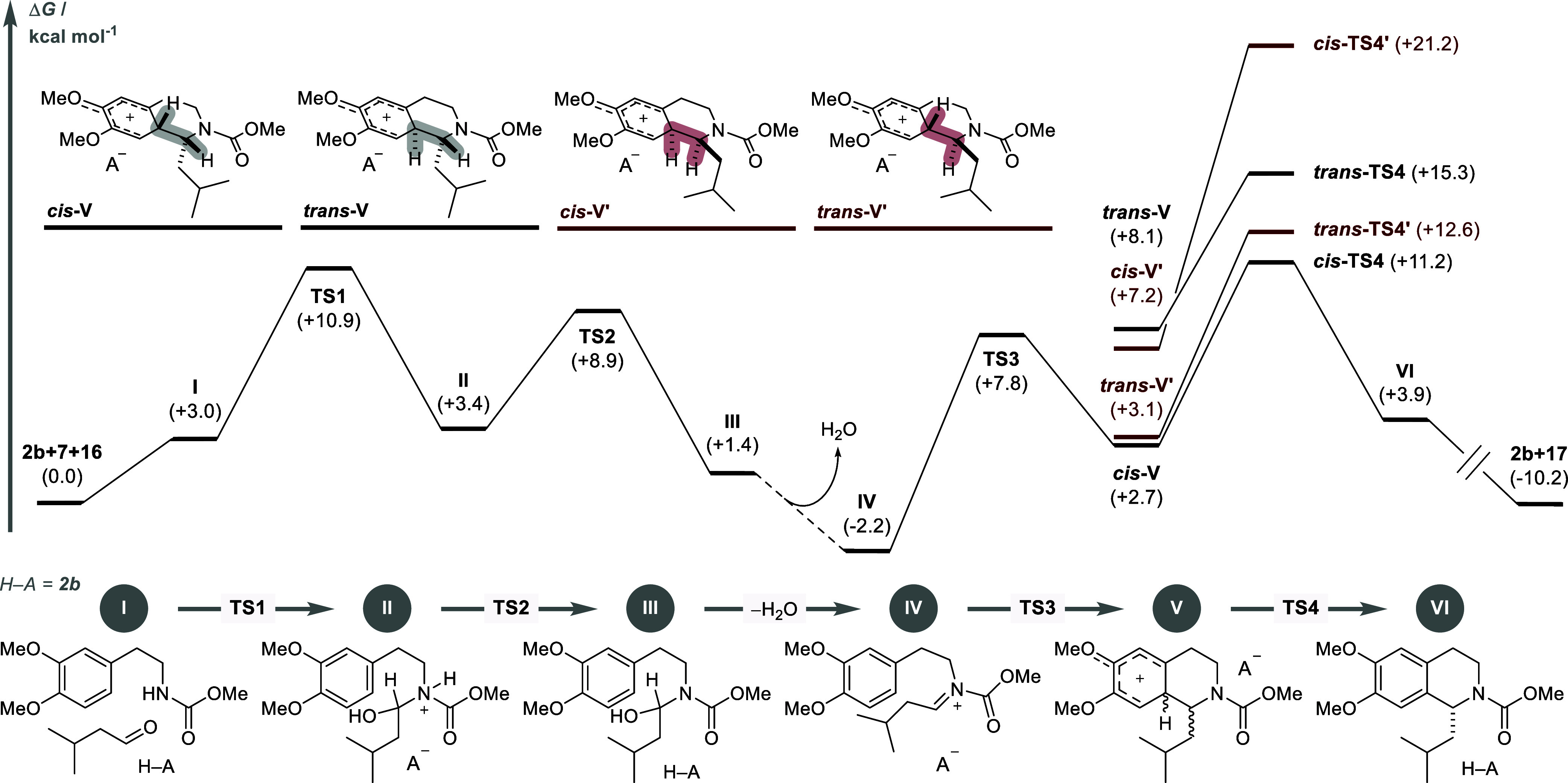
DFT calculations. Energy diagram of the reaction mechanism, calculated at CPCM(CHCl_3_)-ωB97M-V/(ma)-def2-TZVPP//r^2^SCAN-3c level of theory. Thermal corrections were calculated at 298.15 K. The energy profile leading to the major enantiomer is depicted in black, and the one leading to the minor enantiomer in red. The four possible stereoisomers of intermediate **V** are illustrated for clarity.

To gain further insights into the reaction mechanism, we conducted Density Functional Theory (DFT) calculations at the CPCM(CHCl_3_)-ωB97M-V/(ma)-def2-TZVPP//r^2^SCAN-3c level of theory (Figure 11, see Supporting Information for details).^55−57^ We chose to examine the reaction of carbamate **7** with isovaleraldehyde (**16**) as a representative substrate combination. The reaction commences with protonation of **16** and subsequent nucleophilic addition of carbamate **7** to yield ion pair **II**. A subsequent proton transfer via **TS2** furnishes hemiaminal intermediate **III**. The following dehydration step generates the *N*-acyliminium ion pair **IV** in a local energy minimum. Intramolecular cyclization toward arenium ion **V** generates two adjacent stereogenic centers. Consequently, a pair of diastereomers, *cis***-** and *trans***-V**, and their enantiomers *cis***-** and *trans***-V′** in conjunction with the enantiopure IDPi counteranion were considered computationally. After deprotonation and rearomatization, the THIQ product is obtained as a complex with the IDPi catalyst (**VI**), which readily dissociates toward the free catalyst **2b** and the THIQ product **17**. Overall, the TS for the final deprotonation of arenium ion **V** is higher in energy than all previous steps, which renders **TS4** both enantio- and rate-determining. Consequently, all stereoisomers of **TS4** indeed have to be taken into account, due to the possible formation of **V** in a dynamic equilibrium. Through careful studies, *cis***-TS4** and *trans***-TS4′** appear to be the favored TSs, leading to the major and minor product enantiomers, respectively. The calculated energy difference between these TSs was 1.36 kcal mol^–1^, which corresponds to an enantiomeric ratio of 91:9 at 298 K. The calculated value is thus in good agreement with the experimentally observed ratio of 93.5:6.5.

Comparison of the respective ground states reveals that the two most stable isomers, *cis***-V** and *trans***-V′**, share the identical absolute configuration of the arenium ion component. The same is true for the lowest-lying TSs *cis***-TS4** and *trans***-TS4′**. It appears that the final C1 stereocenter has a smaller impact on the relative stability than the transient arenium ion stereocenter. This observation might contribute to the promiscuity of the optimal IDPi catalyst toward a large diversity of aldehyde reaction partners. In other words: The optimal IDPi catalyst does not occupy and destabilize the three-dimensional space of the aldehyde residue, but rather allows the substrate to “find” the most stable C1 configuration relative to the arenium stereocenter. Thus, substrate-inherent contributions such as distortion become dominant stereodetermining factors.

**Figure 12:**
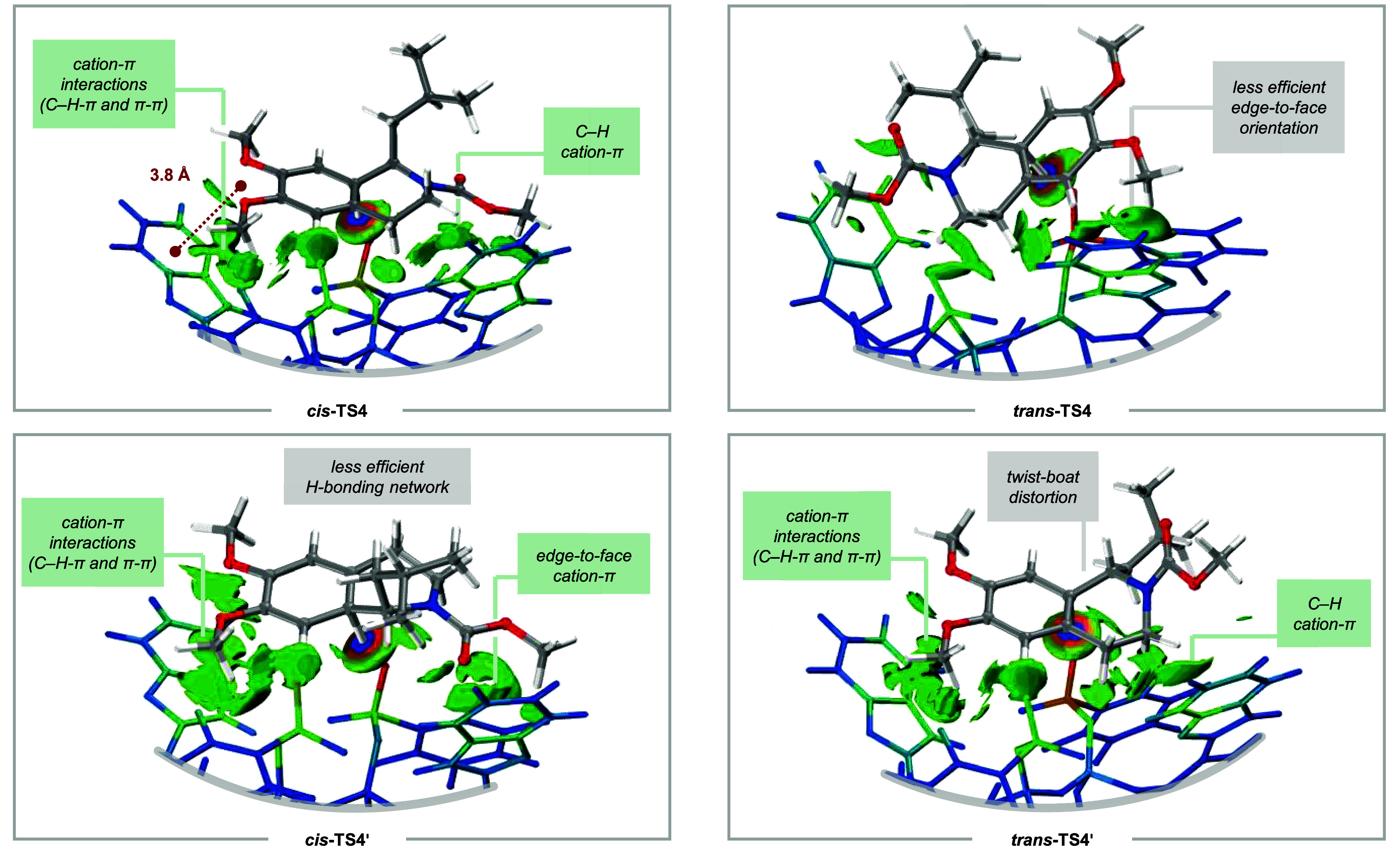
IGMH maps of the four stereoisomers of **TS4** (Isosurface = 0.005). Green isosurfaces visualize the NCIs between the substrate and the catalyst. Key attractive interactions are highlighted in the green text box, while plausible destabilizing factors are indicated in gray. The relative atomic contributions of the anion are colored by δ*G*^atom^. Brighter-colored atoms indicate stronger contributions to the interfragment interactions.

To comprehend the high enantioselectivity observed in the reactions in detail, we sought to visualize the relevant NCIs in **TS4** (Figure 12). We conducted a computational analysis according to the independent gradient model based on Hirshfeld partition (IGMH).^58^ We were thus able to illustrate the weak interfragment interactions between the substrate cation and the IDPi counteranion, as well as the individual atomic contributions to the NCIs. All isomers of **TS4** share elemental characteristics. The arenium ion is embedded in a complex hydrogen bonding network comprised of polarized C–H bonds of the substrate and Lewis basic residues of the counteranion. These H-bond acceptors reside in the inner core of the IDPi catalyst, which largely contributes to delocalization of the negative charge. The ultimate deprotonation occurs from one of two diastereotopic sulfonimidate oxygens, the topicity of which is identical in all calculated stereoisomers. Notably, the conformational freedom of the sulfonimidate residues in the counteranion is restricted by strong face-to-face cation–π interactions between the perfluorinated core and the electron-rich BINOL backbone (see the Supporting Information for full structures). Due to their substantial involvement in the stereodetermining deprotonation, this geometrical lock likely contributes to the pronounced effect on the enantioselectivity upon exchange of the CF_3_-groups in IDPi catalyst **2a** to C_6_F_5_-groups in **2b**.

As illustrated, the favored TS *cis***-TS4** is stabilized by multiple NCIs. Beyond electrostatic contributions arising from nonobvious hydrogen bonding between polarized C–H bonds and basic residues of the counteranion, substantial cation–π interactions can be found between two benzofuran substituents and both the arenium ion and the piperidine ring. The arenium ion and one benzofuran substituent are oriented in a parallel-displaced face-to-face geometry, and a distance of 3.8 Å was measured between the benzofuran ring center and the imaginable center between the two methoxy substituents. This contact is well in line with the considerations in the study of NCIs in structural biology and small molecule catalysis.^59^ Importantly, the two most stable TSs, *cis***-TS4** and *trans***-TS4′**, seem to share comparable stabilizing NCIs. The enantiodetermining energy difference however arises from an energy penalty due to catalyst and substrate distortion into a twist-boat conformation to maximize the stabilizing interactions within the ion pair, as was further supported by distortion–interaction analysis (see the Supporting Information for details).^60^ The energetically less favored isomers *trans***-TS4** and *cis***-TS4′** are both destabilized, likely by a lack of attractive forces such as favorable cation–π interactions in parallel-displaced orientation, and a crucial H-bond to a nitrogen of the anion, respectively.

### 2.8 Reaction Optimization for 1-Aryl THIQs

**Figure 13:**
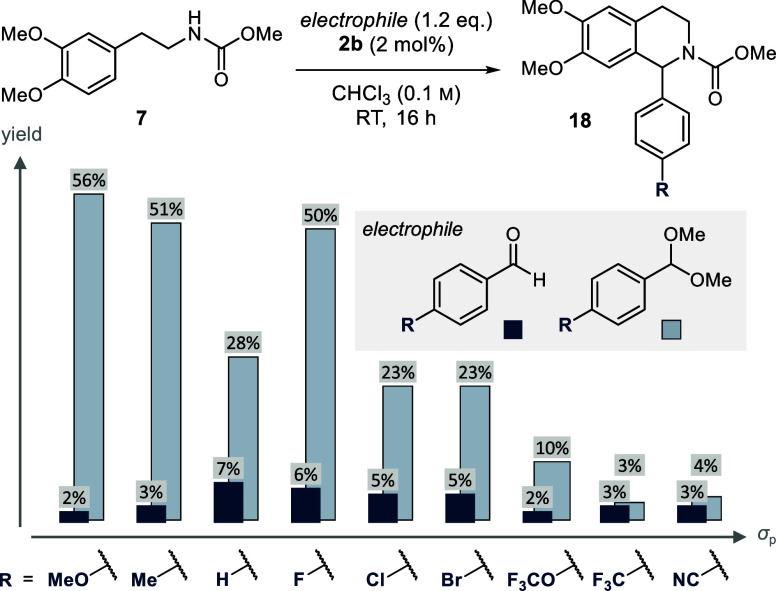
Reactivity assessment in the reaction toward 1-aryl THIQs **18** by employing aldehydes and dimethyl acetals as electrophiles. Comparison of *para*-substituted electrophiles with diverse electronic properties according to their respective Hammett coefficient σ_p_. Yields were determined by ^1^H NMR spectroscopy of the crude reaction mixture using CHPh_3_ as internal standard.

Previously, when we applied the optimal reaction conditions for the Pictet–Spengler reaction of aliphatic aldehydes^29^ to the transformation of substituted benzaldehydes, we saw a significant decrease in reactivity to an extend that rendered the reaction synthetically impractical (<10% yield). We rationalized that the mesomeric stabilization of both a protonated aromatic aldehyde as well as an aromatic *N*-acyliminium ion leads to a considerable reduction of their respective electrophilicity.^61^ Our mechanistic insights into the reaction profile of aliphatic aldehydes led us to conclude that the cyclization event is unlikely to be rate-determining in the reactions under study. Consequently, the electrophilicity of the *N*-acyliminium ion must be irrelevant to the overall rate. Instead, we suspected the formation of the hemiaminal intermediate by nucleophilic attack of the carbamate to be the bottleneck in the transformation of benzaldehydes. We therefore hypothesized that high reactivity might be restored by masking the aldehyde as the corresponding dimethylacetal. The formation of a highly electrophilic alkyl carbonylonium ion intermediate should increase the rate of nucleophilic attack. Importantly, the downstream intermediates in the catalytic cycle remain similar or identical by this substrate modification. Furthermore, the condensation event would be entropically favored by liberation of two equivalents of MeOH. In order to test our hypothesis, we examined the reactivity profile of electronically diverse *para*-substituted benzaldehydes and their respective dimethylacetal derivatives in the Pictet–Spengler reaction with carbamate **7** (Figure 13).

The reactivity of the aldehydes under study reached a maximum for the electron-neutral and slightly electron-poor substrates (R = H, F, Cl, Br), although on a low-yielding plateau (≤7% yield). Both electron-donating as well as electron-withdrawing substituents further decreased the reactivity. We were however pleased to observe superior product yields, when dimethylacetals were utilized as reaction partners. The reactivity enhancement was particularly pronounced in the case of electron-rich substrates (R = OMe, Me), further supporting our hypothesis that the electrophilicity ought to be enhanced to facilitate reaction progress. Nevertheless, an electronic substrate bias was still apparent, as electron-poor acetals (R = CF_3_, CN) remained poorly reactive.

**Figure 14:**
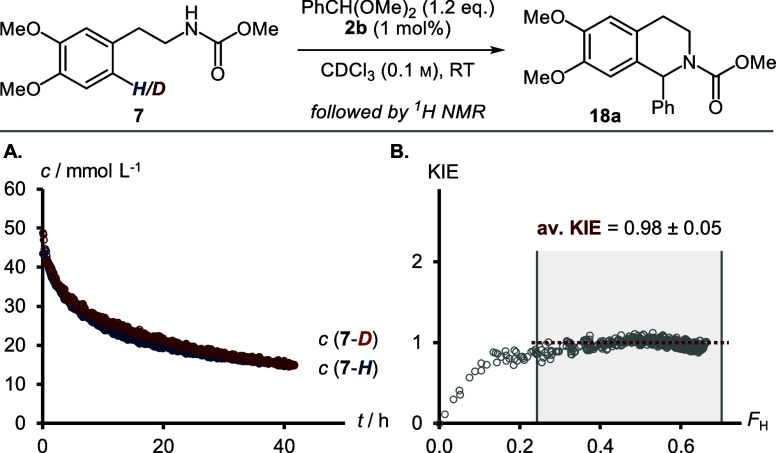
Competition KIE experiment using partly deuterated substrate **7** and benzaldehyde dimethylacetal. (A) Concentration profiles. (B) Calculated KIE according to eq 1. The average KIE was determined at fractional conversion of the protonated starting material *F*_H_ > 0.25. The error is given as the standard deviation.

As a consequence of the reactivity-enhancement achieved by employing dimethylacetals, the relative reaction rates in the mechanism have likely been altered. We were interested in probing, whether or not the final deprotonation remains the rate-limiting step, as previously seen for aliphatic aldehydes (see Figure 6). We therefore repeated the competition KIE experiment with partly deuterated carbamate **7** and benzaldehyde dimethylacetal (Figure 14). In sharp contrast to the reaction with hexanal, the concentration profiles of deuterated and nondeuterated substrate **7** progress almost identically. This observation is reflected in a calculated average KIE of 0.98 ± 0.05. In agreement with our data, we hypothesize that the RDS for aromatic dimethylacetals is indeed most likely the initial formation of an alkyl carbonylonium ion, followed by an irreversible nucleophilic attack of carbamate **7**.

**Figure 15:**
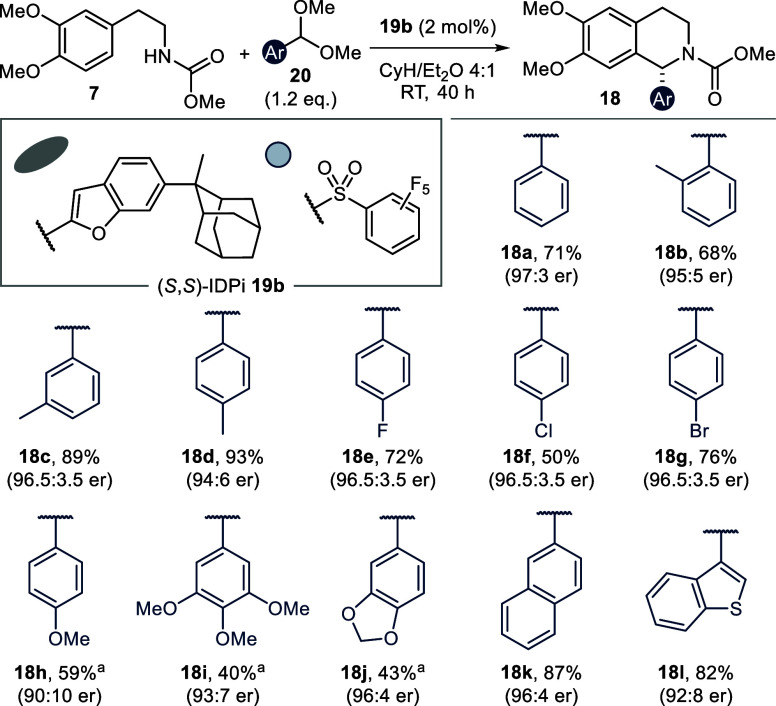
Scope of 1-aryl THIQs **18**. All reactions were conducted on a 0.10 mmol scale. Yields are reported as isolated yields after column chromatography. ^a^Reaction was performed at 0 °C for 72 h. See the Supporting Information for detailed reaction conditions.

After reoptimization of the reaction conditions (see the Supporting Information for further details), we found that high reactivity and selectivity could be achieved by performing the reaction in a nonpolar solvent mixture (CyH/Et_2_O 4:1). Importantly, the same 2-methyladamantyl-decorated IDPi **19b**, which was ideal for the scope of aliphatic aldehydes,^29^ remained optimal for aromatic dimethylacetals as well. With an improved reaction protocol at hand, we explored the scope of amenable acetals **20** in the synthesis of 1-aryl THIQs **18** (Figure 15). The absolute configuration of the obtained products was assigned in analogy to the aliphatic THIQs.^29^

The parent unsubstituted product **18a** was obtained in good yield and with excellent selectivity (71%, 97:3 er). Additionally, methyl substituents in the *ortho*-, *meta*-, or *para*-position were well tolerated (**18b**–**d**). Halogenation could be handled by the optimal catalyst (**18e**–**g**), even though *para*-chloro THIQ **18f** was formed in slightly reduced yield. 1-Aryl THIQs are a rare structural motif in natural products. Nevertheless, (poly)alkoxylated alkaloids of this type have been isolated from *cryptostylis fulva* and *cryptostylis erythroglossa* orchids.^62,63^ When we tested the relevant electron-rich substitution patterns, we however encountered an unexpected problem of product racemization (see the Supporting Information for details). THIQ products **18h**–**j** could nevertheless be obtained in moderate yields but with high enantiopurity, if the synthesis was conducted at reduced reaction temperature. Finally, 2-naphthyl- and 3-benzothiophenyl-substituted THIQs **18k** and **18L** were obtained in high efficiency and with good enantioselectivity.

## 3 Summary

2-Benzofuranyl-substituted IDPi catalysts offer a unique reactivity and selectivity profile in catalytic asymmetric Pictet–Spengler reactions across a range of substrate classes. First and foremost, we noticed an unexpected but significant acidifying effect upon installation of the 2-benzofuranyl substituents in IDPi catalysts, which undoubtedly accounts in part for the observed high reactivity. Additionally, we present evidence for the involvement of attractive noncovalent interactions between the catalysts and the high-energy cationic intermediates and TSs formed in the catalytic cycle. Specifically, we examined the π-donor capabilities of benzofuran substituents in cation–π interactions within key ion pairs both experimentally and computationally. We suspect that our findings will be inspirational to catalyst development campaigns in the field of ACDC in general. Our deepened understanding of the reaction kinetics in catalytic asymmetric Pictet–Spengler reactions furthermore led us to the identification of improved reaction conditions for the construction of 1-aryl tetrahydroisoquinolines. This substrate class is now synthetically available for the first time from the corresponding aryl dimethylacetals with high catalytic enantiocontrol.

## Supporting Information Available

Experimental details and analytical data for all new compounds (ZIP)
NMR kinetic data (ZIP)
Cartesian coordinates and IGMH analysis (PDF)
