# Supplementary material for: Highly Acidic Electron-Rich Brønsted Acids Accelerate Asymmetric Pictet–Spengler Reactions by Virtue of Stabilizing Cation–π Interactions
Source: J Am Chem Soc. 2024 Oct 3;146(41):28339–49. doi: 10.1021/jacs.4c09421 (PMC11487569; doi:10.1021/jacs.4c09421)
Supplement: Supplementary file 3 — ja4c09421_si_003.pdf [file ja4c09421_si_003.pdf]

Highly Acidic Electron-Rich Brønsted Acids Accelerate Asymmetric Pictet–Spengler Reactions by Virtue of Stabilizing Cation-*π* Interactions

Manuel J. Scharf[a], Nobuya Tsuji[b], Monika M. Lindner[a], Markus Leutzsch[a], Märt Lõkov[c], Elisabeth Parman[c], Ivo Leito[c], Benjamin List[a,b]*

[a]Max-Planck-Institut für Kohlenforschung, 45470 Mülheim an der Ruhr, Germany

[b]Institute for Chemical Reaction Design and Discovery, Hokkaido University, Sapporo 001-0021, Japan

[c]Institute of Chemistry, University of Tartu, 50411 Tartu, Estonia

*Email: list@kofo.mpg.de

Contents

1. General Information 1
2. Asymmetric Pictet–Spengler Reactions 2
3. Synthesis of Protected *β*-Arylethylamines 10
4. Synthesis of Aldehydes and Acetals 13
5. Synthesis of IDPi Catalysts 13
6. Acidity Measurements 14
7. NMR Kinetics 21
8. Computational Studies 33
9. NMR Spectra 38
10. HPLC Traces 62
11. References 74

1. General Information

Unless otherwise stated, all reagents were purchased from commercial suppliers and used without further purification. All solvents used in the reactions were distilled from appropriate drying agents prior to use. Reactions were monitored by thin layer chromatography (TLC) on silica gel pre-coated glass (0.2 mm, Macherey-Nagel). Visualization was accomplished by irradiation with UV light at 254 nm and/or cerium ammonium molybdate (CAM) stain and/or $KMnO_4$ stain. Column chromatography was carried out using Merck (60 Å, 230−400 mesh, particle size 0.040−0.063 mm) or VWR (40−63 µm) silica gel, using technical grade solvents. Automated reversed phase column chromatography was conducted on a Biotage Isolera Spektra Four system, using SNAP Ultra C18 HP-Sphere 25 µm reversed phase cartridges. All reported yields refer to chromatographically and spectroscopically pure compounds. ^{1}H and ^{13}C NMR spectra were recorded on a Bruker AV-500 NMR, a Bruker AVNeo 600 MHz NMR, or a AVIII HD 400 MHz NMR spectrometer in deuterated solvents. ^{1}H chemical shifts (δ) are reported in ppm relative to the protonated solvent resonance employed as the internal standard ($CDCl_3$ δ = 7.26, CD_2Cl_2 δ = 5.32, DMSO δ = 2.50, CD_3OD δ = 3.31 ppm). Data are reported as follows: chemical shift, multiplicity (s = singlet, d = doublet, t = triplet, q = quartet, p = pentet, s = sextet, h = heptet, m = multiplet, b = broad), coupling constants (Hz), and integration. ^{13}C chemical shifts are reported in ppm with the solvent resonance as the internal standard ($CDCl_3$ δ = 77.16,

CD_2Cl_2 δ = 54.00, DMSO δ = 39.52, CD_3OD δ = 49.00 ppm). High resolution mass spectra were determined on a Bruker APEX III FTMS (7 T magnet). Optical rotations were determined with an Autopol IV polarimeter (Rudolph Research Analytical) at 589 nm and 25 °C. Data are reported as follows: α_{λ}^{T}, concentration *c* (g/100 mL), and solvent. Enantiomeric ratios (er) were determined by HPLC analysis employing a chiral stationary phase column specified in the individual experiment, by comparing the samples with the corresponding racemic mixtures.

2. Asymmetric Pictet–Spengler Reactions

Reaction Optimization

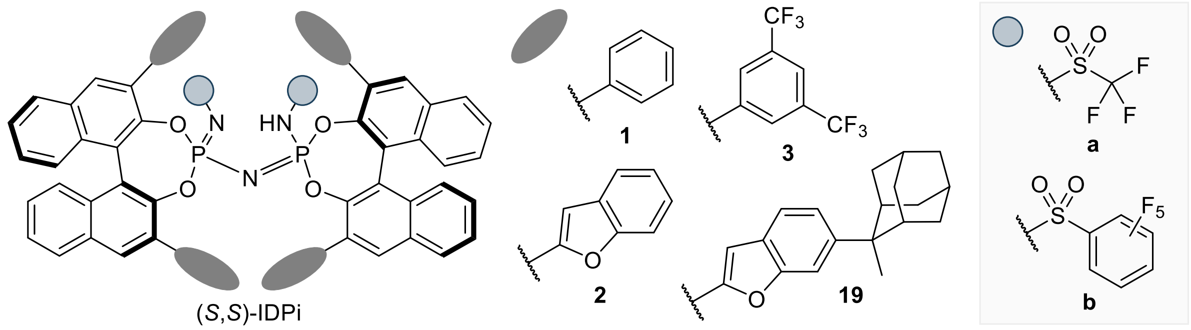

Figure S1 Catalysts used in the reaction optimization.

An oven-dried GC vial equipped with a magnetic stir bar was charged with the catalyst (2 mol%) and placed under argon. Substrate (0.025 mmol) and aldehyde or acetal (1.2 eq.) were dissolved separately in the reaction solvent and sequentially added to the catalyst. The mixture was then stirred at the appropriate temperature for 16 h (for reactions at reduced *T*, the reaction was started at –78 °C and then warmed to the reaction temperature). The reaction was quenched by addition of Et_3N (10 µL) followed by addition of Ph_3CH as internal standard (1.0 M in PhMe, 25 µL, 1.0 eq.). $CDCl_3$ (0.5 mL) was added to the mixture, and 0.5 mL were analyzed by ^1H NMR to determine the product yield. The remaining solution was purified by preparative thin layer chromatography to give the enantiomeric ratio after HPLC analysis.

Table S1 Reaction Optimization for aromatic dimethylacetals.

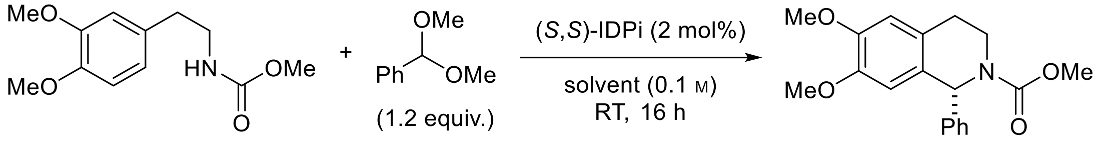

entry	(*S*,*S*)-IDPi	solvent	time	yield	er
1	**2b**	$CHCl_3$	40 h	51%	90:10
2	**19b**	$CHCl_3$	40 h	49%	95:5
3	**19b**	$CHCl_3$	16 h	26%	94.5:5.5
4	**19b**	CH_2Cl_2	16 h	36%	83:17
5	**19b**	PhMe	16 h	40%	96:4
6	**19b**	Et_2O	16 h	79%	93.5:6.5
7	**19b**	THF	16 h	26%	89:11
8	**19b**	CyH	16 h	36%	97.5:2.5
9	**19b**	Et_2O/CyH 9:1	16 h	82%	94.5:5.5
10	**19b**	Et_2O/CyH 4:1	16 h	78%	95:5
11	**19b**	Et_2O/CyH 1:1	16 h	73%	96:4
12	**19b**	Et_2O/CyH 1:4	16 h	63%	97:3
13	**19b**	Et_2O/CyH 1:9	16 h	49%	97.5:2.5

The synthesis of electron-rich substrates **18h-j** was further optimized, due to an observed reduced enantioselectivity under the standard reaction conditions (Table S2). We propose a racemization pathway via acid-mediated ring-opening of the tetrahydroisoquinoline, which generates a bis-benzylic carbocation that is electronically further stabilized by electron-donating substituents. This racemization pathway could be confirmed by reaction of enantioenriched **18h** with catalytic amounts of $HNTf_2$, which leads to facile loss of enantiopurity (not shown).

Table S2 Proposed racemization pathway for electron-rich 1-aryl tetrahydroisoquinolines and re-optimization of the reaction conditions for substrate **18h**.

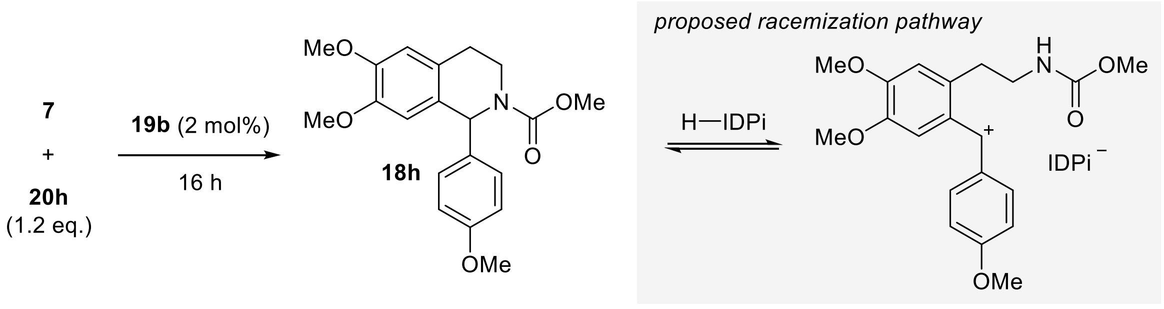

**entry**	**solvent**	**T**	**yield**	**er**
1	$CHCl_3$	RT	56%	63:37
2	CyH	RT	65%	77:23
3	$CHCl_3$	0 °C	23%	95:5
4	CyH	0 °C	4%	94:6
5	Et_2O	0 °C	52%	72:28
6	Et_2O/CyH 1:4	0 °C	30%	93.5:6.5

Synthesis of 1-aryltetrahydroisoquinolines

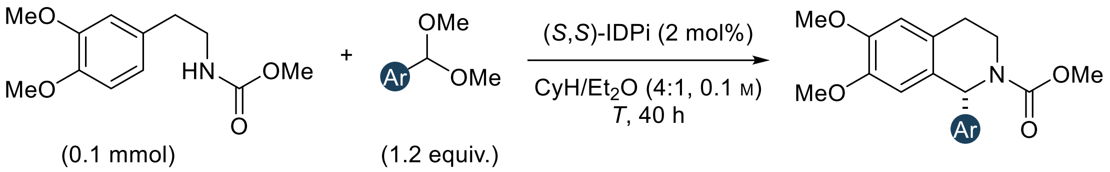

General procedure a (liquid aromatic acetals)

Carbamate **7** (23.9 mg, 0.10 mmol, 1.00 eq.) and (S,S)-IDPi catalyst **19b** (4.39 mg, 0.002 mmol, 2 mol%) were weighed into a septum-capped 4 mL vial equipped with a magnetic stir bar, placed under argon, and dissolved in dry CyH (0.8 mL) and Et_2O (0.2 mL). The corresponding acetal **20** (0.12 mmol, 1.2 eq.) was subsequently added *via* Hamilton syringe, the vial was sealed with Parafilm©, and the mixture was stirred at RT for 40 h. The reaction was quenched by addition of 5 drops of Et_3N and concentrated on silica. The product was isolated by silica gel flash column chromatography. **for deviations from the general procedure, see the corresponding entries.*

General procedure b (solid aromatic acetals)

Carbamate **7** (23.9 mg, 0.10 mmol, 1.00 eq.) and (S,S)-IDPi catalyst **19b** (4.39 mg, 0.002 mmol, 2 mol%) were weighed into a septum-capped 4 mL vial equipped with a magnetic stir bar, placed under argon, and suspended in dry CyH (0.8 mL). The corresponding acetal **20** (0.18 mmol, 1.8 eq.) was placed in an oven-dried GC-vial, argonated, and dissolved in dry Et_2O (0.3 mL). Of the thus prepared acetal stock solution, 0.2 mL (0.12 mmol, 1.2 eq.) were added to the substrate and catalyst under argon, the vial was sealed with Parafilm©, and the mixture was stirred at RT for 40 h. The reaction was quenched by addition of 5 drops of Et_3N and concentrated on silica. The product was isolated by silica gel flash column chromatography. **for deviations from the general procedure, see the corresponding entries.*

Racemate synthesis

The corresponding racemates for determination of the enantiomeric access by HPLC-analysis were synthesized by reacting carbamate (0.1 mmol) with aldehyde (1.2 mmol) and catalytic amounts of Tf_2NH (7.5 mol%) in dry CH_3CN (1.0 mL) for 16 h.

Methyl 6,7-dimethoxy-1-phenyl-3,4-dihydroisoquinoline-2(1H)-carboxylate (18a)

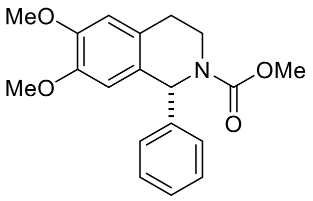

The reaction was performed according to **general procedure a** with benzaldehyde dimethylacetal (**20a**, 18 µL, 0.12 mmol, 1.2 eq.). Purification by silica gel flash column chromatography (DCM/EtOAc 3–7.5%) afforded the product as a white foam (23.3 mg, 71 µmol, 71%).

***R*F** (DCM/EtOAc 19:1) = 0.44.

^{1}H NMR (501 MHz, CD_3CN): δ = 7.33–7.28 (m, 2H), 7.28–7.20 (m, 3H), 6.77 (s, 1H), 6.63 (s, 1H), 6.24 (s, 1H), 3.89 (s, 1H), 3.79 (s, 3H), 3.69 (s, 3H), 3.66 (s, 3H), 3.21 (s, 1H), 2.84 (ddd, J = 16.2, 10.3, 5.9 Hz, 1H), 2.68 (dt, J = 16.1, 4.3 Hz, 1H).

^{13}C NMR (126 MHz, CD_3CN): δ = 156.87, 149.36, 148.68, 144.13, 129.24, 129.00, 128.28, 128.22, 128.11, 112.80, 112.46, 58.25, 56.41, 56.37, 53.18, 39.19, 28.42.

EI HRMS: calculated for $C_{19}H_{21}N_1O_4$ ($[M]^+$): 327.146509, found: 327.146690.

HPLC (IA-3, *n*-heptane/*i*-PrOH 80:20, 298 K, 283 nm): t_R (major) = 9.1 min, t_R (minor) = 11.7 min, er = 97:3 (94% ee).
$[\alpha]_D^{25}$ = −168.8 (*c* = 0.25, $CHCl_3$).

Methyl 6,7-dimethoxy-1-(*o*-tolyl)-3,4-dihydroisoquinoline-2(1*H*)-carboxylate (18b)

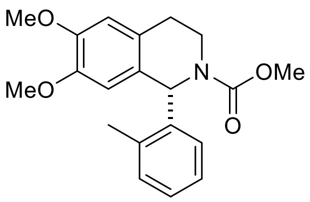

The reaction was performed according to **general procedure a** with 2-methylbenzaldehyde dimethylacetal (**20b**, 20 µL, 0.12 mmol, 1.2 eq.). Purification by silica gel flash column chromatography (DCM/EtOAc 10%) afforded the product as a white foam (23.1 mg, 68 µmol, 68%).
***R*F** (hex/EtOAc 2:1) = 0.43.
^{1}H NMR (501 MHz, CD_3CN): δ = 7.23 (d, J = 7.4 Hz, 1H), 7.16 (td, J = 7.4, 1.4 Hz, 1H), 7.07–7.01 (m, 1H), 6.76 (s, 1H), 6.72 (d, J = 7.8 Hz, 1H), 6.45 (s, 1H), 6.39 (s, 1H), 3.95 (s, 1H), 3.79 (s, 3H), 3.66 (s, 3H), 3.61 (s, 3H), 3.11 (ddd, J = 13.6, 12.1, 4.4 Hz, 1H), 2.92 (ddd, J = 18.0, 12.1, 6.2 Hz, 1H), 2.72–2.62 (m, 1H), 2.51 (s, 3H).
^{13}C NMR (126 MHz, CD_3CN): δ = 157.10, 149.32, 148.74, 141.96, 138.47, 131.54, 130.52, 128.83, 128.41, 128.18, 126.42, 112.99, 112.00, 56.36, 56.32, 55.97, 53.25, 38.72, 28.04, 19.87.
EI HRMS: calculated for $C_{20}H_{23}N_1O_4$ ($[M]^+$): 341.162159, found: 341.162460.
HPLC (IA-3, *n*-heptane/*i*-PrOH 80:20, 298 K, 283 nm): t_R (major) = 7.0 min, t_R (minor) = 8.3 min, er = 95:5 (90% ee).
$[\alpha]_D^{25}$ = −225.5 (*c* = 0.33, $CHCl_3$).

Methyl 6,7-dimethoxy-1-(*m*-tolyl)-3,4-dihydroisoquinoline-2(1H)-carboxylate (18c)

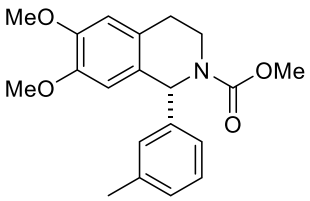

The reaction was performed according to **general procedure a** with 3-methylbenzaldehyde dimethylacetal (**20c**, 20 µL, 0.12 mmol, 1.2 eq.). Purification by silica gel flash column chromatography (DCM/EtOAc 10%) afforded the product as a white foam (30.4 mg, 89 µmol, 89%).
***R*F** (hex/EtOAc 2:1) = 0.39.
^{1}H NMR (501 MHz, CD_3CN): δ = 7.18 (t, J = 7.6 Hz, 1H), 7.10–7.03 (m, 2H), 7.00 (d, J = 7.6 Hz, 1H), 6.76 (s, 1H), 6.61 (s, 1H), 6.20 (s, 1H), 3.89 (s, 1H), 3.78 (s, 3H), 3.69 (s, 3H), 3.66 (s, 3H), 3.27–3.17 (m, 1H), 2.84 (ddd, J = 16.2, 10.4, 5.9 Hz, 1H), 2.74–2.64 (m, 1H), 2.28 (s, 3H).
^{13}C NMR (126 MHz, CD_3CN): δ = 156.79, 149.34, 148.66, 144.12, 138.95, 129.59, 129.14, 128.97, 128.21, 128.19, 126.13, 112.77, 112.46, 58.25, 56.41, 56.36, 53.17, 39.18, 28.43, 21.51.
EI HRMS: calculated for $C_{20}H_{23}N_1O_4$ ($[M]^+$): 341.162159, found: 341.162530.
HPLC (IA-3, *n*-heptane/*i*-PrOH 80:20, 298 K, 283 nm): t_R (major) = 8.0 min, t_R (minor) = 10.3 min, er = 96.5:3.5 (93% ee).
$[\alpha]_D^{25}$ = −172.0 (*c* = 0.34, $CHCl_3$).

Methyl 6,7-dimethoxy-1-(*p*-tolyl)-3,4-dihydroisoquinoline-2(1H)-carboxylate (18d)

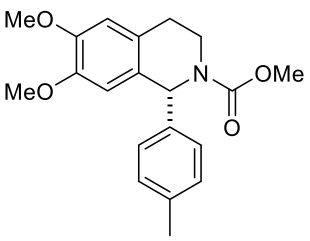

The reaction was performed according to **general procedure a** with 4-methylbenzaldehyde dimethylacetal (**20d**, 20 µL, 0.12 mmol, 1.2 eq.). Purification by silica gel flash column chromatography (DCM/EtOAc 5–10%) afforded the product as a white foam (31.9 mg, 93 µmol, 93%).

$\boldsymbol{R_F}$ (hex/EtOAc 2:1) = 0.39.

^{1}H NMR (501 MHz, CD_3CN): δ = 7.14–7.06 (m, 4H), 6.76 (s, 1H), 6.60 (s, 1H), 6.20 (s, 1H), 3.89 (s, 1H), 3.78 (s, 3H), 3.68 (s, 3H), 3.65 (s, 3H), 3.25–3.10 (m, 1H), 2.83 (ddd, J = 16.3, 10.5, 5.9 Hz, 1H), 2.67 (dt, J = 16.1, 4.1 Hz, 1H), 2.28 (s, 3H).

^{13}C NMR (126 MHz, CD_3CN): δ = 156.75, 149.31, 148.65, 141.16, 138.03, 129.83, 129.00, 128.25, 128.16, 112.78, 112.43, 57.97, 56.40, 56.36, 53.14, 39.00, 28.45, 21.07.

EI HRMS: calculated for $C_{20}H_{23}N_1O_4$ ($[M]^+$): 341.162159, found: 341.162470.

HPLC (IA-3, *n*-heptane/*i*-PrOH 80:20, 298 K, 283 nm): t_R (major) = 9.1 min, t_R (minor) = 10.7 min, er = 94:6 (88% ee).

$[\boldsymbol{\alpha}]_{\boldsymbol{D}}^{\boldsymbol{25}}$ = −163.2 (*c* = 0.44, $CHCl_3$).

Methyl 1-(4-fluorophenyl)-6,7-dimethoxy-3,4-dihydroisoquinoline-2(1H)-carboxylate (18e)

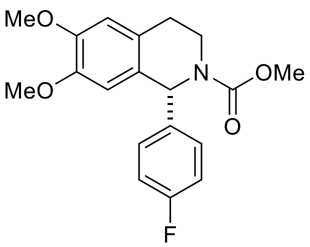

The reaction was performed according to **general procedure a** with 4-fluorobenzaldehyde dimethylacetal (**20e**, 19 µL, 0.12 mmol, 1.2 eq.). Purification by silica gel flash column chromatography (DCM/EtOAc 10%) afforded the product as a white foam (24.9 mg, 72 µmol, 72%).

$\boldsymbol{R_F}$ (hex/EtOAc 2:1) = 0.31.

^{1}H NMR (501 MHz, CD_3CN): δ = 7.23 (dd, J = 8.6, 5.7 Hz, 2H), 7.07–6.99 (m, 2H), 6.77 (s, 1H), 6.60 (s, 1H), 6.23 (s, 1H), 3.90 (s, 1H), 3.79 (s, 3H), 3.69 (s, 3H), 3.66 (s, 3H), 3.27–3.08 (m, 1H), 2.84 (ddd, J = 16.3, 10.5, 5.9 Hz, 1H), 2.67 (dt, J = 16.1, 4.2 Hz, 1H).

^{19}F NMR (471 MHz, CD_2Cl_2): δ = -114.96, -115.10 (mixture of rotamers).

^{13}C NMR (126 MHz, CD_3CN): δ = 163.87, 161.94, 156.80, 149.07 (d, *J* = 87.6 Hz), 140.29 (d, *J* = 3.2 Hz), 130.98 (d, *J* = 8.1 Hz), 128.21, 127.86, 115.80 (d, *J* = 21.4 Hz), 112.80, 112.39, 57.53, 56.40, 56.36, 53.21, 39.00, 28.41.

EI HRMS: calculated for $C_{19}H_{20}N_1O_4F_1$ ($[M]^+$): 345.137087, found: 345.137460.

HPLC (IA-3, *n*-heptane/*i*-PrOH 80:20, 298 K, 283 nm): t_R (major) = 9.6 min, t_R (minor) = 12.1 min, er = 96.5:3.5 (93% ee).

$[\boldsymbol{\alpha}]_{\boldsymbol{D}}^{\boldsymbol{25}}$ = −73.8 (*c* = 0.44, $CHCl_3$).

Methyl 1-(4-chlorophenyl)-6,7-dimethoxy-3,4-dihydroisoquinoline-2(1H)-carboxylate (18f)

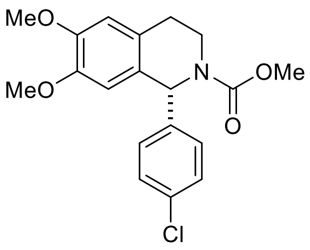

The reaction was performed according to **general procedure a** with 4-chlorobenzaldehyde dimethylacetal (**20f**, 20 µL, 0.12 mmol, 1.2 eq.). Purification by silica gel flash column chromatography (DCM/EtOAc 10%) afforded the product as a white foam (18.0 mg, 50 µmol, 50%).
***R*F** (hex/EtOAc 2:1) = 0.40.
^{1}H NMR (501 MHz, CD_3CN): δ = 7.33–7.28 (m, 2H), 7.25–7.17 (m, 2H), 6.77 (s, 1H), 6.61 (s, 1H), 6.21 (s, 1H), 3.89 (s, 1H), 3.78 (s, 3H), 3.69 (s, 3H), 3.66 (s, 3H), 3.22–3.13 (m, 1H), 2.84 (ddd, J = 16.1, 10.3, 5.8 Hz, 1H), 2.67 (dt, J = 16.1, 4.3 Hz, 1H).
^{13}C NMR (126 MHz, CD_3CN): δ = 156.93, 149.46, 148.74, 143.05, 133.54, 130.73, 129.20, 128.25, 127.58, 112.81, 112.37, 57.62, 56.41, 56.36, 53.25, 39.20, 28.38.
EI HRMS: calculated for $C_{19}H_{20}N_1O_4Cl_1$ ($[M]^+$): 361.107537, found: 361.107910.
HPLC (IA-3, *n*-heptane/*i*-PrOH 90:10, 298 K, 284 nm): t_R (major) = 16.3 min, t_R (minor) = 19.5 min, er = 96.5:3.5 (93% ee).
$[\alpha]_D^{25}$ = −164.0 (*c* = 0.27, $CHCl_3$).

Methyl 1-(4-bromophenyl)-6,7-dimethoxy-3,4-dihydroisoquinoline-2(1H)-carboxylate (18g)

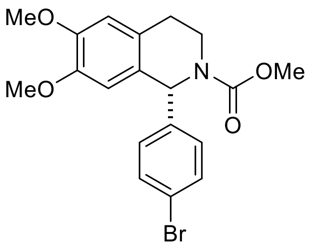

The reaction was performed according to **general procedure a** with 4-bromobenzaldehyde dimethylacetal (**20g**, 20 µL, 0.12 mmol, 1.2 eq.). Purification by silica gel flash column chromatography (DCM/EtOAc 10%) afforded the product as a white foam (30.8 mg, 76 µmol, 76%).
***R*F** (hex/EtOAc 2:1) = 0.38.
^{1}H NMR (501 MHz, CD_3CN): δ = 7.47–7.41 (m, 2H), 7.18–7.11 (m, 2H), 6.76 (s, 1H), 6.61 (s, 1H), 6.20 (s, 1H), 3.89 (s, 1H), 3.78 (s, 3H), 3.68 (s, 3H), 3.66 (s, 3H), 3.24–3.09 (m, 1H), 2.83 (ddd, J = 16.2, 10.3, 5.8 Hz, 1H), 2.66 (dt, J = 16.1, 4.3 Hz, 1H).
^{13}C NMR (126 MHz, CD_3CN): δ = 156.89, 149.46, 148.74, 143.51, 132.20, 131.07, 128.24, 127.49, 121.68, 112.80, 112.36, 57.68, 56.41, 56.36, 53.25, 39.21, 28.37.
ESI HRMS: calculated for $C_{19}H_{20}Br_1N_1Na_1O_4$ ($[M+Na]^+$): 428.04679, found: 428.04678.
HPLC (IA-3, *n*-heptane/*i*-PrOH 90:10, 298 K, 284 nm): t_R (major) = 16.3 min, t_R (minor) = 19.5 min, er = 96.5:3.5 (93% ee).
$[\alpha]_D^{25}$ = −150.2 (*c* = 0.27, $CHCl_3$).

Methyl 6,7-dimethoxy-1-(4-methoxyphenyl)-3,4-dihydroisoquinoline-2(1H)-carboxylate (18h)

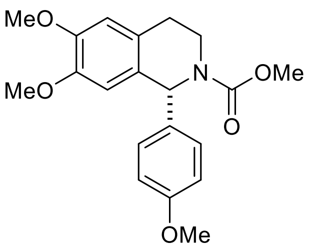

The reaction was performed according to **general procedure a** at 0 °C for 72 h with 4-methoxybenzaldehyde dimethylacetal (**20h**, 20.5 µL, 0.12 mmol, 1.2 eq.). Purification by silica gel flash column chromatography (DCM/EtOAc 10%) afforded the product as a white foam (21.2 mg, 59 µmol, 59%).
***R*F** (hex/EtOAc 1:1) = 0.51.

^{1}H NMR (501 MHz, CD_3CN): δ = 7.15–7.09 (m, 2H), 6.88–6.80 (m, 2H), 6.76 (s, 1H), 6.58 (s, 1H), 6.19 (s, 1H), 3.91 (s, 1H), 3.78 (s, 3H), 3.74 (s, 3H), 3.68 (s, 3H), 3.65 (s, 3H), 3.22–3.07 (m, 1H), 2.83 (ddd, J = 16.5, 10.8, 6.0 Hz, 1H), 2.67 (dt, J = 16.1, 4.0 Hz, 1H).
^{13}C NMR (126 MHz, CD_3CN): δ = 159.93, 156.82, 149.29, 148.65, 136.22, 130.32, 128.34, 128.14, 114.48, 112.78, 112.41, 57.62, 56.39, 56.36, 55.87, 53.13, 38.77, 28.46.
EI HRMS: calculated for $C_{20}H_{23}N_1O_5$ ($[M]^+$): 357.157074, found: 357.157540.
HPLC (IA-3, *n*-heptane/*i*-PrOH 70:30, 298 K, 283 nm): t_R (major) = 8.9 min, t_R (minor) = 10.3 min, er = 90:10 (80% ee).
$[\alpha]_D^{25}$ = −150.5 (*c* = 0.30, $CHCl_3$).

Methyl 6,7-dimethoxy-1-(3,4,5-trimethoxyphenyl)-3,4-dihydroisoquinoline-2(1H)-carboxylate (18i)

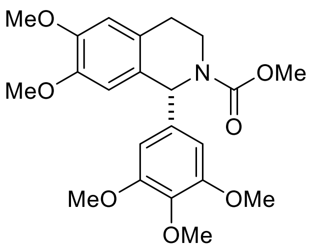

The reaction was performed according to **general procedure b** at 0 °C for 72 h with 3,4,5-trimethoxybenzaldehyde dimethylacetal (**20i**, 45.9 mg, 0.18 mmol, 1.8 eq.). Purification by silica gel flash column chromatography (hex/EtOAc 1:1) and preparative silica gel thin layer chromatography (hex/EtOAc 60:40) afforded the product as a white foam (16.7 mg, 40 µmol, 40%).
***R*F** (hex/EtOAc 1:1) = 0.27.
^{1}H NMR (501 MHz, CD_3CN): δ = 6.76 (s, 1H), 6.65 (s, 1H), 6.49 (s, 2H), 6.15 (s, 1H), 3.90 (s, 1H), 3.79 (s, 3H), 3.71 (s, 6H), 3.70 (s, 3H), 3.69 (s, 6H), 3.29 (q, J = 8.2 Hz, 1H), 2.84 (ddd, J = 16.0, 10.2, 5.8 Hz, 1H), 2.72 (dt, J = 16.1, 4.3 Hz, 1H).
^{13}C NMR (126 MHz, CD_3CN): δ = 154.09, 149.37, 148.67, 139.99, 138.22, 128.28, 127.99, 112.72, 112.47, 106.50, 60.79, 58.43, 56.65, 56.46, 56.34, 53.20, 39.43, 28.49.
ESI HRMS: calculated for $C_{22}H_{27}N_1Na_1O_7$ ($[M+Na]^+$): 440.16797, found: 440.16812.
HPLC (IA-3, *n*-heptane/*i*-PrOH 60:40, 298 K, 282 nm): t_R (minor) = 10.4 min, t_R (major) = 19.6 min, er = 93:7 (86% ee).
$[\alpha]_D^{25}$ = −118.1 (*c* = 0.19, $CHCl_3$).

Methyl 1-(benzo[*d*][1,3]dioxol-5-yl)-6,7-dimethoxy-3,4-dihydroisoquinoline-2(1H)-carboxylate (18j)

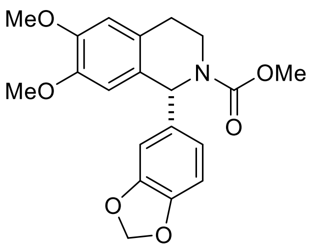

The reaction was performed according to **general procedure b** at 0 °C for 72 h with benzo[*d*][1,3]dioxole-5-carbaldehyde dimethylacetal (**20j**, 37.2 mg, 0.18 mmol, 1.8 eq.). Purification by silica gel flash column chromatography (DCM/EtOAc 9:1) afforded the product as a white foam (16.0 mg, 43 µmol, 43%).
***R*F** (hex/EtOAc 2:1) = 0.53.
^{1}H NMR (501 MHz, CD_3CN): δ = 6.76–6.72 (m, 3H), 6.70–6.64 (m, 1H), 6.59 (s, 1H), 6.15 (s, 1H), 5.92 (q, J = 1.1 Hz, 2H), 3.91 (s, 1H), 3.78 (s, 3H), 3.68 (s, 3H), 3.66 (s, 3H), 3.26–3.12 (m, 1H), 2.82 (ddd, J = 16.5, 10.7, 6.0 Hz, 1H), 2.67 (dt, J = 16.2, 4.0 Hz, 1H).
^{13}C NMR (126 MHz, CD_3CN): δ = 148.36, 147.68, 147.65, 146.81, 137.19, 127.15, 127.12, 121.61, 111.76, 111.40, 108.47, 107.59, 101.36, 56.92, 55.39, 55.36, 52.16, 37.83, 27.42. *The carbamate carbon signal could not be detected due to peak broadening.*
EI HRMS: calculated for $C_{20}H_{21}N_1O_6$ ($[M]^+$): 371.136339, found: 371.136810.

HPLC (IA-3, *n*-heptane/*i*-PrOH 60:40, 298 K, 283 nm): t_R (major) = 9.0 min, t_R (minor) = 10.5 min, er = 96:4 (92% ee).
$[\alpha]_D^{25}$ = −172.6 (*c* = 0.12, $CHCl_3$).

Methyl 6,7-dimethoxy-1-(naphthalen-2-yl)-3,4-dihydroisoquinoline-2(1H)-carboxylate (18k)

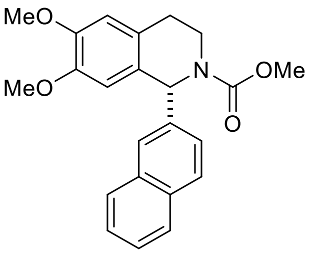

The reaction was performed according to **general procedure a** with 2-naphthaldehyde dimethylacetal (**20k**, 22 µL, 0.12 mmol, 1.2 eq.). Purification by silica gel flash column chromatography (DCM/EtOAc 10%) afforded the product as a white foam (32.7 mg, 87 µmol, 87%).
***R*F** (hex/EtOAc 2:1) = 0.35.
^{1}H NMR (501 MHz, CD_3CN): δ = 7.82 (dd, J = 9.0, 6.1 Hz, 2H), 7.79–7.74 (m, 1H), 7.57 (d, J = 1.8 Hz, 1H), 7.51–7.42 (m, 3H), 6.80 (s, 1H), 6.69 (s, 1H), 6.40 (s, 1H), 3.92 (s, 1H), 3.80 (s, 3H), 3.71 (s, 3H), 3.65 (s, 3H), 3.24 (ddd, J = 14.1, 10.6, 4.5 Hz, 1H), 2.87 (ddd, J = 16.3, 10.5, 5.9 Hz, 1H), 2.70 (dt, J = 16.2, 4.1 Hz, 1H).
^{13}C NMR (126 MHz, CD_3CN): δ = 157.03, 149.45, 148.71, 141.64, 134.00, 133.62, 129.00, 128.96, 128.44, 128.36, 127.88, 127.80, 127.31, 127.20, 127.03, 112.86, 112.57, 58.40, 56.39, 56.37, 53.25, 39.19, 28.45.
EI HRMS: calculated for $C_{23}H_{23}N_1O_4$ ($[M]^+$): 377.162159, found: 377.162620.
HPLC (IA-3, *n*-heptane/*i*-PrOH 90:10, 298 K, 277 nm): t_R (major) = 20.2 min, t_R (minor) = 22.7 min, er = 96:4 (92% ee).
$[\alpha]_D^{25}$ = −155.0 (*c* = 0.35, $CHCl_3$).

Methyl 1-(benzo[*b*]thiophen-3-yl)-6,7-dimethoxy-3,4-dihydroisoquinoline-2(1H)-carboxylate (18l)

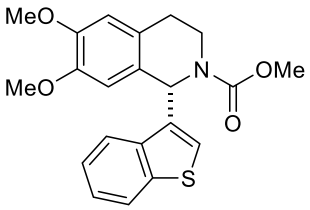

The reaction was performed according to **general procedure a** with benzo[*b*]thiophene-3-carbaldehyde dimethylacetal (**20l**, 21 µL, 0.12 mmol, 1.2 eq.). Purification by silica gel flash column chromatography (DCM/EtOAc 10%) afforded the product as a white foam (31.4 mg, 82 µmol, 82%).
***R*F** (hex/EtOAc 2:1) = 0.39.
^{1}H NMR (501 MHz, CD_3CN): δ = 8.16 (s, 1H), 7.90 (dt, J = 7.9, 1.0 Hz, 1H), 7.43 (ddd, J = 8.1, 7.1, 1.2 Hz, 1H), 7.38 (ddd, J = 8.2, 7.0, 1.3 Hz, 1H), 6.82 (s, 1H), 6.77 (s, 1H), 6.71–6.62 (m, 2H), 3.93 (s, 1H), 3.80 (s, 3H), 3.74 (s, 3H), 3.64 (s, 3H), 3.11–3.00 (m, 1H), 2.92 (ddd, J = 16.4, 12.4, 6.1 Hz, 1H), 2.65 (ddd, J = 16.4, 4.3, 1.5 Hz, 1H).
^{13}C NMR (126 MHz, CD_3CN): δ = 156.91, 149.53, 148.62, 141.38, 139.34, 139.21, 128.25, 128.04, 127.74, 125.65, 125.35, 123.79, 123.56, 112.96, 112.25, 56.35, 53.44, 38.46, 28.03. *Other signals could not be detected due to peak broadening.*
EI HRMS: calculated for $C_{21}H_{21}N_1O_4S_1$ ($[M]^+$): 383.118581, found: 383.119240.
HPLC (IA-3, *n*-heptane/*i*-PrOH 80:20, 298 K, 282 nm): t_R (major) = 8.8 min, t_R (minor) = 11.7 min, er = 92:8 (84% ee).
$[\alpha]_D^{25}$ = −150.0 (*c* = 0.34, $CHCl_3$).

3. Synthesis of Protected *β*-Arylethylamines

4-Methoxyphenyl (3,4-dimethoxyphenethyl)carbamate (11a)

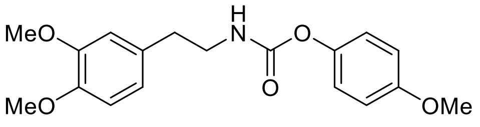

A 100 mL flask equipped with a stir bar was charged with bis(4-methoxyphenyl) carbonate (1.5 g, 5.3 mmol, 1.0 eq.), H_2O (10 mL) and THF (1.3 mL). Homoveratrylamine (**S1**, 0.9 mL, 5.3 mmol, 1.0 eq.) was added dropwise and the mixture was stirred at RT for 16 h. The aqueous layer was extracted with EtOAc (3×50 mL). The combined organic layers were washed with NaOH (1 M) and brine, dried over anhydrous Na_2SO_4, and concentrated. Purification by silica gel flash column chromatography (hex/EtOAc 2:1) yielded the desired product as a white solid (1.6 g, 4.7 mmol, 88%).

$\boldsymbol{R}_\mathbf{F}$ (hex/EtOAc 2:1) = 0.20.

^{1}H NMR (501 MHz, $CDCl_3$): δ = 7.20–7.15 (m, 1H), 7.08 (dd, J = 7.9, 1.7 Hz, 1H), 6.98–6.91 (m, 2H), 6.83 (d, J = 8.0 Hz, 1H), 6.81–6.75 (m, 2H), 5.10 (s, 1H), 3.89 (s, 3H), 3.87 (s, 3H), 3.84 (s, 3H), 3.51 (q, J = 6.7 Hz, 2H), 2.84 (t, J = 7.0 Hz, 2H).

^{13}C NMR (126 MHz, $CDCl_3$): δ = 154.51, 151.84, 149.25, 147.93, 140.09, 131.29, 126.64, 123.41, 120.92, 120.90, 112.55, 112.16, 111.59, 56.11, 56.06, 56.03, 42.77, 35.66.

ESI HRMS: calculated for $C_{18}H_{21}N_1O_5Na_1$ ($[M+Na]^+$): 354.131192, found: 354.131110.

p-Tolyl (3,4-dimethoxyphenethyl)carbamate (11b)

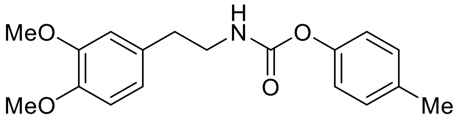

A 100 mL flask equipped with a stir bar was charged with homoveratrylamine (**S1**, 1.5 mL, 8.9 mmol, 1.0 eq.), THF (40 mL), and K_2CO_3 (1.5 g, 11 mmol, 1.2 eq.). 4-methylphenyl chloroformate (1.5 mL, 10.1 mmol, 1.1 eq.) was added and the mixture was stirred at RT for 16 h. The reaction was quenched by addition of HCl (1.2 M) and the aqueous layer was extracted with EtOAc (3×50 mL). The combined organic layers were washed with HCl (1.2 M) and brine, dried over anhydrous Na_2SO_4, and concentrated. Purification by silica gel flash column chromatography (hex/EtOAc 4:1 to 3:1) yielded the desired product as a white solid (1.52 g, 4.82 mmol, 54%).

$\boldsymbol{R}_\mathbf{F}$ (hex/EtOAc 2:1) = 0.31.

^{1}H NMR (501 MHz, CD_2Cl_2): δ = 7.14 (d, J = 8.1 Hz, 2H), 6.98 (d, J = 8.5 Hz, 2H), 6.83 (d, J = 8.1 Hz, 1H), 6.77 (dd, J = 8.1, 2.0 Hz, 1H), 6.75 (d, J = 1.9 Hz, 1H), 5.00 (t, J = 6.0 Hz, 1H), 3.88 (s, 3H), 3.87 (s, 3H), 3.51 (q, J = 6.7 Hz, 2H), 2.83 (t, J = 7.0 Hz, 2H), 2.33 (s, 3H).

^{13}C NMR (126 MHz, CD_2Cl_2): δ = 154.93, 149.26, 148.90, 147.95, 135.04, 131.21, 129.93, 121.41, 120.86, 112.11, 111.60, 56.10, 56.03, 42.60, 35.66, 20.96.

ESI HRMS: calculated for $C_{18}H_{21}N_1O_4Na_1$ ($[M+Na]^+$): 338.136278, found: 338.135940.

Phenyl (3,4-dimethoxyphenethyl)carbamate (11c)[1]

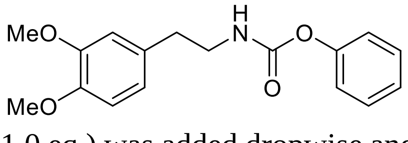

A 100 mL flask equipped with a stir bar was charged with diphenyl carbonate (4.3 g, 20 mmol, 1.0 eq.), H_2O (36 mL) and THF (5 mL). Homoveratrylamine (**S1**, 3.4 mL, 20 mmol, 1.0 eq.) was added dropwise and the mixture was stirred at RT for 16 h. The aqueous layer was extracted with EtOAc (3×50 mL). The combined organic layers were washed with NaOH (2 M) and brine, dried over anhydrous Na_2SO_4, and concentrated. Purification by silica gel flash

column chromatography (hex/EtOAc 2:1 to 1:1) and crystallization from hex/EtOAc yielded the desired product as a white crystalline solid (5.0 g, 17 mmol, 82%).
^{1}H NMR (501 MHz, $CDCl_3$): δ = 7.35 (t, J = 8.0 Hz, 2H), 7.19 (t, J = 6.9 Hz, 1H), 7.10 (d, J = 7.4 Hz, 2H), 6.84 (d, J = 8.1 Hz, 1H), 6.79–6.73 (m, 2H), 5.04 (bs, 1H), 3.89 (s, 3H), 3.88 (s, 3H), 3.51 (q, J = 6.7 Hz, 2H), 2.84 (t, J = 7.0 Hz, 2H).
^{13}C NMR (126 MHz, $CDCl_3$): δ = 154.70, 151.15, 149.28, 147.97, 131.18, 129.42, 125.43, 121.69, 120.86, 112.12, 111.62, 56.10, 56.04, 42.61, 35.65.
EI HRMS: calculated for $C_{17}H_{19}N_1O_4$ ($[M]^+$): 301.130859, found: 301.130390.

4-Fluorophenyl (3,4-dimethoxyphenethyl)carbamate (11d)

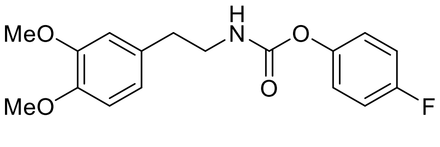

A 100 mL flask equipped with a stir bar was charged with homoveratrylamine (**S1**, 0.9 mL, 5.3 mmol, 1.0 eq.), THF (25 mL), and K_2CO_3 (0.88 g, 6.4 mmol, 1.2 eq.). 4-fluorophenyl chloroformate (0.77 mL, 5.9 mmol, 1.1 eq.) was added and the mixture was stirred at RT for 1.5 h. The reaction was quenched by addition of HCl (1.2 M) and the aqueous layer was extracted with EtOAc (3×50 mL). The combined organic layers were washed with HCl (1.2 M) and brine, dried over anhydrous Na_2SO_4, and concentrated. Purification by silica gel flash column chromatography (hex/EtOAc 3:1 to 2:1) yielded the desired product as a white solid (0.84 g, 2.6 mmol, 49%).
***R*F** (hex/EtOAc 2:1) = 0.38.
^{1}H NMR (501 MHz, CD_2Cl_2): δ = 7.11–7.00 (m, 4H), 6.83 (d, J = 7.9 Hz, 1H), 6.80–6.73 (m, 2H), 5.11 (s, 1H), 3.83 (s, 3H), 3.81 (s, 3H), 3.47 (q, J = 6.7 Hz, 2H), 2.81 (t, J = 7.0 Hz, 2H).
^{13}C NMR (126 MHz, CD_2Cl_2): δ = 154.76, 149.72, 147.55 (d, *J* = 2.7 Hz), 131.58, 123.49 (d, *J* = 8.5 Hz), 121.11, 116.09 (d, *J* = 23.4 Hz), 112.44 (d, *J* = 72.2 Hz), 56.25, 56.18, 42.88, 35.80.
^{19}F NMR (471 MHz, CD_2Cl_2): δ = -118.67.
ESI-HRMS: calculated for $C_{17}H_{18}N_1O_4F_1Na_1$ ($[M+Na]^+$): 342.111206, found: 342.111060.

4-Chlorophenyl (3,4-dimethoxyphenethyl)carbamate (11e)

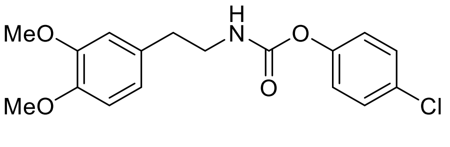

A 100 mL flask equipped with a stir bar was charged with homoveratrylamine (**S1**, 1.5 mL, 8.9 mmol, 1.0 eq.), THF (40 mL), and K_2CO_3 (1.5 g, 11 mmol, 1.2 eq.). 4-chlorophenyl chloroformate (1.4 mL, 9.8 mmol, 1.1 eq.) was added and the mixture was stirred at RT for 16 h. The reaction was quenched by addition of HCl (1.2 M) and the aqueous layer was extracted with EtOAc (3×50 mL). The combined organic layers were washed with HCl (1.2 M) and brine, dried over anhydrous Na_2SO_4, and concentrated. Purification by silica gel flash column chromatography (hex/EtOAc 4:1 to 3:1) yielded the desired product as a white solid (1.56 g, 4.67 mmol, 52%).
***R*F** (hex/EtOAc 2:1) = 0.35.
^{1}H NMR (501 MHz, CD_2Cl_2): δ = δ 7.36–7.29 (m, 2H), 7.08–7.03 (m, 2H), 6.83 (d, J = 7.9 Hz, 1H), 6.80–6.73 (m, 2H), 5.12 (s, 1H), 3.83 (s, 3H), 3.81 (s, 3H), 3.48 (q, J = 6.7 Hz, 2H), 2.81 (t, J = 7.0 Hz, 2H).
^{13}C NMR (126 MHz, CD_2Cl_2): δ = 154.43, 150.21, 149.73, 148.43, 131.53, 130.72, 129.58, 123.44, 121.11, 112.72, 112.15, 56.25, 56.18, 42.88, 35.78.
ESI HRMS: calculated for $C_{17}H_{18}Cl_1N_1O_4Na_1$ ($[M+Na]^+$): 358.081656, found: 358.081410.

4-Nitrophenyl (3,4-dimethoxyphenethyl)carbamate (11f)

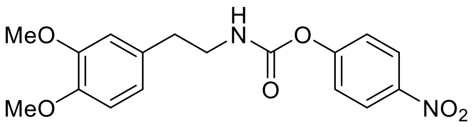

A 100 mL flask equipped with a stir bar was charged with bis(4-nitrophenyl) carbonate (1.6 g, 5.3 mmol, 1.0 eq.), H_2O (10 mL) and THF (1.3 mL). Homoveratrylamine (**S1**, 0.9 mL, 5.3 mmol, 1.0 eq.) was added dropwise and the mixture was stirred at RT for 16 h. The aqueous layer was extracted with EtOAc (3×50 mL). The combined organic layers were washed with brine, dried over anhydrous Na_2SO_4, and concentrated. Purification by silica gel flash column chromatography (hex/EtOAc 3:1 to 2:1) yielded the desired product as a white solid (1.0 g, 2.9 mmol, 54%).

***R*F** (hex/EtOAc 2:1) = 0.20.

^{1}H NMR (501 MHz, $CDCl_3$): δ = 8.27–8.20 (m, 2H), 7.32–7.27 (m, 2H), 6.84 (d, J = 8.1 Hz, 1H), 6.77 (dd, J = 8.1, 2.0 Hz, 1H), 6.74 (d, J = 2.0 Hz, 1H), 5.14 (t, J = 6.3 Hz, 1H), 3.89 (s, 3H), 3.88 (s, 3H), 3.54 (q, J = 6.7 Hz, 2H), 2.85 (t, J = 7.0 Hz, 2H).

^{13}C NMR (126 MHz, $CDCl_3$): δ = 156.03, 153.17, 149.34, 148.09, 144.89, 130.74, 125.25, 122.04, 120.86, 112.06, 111.63, 56.10, 56.06, 42.64, 35.49.

ESI HRMS: calculated for $C_{17}H_{18}N_2O_6Na_1$ ($[M+Na]^+$): 369.105706, found: 369.105520.

Methyl (2-bromo-4,5-dimethoxyphenethyl)carbamate (S2)

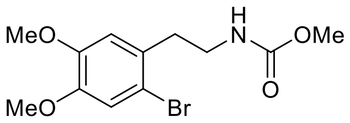

A 50 mL round-bottom flask equipped with a stir bar was charged with carbamate **7** (1.0 g, 4.2 mmol, 1.0 eq.) and AcOH (15 mL). Bromine was added and the reaction was stirred at RT for 90 min. The mixture was poured onto ice water and the aqueous layer was extracted with EtOAc (3×). The combined organic layers were washed with sat. aq. $NaHCO_3$, H_2O, sat. aq. Na_2SO_3, and brine, dried over anhydrous Na_2SO_4, and concentrated. Purification by silica gel flash column chromatography (hex/EtOAc 60:40) yielded the desired product as a white solid (1.2 g, 3.9 mmol, 92%).

***R*F** (hex/EtOAc 1:1) = 0.41.

^{1}H NMR (501 MHz, $CDCl_3$): δ = 7.00 (s, 1H), 6.72 (s, 1H), 4.74 (s, 1H), 3.85 (s, 6H), 3.67 (s, 3H), 3.42 (q, J = 6.8 Hz, 2H), 2.88 (d, J = 7.2 Hz, 2H).

^{13}C NMR (126 MHz, $CDCl_3$): δ = 157.19, 148.63, 148.48, 130.16, 115.80, 114.40, 113.58, 56.32, 56.24, 52.23, 41.10, 36.09.

EI HRMS: calculated for $C_{12}H_{16}N_1O_4Br_1$ ($[M]^+$): 317.025734, found: 317.025210.

Methyl (dimethoxyphenethyl)carbamate (7-*D*)

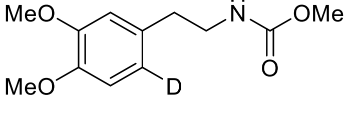

A flame-dried 50 mL Schlenk under argon was charged with carbamate **S2** (499 mg, 1.6 mmol, 1.0 eq.) and THF (16 mL). The mixture was cooled to 0 °C, NaH (60 wt%, 125 mg, 31 mmol, 2.0 eq.) was added in one portion, and the reaction was stirred for 45 min. The mixture was cooled to −78 °C, *n*-BuLi (2.5 m in hexane, 1.3 mL, 3.3 mmol, 2.1 eq.) was added carefully, and the reaction was stirred for 2 h at −78 °C. CD_3OD (0.65 mL, 16 mmol, 10 eq.) was added carefully and the mixture was stirred for another 15 min, before warming to RT. Sat. Aq. NH_4Cl was added, and the aqueous layer was extracted with EtOAc (3×). The combined organic layers were washed with sat. aq. $NaHCO_3$ and brine, dried over anhydrous Na_2SO_4, and concentrated. Purification by silica gel flash column chromatography (hex/EtOAc 2:1 to 3:2) yielded the desired product as a white solid (298 mg, 1.2 mmol, 79%,

72% deuterium incorporation by ^{1}H NMR spectroscopy). *No further NMR spectroscopic data is provided, due to incomplete deuteration.*
EI HRMS: calculated for $C_{12}H_{16}N_1O_4D_1$ ($[M]^+$): 240.121486, found: 240.121450.

4. Synthesis of Aldehydes and Acetals

Aromatic dimethylacetals **20a-l** were purchased from commercial suppliers and distilled prior to use, or prepared from the corresponding aldehydes according to literature procedures.

Hexanal-2,2-D_2 (14-D_2)

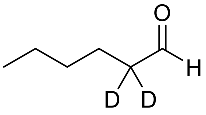

A 5 mL microwave vial was charged with hexanal (**14**, 1.0 mL, 8.1 mmol, 1.0 eq.), deuterium oxide (2.0 mL, 110 mmol, 13.6 eq.), and 4-DMAP (13 mg, 0.11 mmol, 1 mol%). The vial was capped, and the mixture was heated to 100 °C for 24 h. After cooling to RT, the reaction was diluted with Et_2O, and washed with HCl (1.2 M), saturated aqueous $NaHCO_3$, and brine. The organic layers were dried over Na_2SO_4 and concentrated to give the desired product, which was used without further purification (370 mg, 3.6 mmol, 44%, >90% *d*).
^{1}H NMR (501 MHz, $CDCl_3$): δ = 9.76 (s, 1H), 1.61 (t, J = 7.1 Hz, 2H), 1.39–1.24 (m, 4H), 0.98–0.83 (m, 3H).
^{13}C NMR (126 MHz, $CDCl_3$): δ = 203.27, 43.26 (p, *J* = 19.4 Hz), 31.39, 22.54, 21.78, 13.98.
EI HRMS: calculated for $C_6H_{10}D_2O_1$ ($[M]^+$): 102.100819, found: 102.100840.

5. Synthesis of IDPi Catalysts

(*S*,*S*)-Ph-IDPi with $SO_2C_6F_5$ core (1b)

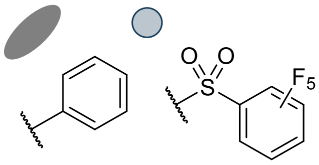

The compound was synthesized following a previously published procedure[1] and gave the product as an off-white powder (214 mg, 0.207 mmol, 72%).
^{1}H NMR (600 MHz, CD_2Cl_2) δ = 8.13–8.06 (m, 4H), 8.03 (d, J = 8.3 Hz, 2H), 7.76 (ddd, J = 8.1, 6.7, 1.3 Hz, 2H), 7.58 (ddd, J = 8.1, 6.3, 1.6 Hz, 2H), 7.56–7.52 (m, 2H), 7.50 (d, J = 8.4 Hz, 2H), 7.43 (bs, 2H), 7.39–7.32 (m, 8H), 7.32–7.22 (m, 6H), 7.08 (t, J = 7.4 Hz, 2H), 6.87 (t, J = 7.4 Hz, 4H), 6.57 (d, J = 7.5 Hz, 4H), 5.62 (bs, 1H, OH). *The OH signal integration was >1 likely due to residual water in the sample.
^{19}F NMR (565 MHz, CD_2Cl_2) δ = -136.26 (d, J = 19.8 Hz, 4F), -147.19 (bs, 2H), -160.04 (t, J = 20.4 Hz, 4F).
^{31}P NMR (243 MHz, CD_2Cl_2) δ = -15.0.
^{13}C NMR (151 MHz, CD_2Cl_2) δ = 144.4 (dm, J = 259.6 Hz), 143.9 (t, J = 5.1 Hz), 143.8 (dm, J = 261.5 Hz), 143.4 (t, J = 5.3 Hz), 137.7 (dm, J = 253.8 Hz), 135.9, 135.9, 133.9, 133.9 (t, J = 1.9 Hz), 132.4, 132.4, 132.2, 132.0, 131.7, 131.4, 129.9, 129.7, 129.1, 128.8, 128.5, 128.1, 128.0, 127.9, 127.7, 127.3, 127.2, 127.2, 127.1, 127.0, 123.7 (t, J = 1.4 Hz), 122.6, 117.9 (m).
ESI HRMS: calculated for $C_{76}H_{40}N_3O_8F_{10}S_2P_2^-$ ($[M-H]^-$): 1438.158170, found: 1438.157800.
$[\alpha]_D^{25}$ = +20.8 (*c* = 0.605 mg/mL, $CHCl_3$).

6. Acidity Measurements

The pK_a measurements rely on the UV-Vis spectrophotometric or NMR spectroscopic determination of the difference between a reference acid with a previously investigated pK_a value and an acid catalyst with an unknown pK_a value. The experimental setup and methodology for the determination of pK_a values in acetonitrile (MeCN) and 1,2-dichloroethane (DCE) are based on the methods developed at the University of Tartu in which a mixture of two acids is titrated with a basic and an acidic titrant to acquire spectra of both the fully protonated and the fully deprotonated forms as well as the partly ionized species of the acids. Each of the acids was also titrated on its own to determine the UV-Vis spectra and the NMR shifts of their neutral and anionic forms. The reference acid was chosen to preferably not differ much more than 1 pK_a unit from the expected pK_a value of the investigated compound. Both the UV-Vis spectrophotometric determination[2–6] and the NMR spectroscopic method[7,8] were published earlier in detail.

In the case of the UV-Vis spectrophotometric method, the spectral data of the titration of the mixture of the two acids were mathematically treated by multilinear regression analysis at multiple wavelengths to determine the dissociation levels of both acids at different steps of the titration. The differences of pK_a values (ΔpK_a) of the acid catalysts and the used reference acids were calculated from the obtained dissociation levels, expressed as $\alpha_1 = [A_1^-]/([A_1^-] + [HA_1])$ in the case of the acid catalysts and $\alpha_2 = [A_2^-]/([A_2^-] + [HA_2])$ in the case of the reference acids. Based on the α values, the difference of the pK_a values can be calculated using equation (1).

$$\Delta \mathrm{p}K_\mathrm{a} = log\frac{\alpha_1(1-\alpha_2)}{(1-\alpha_1)\alpha_2} \qquad \text{eq. (1)}$$

The NMR spectroscopic method was used for the independent confirmation of the UV-Vis data and thus only one measurement with one reference acid was carried out. The ionization of **3a** and the reference acid was monitored by means of ^{19}F NMR spectroscopy using the averaged chemical shifts of the signals of the exchanging forms of the compounds from the spectra of the solutions obtained during the titration. The α values were calculated using the shifts of the fully protonated and fully deprotonated forms as well as the shifts corresponding to the equilibrium mixtures of protonated and deprotonated forms (eq. (2)) and the pK_a values were then calculated using equation (1).

$$\alpha = \frac{(\delta_n - \delta_0)}{(\delta_1 - \delta_0)} \qquad \text{eq. (2)}$$

Experimental setup

All solutions were set up in a GS Megaline Glovebox, type MEGA 3, filled with 99.999% pure argon to ensure an oxygen and moisture content inside the glovebox below 10 ppm. The utilized reference acids are all known compounds, for pK_a values in MeCN see[3], for pK_a values in DCE see[4]. As solvents acetonitrile (Romil 190 SpS far UV/gradient quality) and 1,2-dichloroethane (Thermoscientific, 99+%, for spectroscopy) were used after drying over molecular sieves (3 Å) for at least 24 h (usually longer) to ensure a water content below 6 ppm. The water content was determined after drying by means of coulometric Karl-Fischer titration.

The acidic titrant solution was prepared using trifluoromethanesulfonic acid (Aldrich, ≥99%), for the basic titrant solution 1-*tert*-butyl-2,2,2-tri(1-pyrrolidinyl)-phosphazene (Aldrich, ≥97%) was used. The concentrations of the solutions used for the titrations are listed in Table S3.

Table S3 Concentrations of titrant and acid mixture solutions for titration (mol L^{-1}).

**Method**	**Acidic titrant**	**Basic titrant**	**Mixture of studied acids**
UV-Vis	4.6 – 6.6×10^{-3}	2.3 – 3.3×10^{-3}	4.5×10^{-6} – 1.1×10^{-4}
NMR	5.4 – 6.2×10^{-2}	5.4 – 6.0×10^{-2}	4.0 – 5.0×10^{-4}

The UV-Vis spectrophotometric titrations were carried out using an Agilent Cary 60 spectrophotometer connected with two optical fibre cables to an external cell compartment in the glovebox and a quartz cuvette with 10 mm optical path length. For the NMR spectroscopic titrations, a Bruker Avance Neo 600 MHz NMR spectrometer (^{1}H resonance frequency 600 MHz, ^{19}F resonance frequency 565 MHz, ^{31}P resonance frequency 243 MHz) equipped with a cryogenic broadband probe (BBO cryoProbe) was used with an external standard for referencing containing 1,4-difluorobenzene and triphenylphosphine oxide in deuterated benzene, sealed in a capillary tube. The titration was performed in non-deuterated solvent and the external capillary was used for locking and shimming. After preparing the titrant solutions in the glovebox, they were transferred into Schlenk tubes with rubber stoppers for titration. The NMR tube was used with a septum. All spectra were recorded at 298 K and processed with MestReNova 15.0.0 suits of programs.

Summary of individual pK_a measurements

Table S4 Data of individual acidity measurements of the acid catalysts **1b**, **2a**, **2b**, **3a**, and **3b**, and their assigned pK_a values[a].

**Catalyst**	**Solvent**	**Reference acid**	**pK_a ref.**	**ΔpK_a**	**pK_a cat.**	**±**	**Assigned pK_a**
**1b**	MeCN	4-NO_2-C_6H_4-SO_2-$CH(CN)_2$	6.01	-0.63	6.64	0.15	6.9±0.4
	MeCN	3-NO_2-C_6H_4-SO_2-$CH(CN)_2$	6.16	-0.69	6.82	0.10	
	MeCN	Me-TCNPD	7.36	0.21	7.13	0.10	
**2a**	DCE	Ph-TCNP	-9.4	-1.65	-7.75	0.10	-7.8±0.2
	DCE	Me-TCNP	-8.6	-1.27	-7.83	0.10	
	DCE	3,4-$(OMe)_2$-C_6H_3-TCNP	-8.7	-0.94	-7.76	0.05	
**2b**	MeCN	2,4,6-$(SO_2CF_3)_3$-phenol	4.48	0.35	4.13	0.05	4.1±0.1
	MeCN	2,4,6-$(SO_2F)_3$-phenol	5.22	1.14	4.07	0.05	
**3a**	DCE	Me-TCNP	-8.6	-1.27	-7.33[b]	0.10	-7.4±0.2
	DCE	Me-TCNP	-8.6	-1.28	-7.32[b]	0.15	
	DCE	Me-TCNP	-8.6	-1.35	-7.25[b]	0.10	
	DCE	4-NO_2-C_6H_4-SO_2-NH-SO_2CF_3	-7.8	-0.34	-7.46[c]	0.10	
**3b**	MeCN	2,4,6-$(SO_2F)_3$-phenol	5.22	0.52	4.70	0.10	4.7±0.1
	MeCN	2,4,6-$(SO_2CF_3)_3$-phenol	4.48	-0.18	4.66	0.05	

[a]UV-Vis measurement results if not stated otherwise. The pK_a values presented for DCE are relative ion-pair acidities (pK_{ip}) against picric acid as reference. They are almost indistinguishable from relative pK_a values, so we use the notion of pK_a. [b]Measurement of three different batches of catalyst **3a**. [c]NMR measurement result.

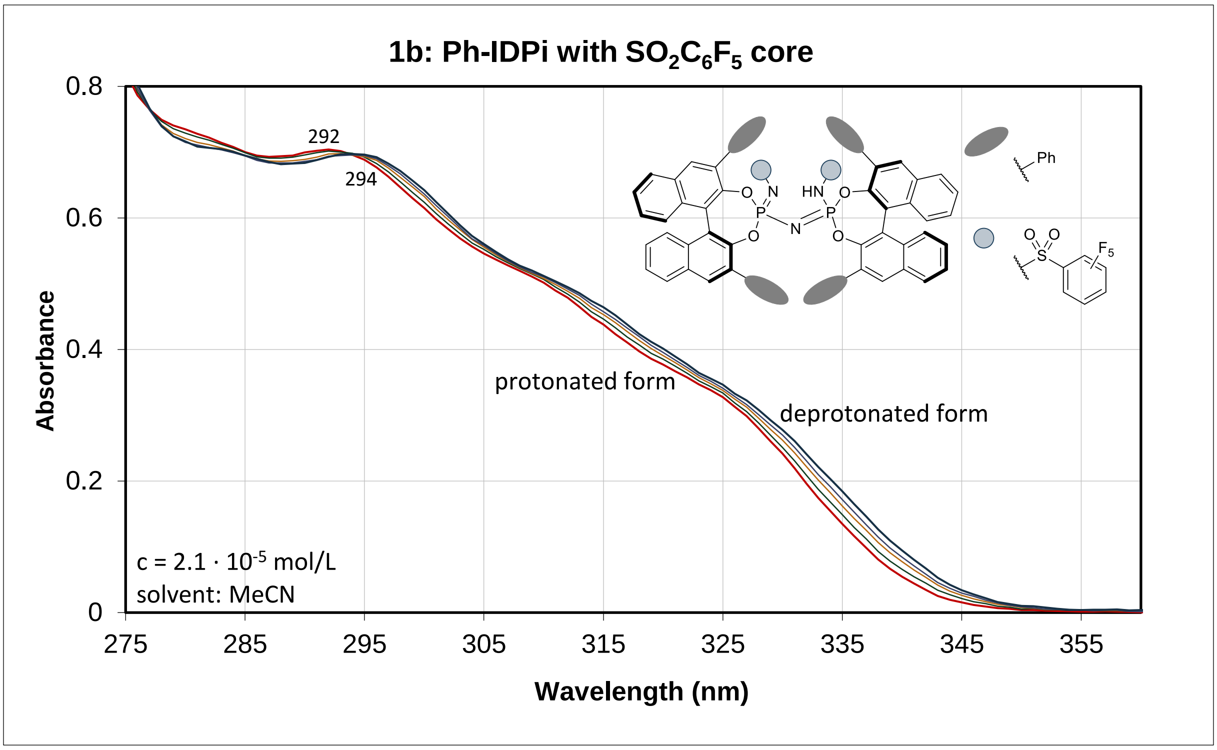

Figure S2 UV-Vis spectrophotometric titration spectra of **1b**.

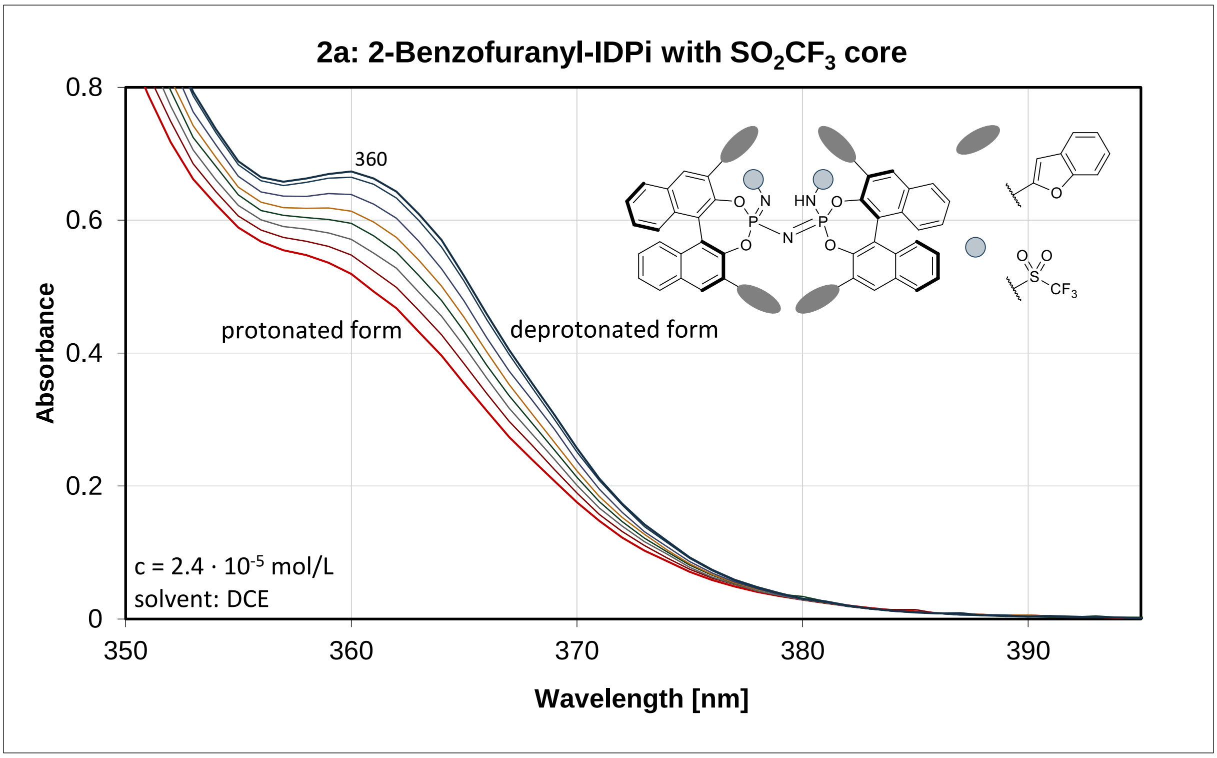

Figure S3 UV-Vis spectrophotometric titration spectra of **2a**.

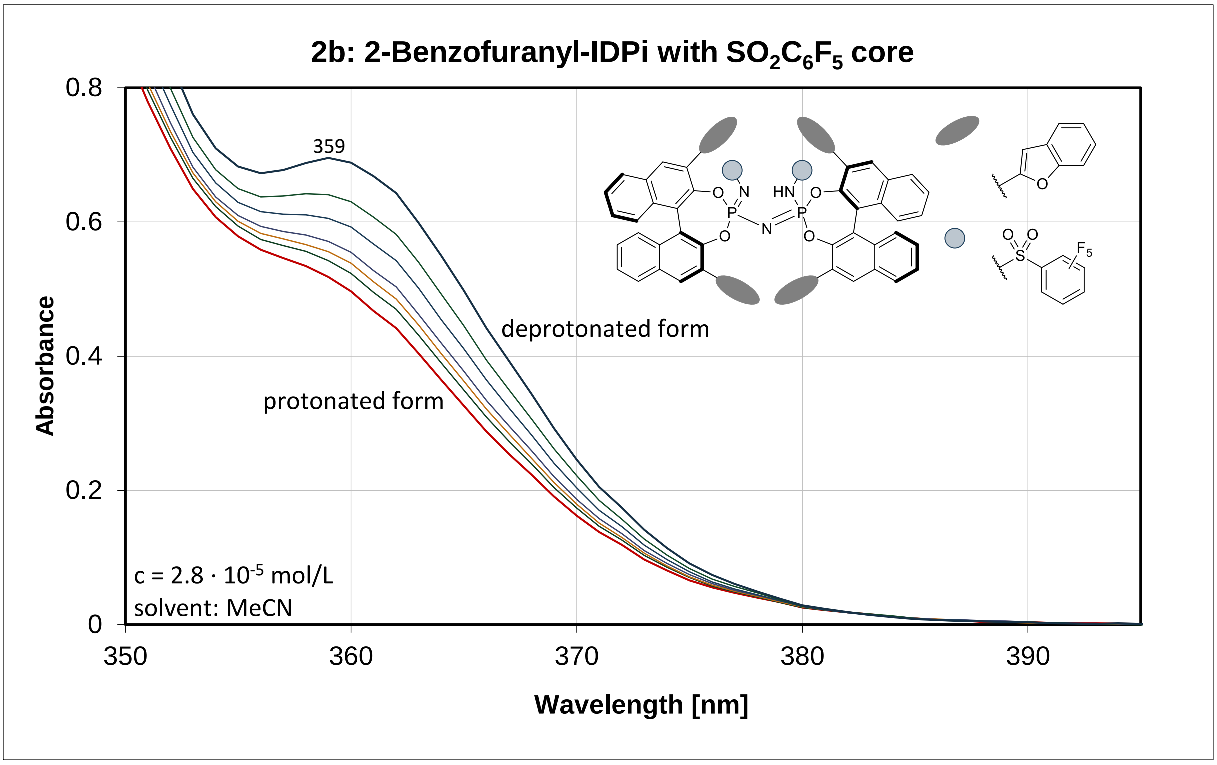

Figure S4 UV-Vis spectrophotometric titration spectra of **2b**.

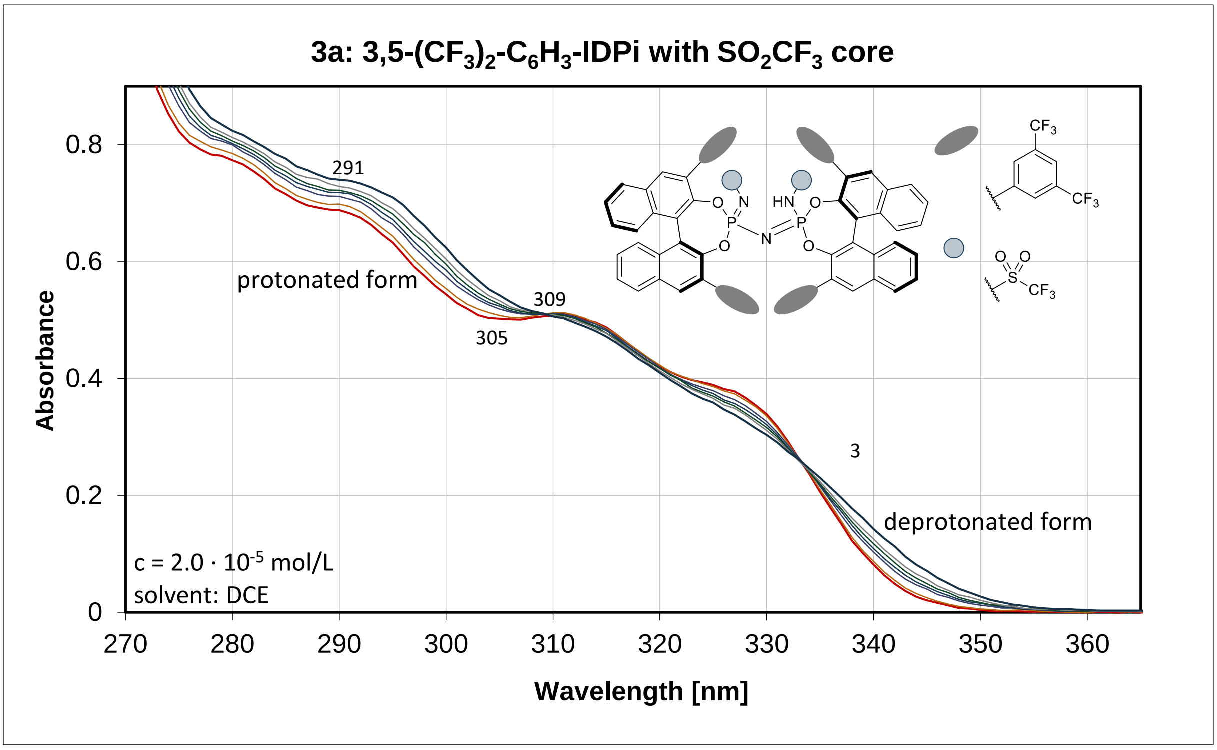

Figure S5 UV-Vis spectrophotometric titration spectra of **3a**.

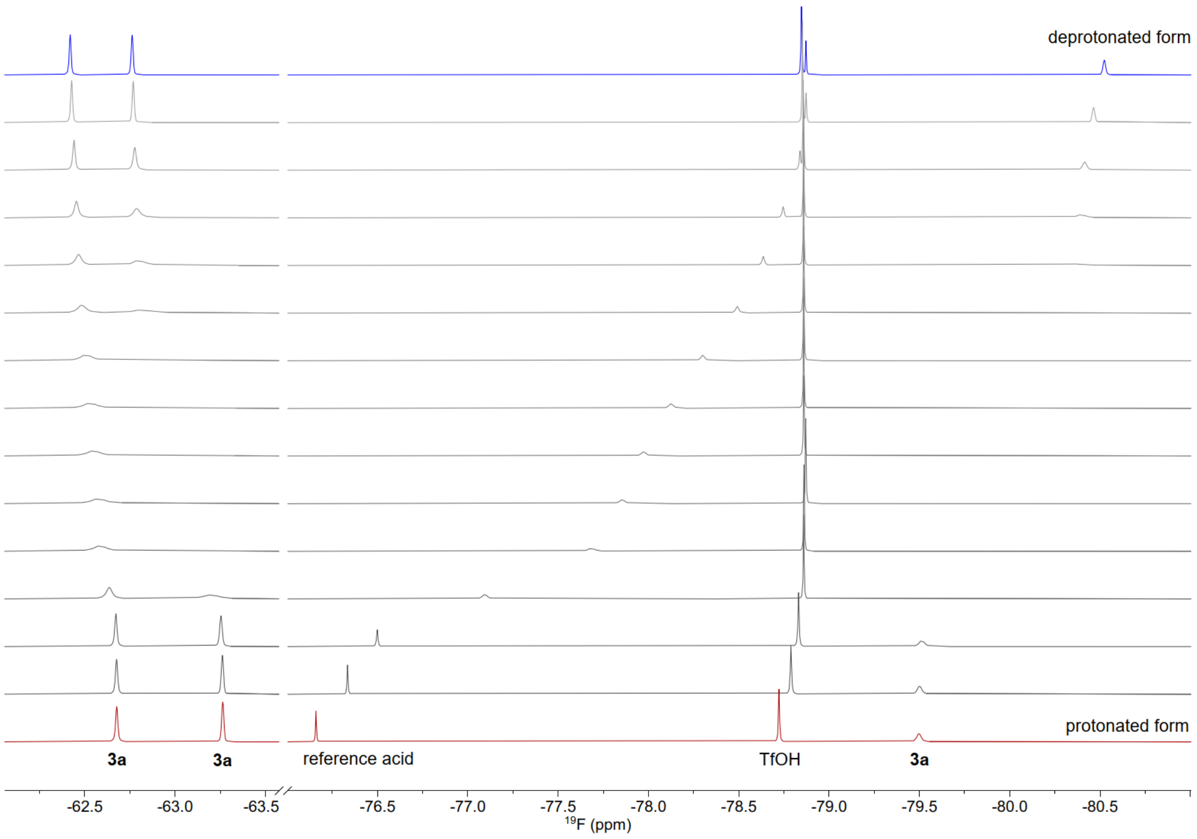

Figure S6 ^{19}F NMR spectroscopic titration spectra of **3a** against 4-NO_2-C_6H_4-SO_2-NH-SO_2CF_3 in DCE (c = 8.0×10^{-4} mol/L).

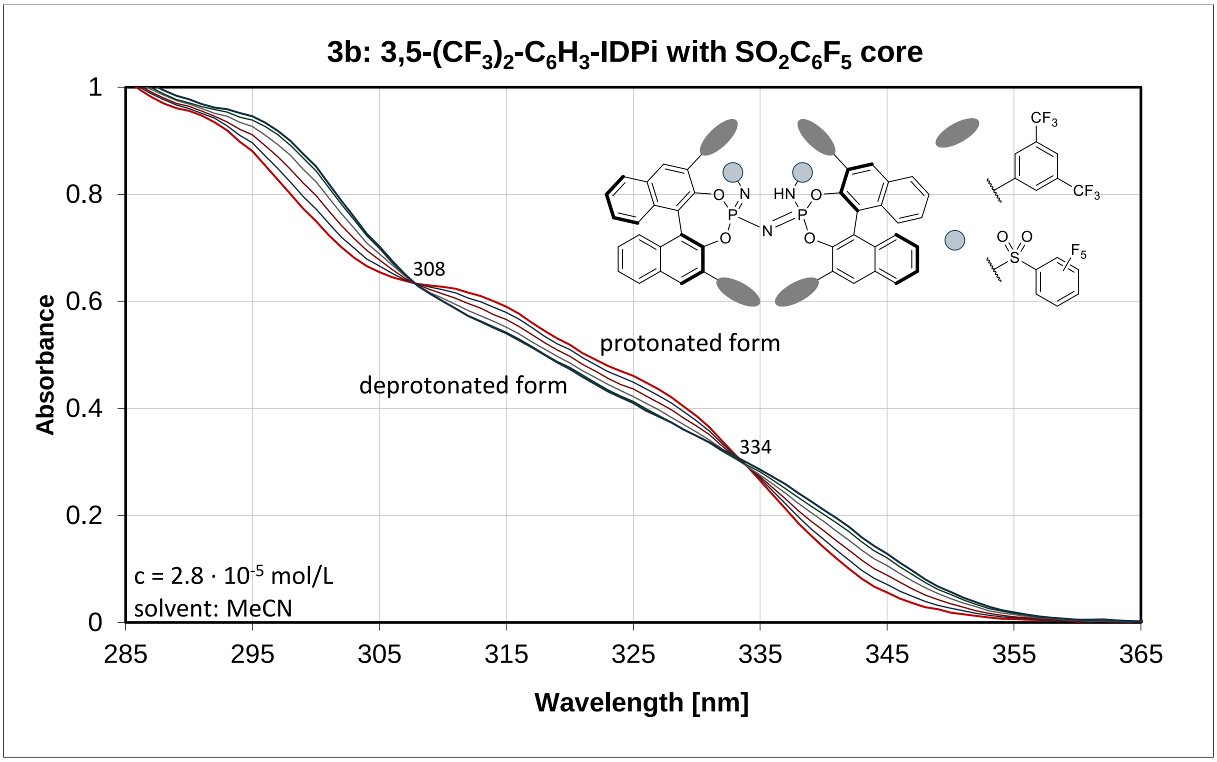

Figure S7 UV-Vis spectrophotometric titration spectra of **3b**.

Competition experiment between catalysts 2b and 3b

To confirm the acidifying effect of the benzofuranyl group a titration experiment measuring the pK_a of catalyst **2b** directly against catalyst **3b** in acetonitrile was carried out using the NMR spectroscopic method as described above. The ionization of **2b** was monitored by means of ^{1}H NMR spectroscopy (Figure S8), the ionization of **3b** by ^{19}F NMR spectroscopy (Figure S9). To enhance the signal-to-noise ratio for the ^{1}H NMR measurements the signal of the solvent (CH_3CN) was suppressed using a WET sequence (bruker pulse sequence: wet).[9]

The experiment clearly confirms catalyst **2b** as the more acidic catalyst compared to catalyst **3b** with a difference between the two catalysts of ΔpK_a = 0.56 (with within-series standard deviation of 0.01), which matches the difference between the independently determined pK_a values of the two catalysts (see Table S4).

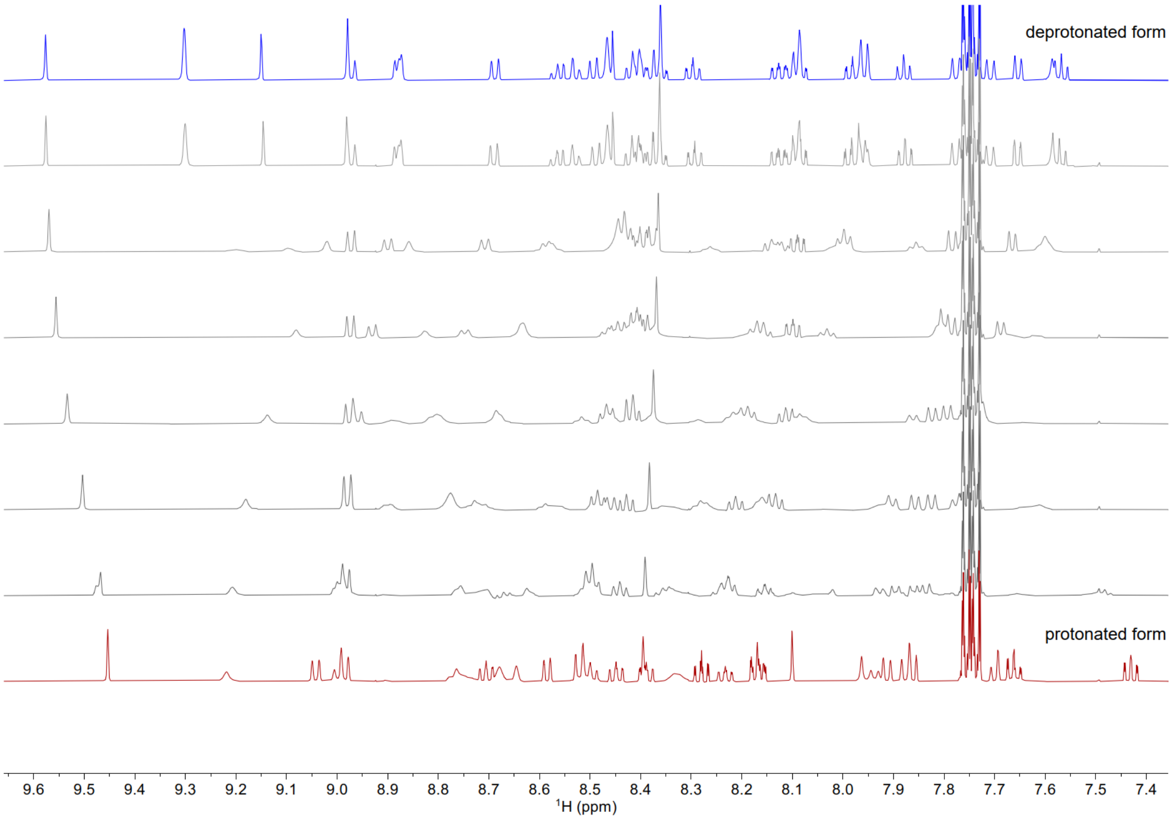

Figure S8 ^{1}H NMR spectroscopic titration spectra of **2b** against **3b** in MeCN (c = 4.0×10^{-4} mol/L).

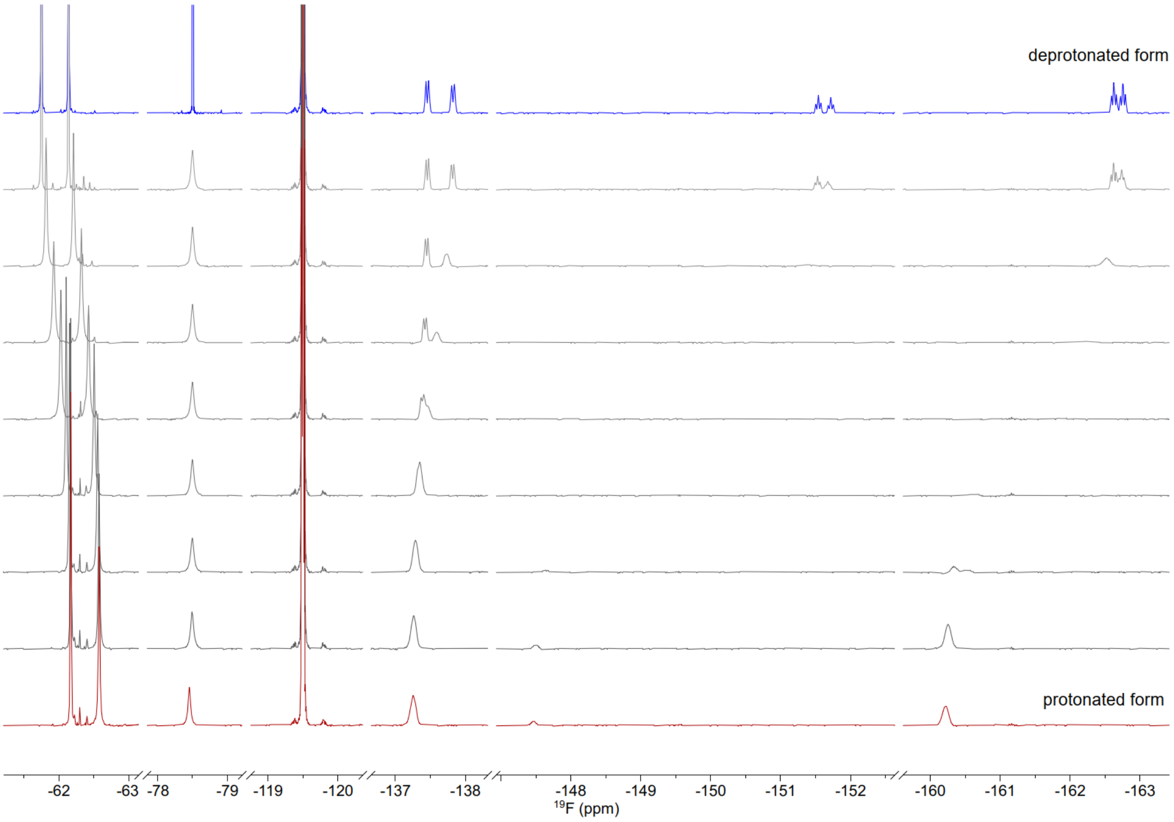

Figure S9 ^{19}F NMR spectroscopic titration spectra of **2b** against **3b** in MeCN (c = 4.0×10^{-4} mol/L).

7. NMR Kinetics

The raw data, as extracted from the processed NMR files, is attached as tables in individual tabstop-separated txt files. The data tables also include the calculated values for the corresponding plots. The files are named as follows:

**Hammett Plots**	**Data file**	**IDPi**		**R**
	H2a-A	**2a**		NO_2
	H2a-B	**2a**		Cl
	H2a-C	**2a**		F
	H2a-D	**2a**		H
	H2a-E	**2a**		Me
	H2a-F	**2a**		OMe
	H3a-A	**3a**		NO_2
	H3a-B	**3a**		Cl
	H3a-C	**3a**		F
	H3a-D	**3a**		H
	H3a-E	**3a**		Me
	H3a-F	**3a**		OMe
**KIE Experiments**	**Data file**	**IDPi**		**substrate**
	KIE-A	**2b**		hexanal
	KIE-B	**2b**		**16a**
**Reaction Order**	**Data file**	**c_0 (7) / M**	**c_0 (14) / M**	**cat.-loading**
	O2b-A	0.05	0.13	1 mol%
	O2b-B	0.10	0.13	1 mol%
	O2b-C	0.20	0.13	1 mol%
	O2b-D	0.10	0.063	1 mol%
	O2b-E	0.10	0.26	1 mol%
	O2b-F	0.10	0.13	0.5 mol%
	O2b-G	0.10	0.13	1.5 mol%
**Eyring Plots**	**Data file**	**IDPi**		**T / °C**
	E2b-A	**2b**		10
	E2b-B (= O2b-B)	**2b**		25
	E2b-C	**2b**		35
	E2b-D	**2b**		45
	E2b-E	**2b**		60
	E3a-A	**3a**		10
	E3a-B	**3a**		25
	E3a-C	**3a**		35
	E3a-D	**3a**		45
	E3a-E	**3a**		60

Hammett Plots

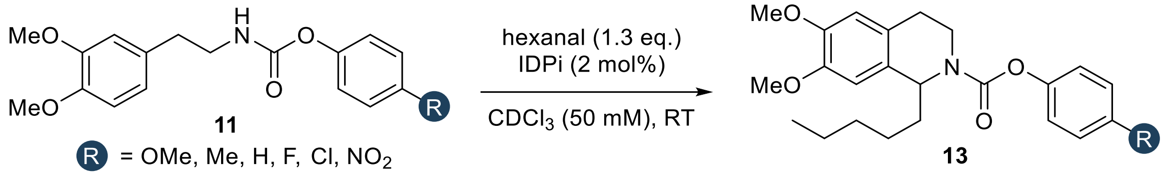

Individual rate experiments were conducted on a 0.025 mmol scale. Stock solutions of carbamate substrates **11** (0.0375 mmol in 0.45 mL $CDCl_3$) and IDPi catalyst **2a** and **3a** (14 mol% in 1.4 mL $CDCl_3$) were prepared. Individual oven-dried NMR tubes were charged with substrate **11** (0.3 mL, 0.025 mmol, 1.0 eq.) and IDPi catalyst (0.2 mL, 2 mol%). Triphenylmethane (1.0 M in PhMe, 25 µL, 1.0 eq.) was added as internal standard to each mixture, and the reaction solution was homogenized by shaking. Hexanal (4.0 µL, 0.0325 mmol, 1.3 eq.) was added, and product formation was followed by ^{1}H NMR spectroscopy approximately every 30 min. Initial rates for each reaction were extracted by linearization of the product concentration profile at <30% conversion using the LINEST function in Microsoft excel.

IDPi 2a

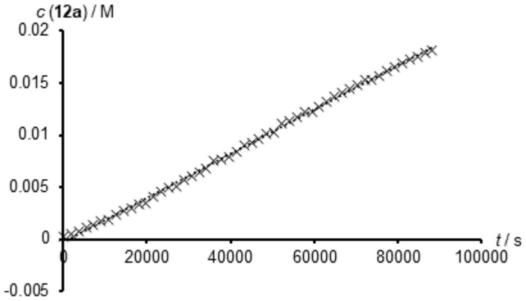

(H2a-A), IDPi **2a**, R = NO_2, σ_p = 0.78			
LINEST		SYNTAX (Y=mX + b)	
2.11758E-07	-0.000256733	m	b
1.16773E-09	5.98079E-05	error m	error b
0.998542482	0.00021451	r^2	error Y

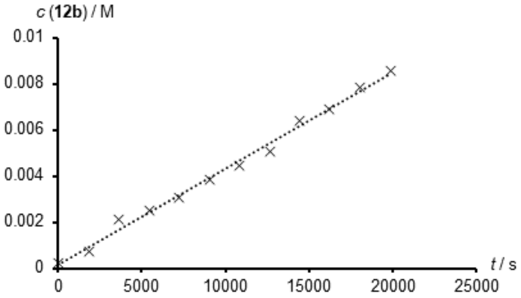

(H2a-B), IDPi **2a**, R = Cl, σ_p = 0.23			
LINEST		SYNTAX (Y=mX + b)	
4.17523E-07	0.000157456	m	b
1.10118E-08	0.000129238	error m	error b
0.993092053	0.000237503	r^2	error Y

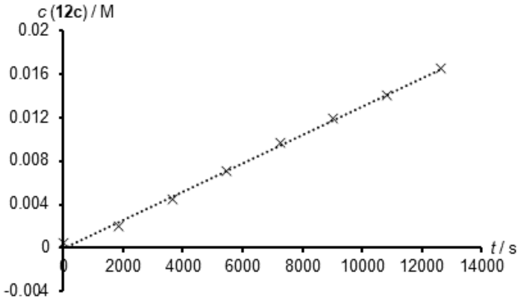

(H2a-C), IDPi **2a**, R = F, σ_p = 0.06			
LINEST		SYNTAX (Y=mX + b)	
1.30874E-06	-1.86435E-05	m	b
2.48091E-08	0.000187833	error m	error b
0.997848545	0.000290141	r^2	error Y

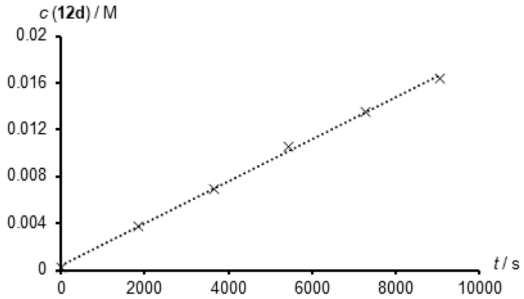

(H2a-D), IDPi **2a**, R = H, σ_p = 0.00			
LINEST		SYNTAX (Y=mX + b)	
1.79692E-06	0.000404118	m	b
3.11563E-08	0.000171281	error m	error b
0.998798925	0.000235771	r^2	error Y

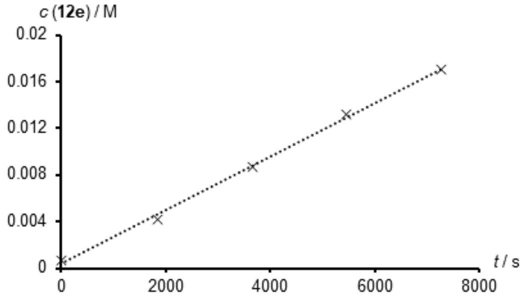

(H2a-E), IDPi **2a**, R = Me, σ_p = -0.17			
LINEST		SYNTAX (Y=mX + b)	
2.29819E-06	0.00035523	m	b
5.95521E-08	0.000265792	error m	error b
0.997989656	0.000341922	r^2	error Y

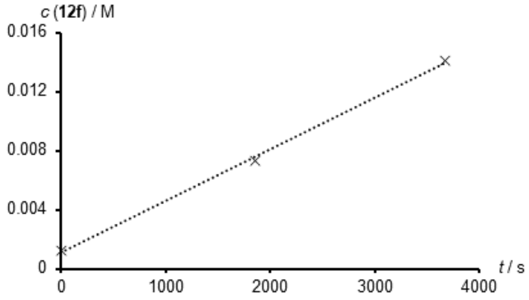

(H2a-D), IDPi **2a**, R = OMe, σ_p = -0.27			
LINEST		SYNTAX (Y=mX + b)	
3.50081E-06	0.001070173	m	b
1.53731E-07	0.000365699	error m	error b
0.998075363	0.000399501	r^2	error Y

IDPi 3a

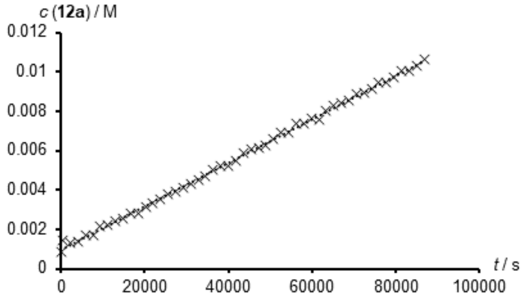

(H3a-A), IDPi **3a**, R = NO_2, σ_p = 0.78			
LINEST		SYNTAX (Y=mX + b)	
1.10451E-07	0.000960831	m	b
5.9009E-10	2.9507E-05	error m	error b
0.998631826	0.00010821	r^2	error Y

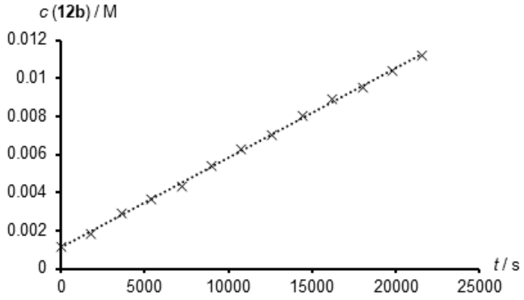

(H3a-B), IDPi **3a**, R = Cl, σ_p = 0.23			
LINEST		SYNTAX (Y=mX + b)	
4.71918E-07	0.001102661	m	b
4.59652E-09	5.85319E-05	error m	error b
0.998957524	0.000111638	r^2	error Y

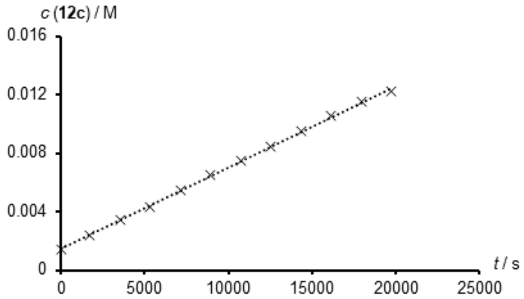

(H3a-C), IDPi **3a**, R = F, σ_p = 0.06			
LINEST		SYNTAX (Y=mX + b)	
5.57321E-07	0.001429282	m	b
4.84864E-09	5.64663E-05	error m	error b
0.999243689	0.000104327	r^2	error Y

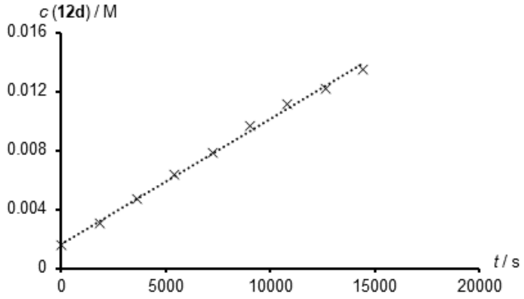

(H3a-D), IDPi **3a**, R = H, σ_p = 0.00			
LINEST		SYNTAX (Y=mX + b)	
8.45255E-07	0.00164679	m	b
1.67588E-08	0.000144226	error m	error b
0.997255796	0.000233968	r^2	error Y

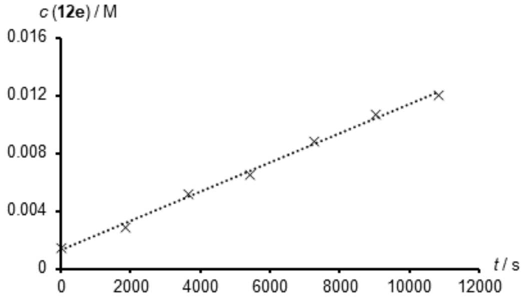

(H3a-E), IDPi **3a**, R = Me, σ_p = -0.17			
LINEST		SYNTAX (Y=mX + b)	
1.00993E-06	0.001287291	m	b
2.94726E-08	0.000192283	error m	error b
0.995759865	0.000281117	r^2	error Y

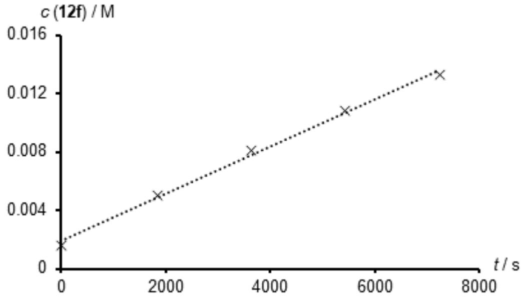

(H3a-F), IDPi **3a**, R = OMe, σ_p = -0.27			
LINEST		SYNTAX (Y=mX + b)	
1.61361E-06	0.001897214	m	b
5.96333E-08	0.000264901	error m	error b
0.995919385	0.00034112	r^2	error Y

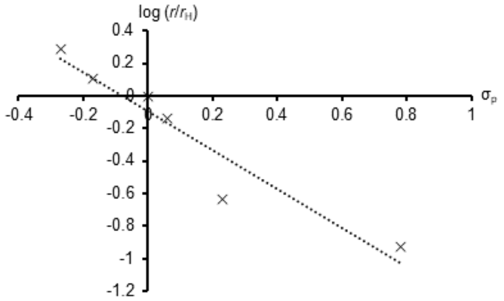

IDPi **2a**, Hammett Plot			
LINEST		SYNTAX (Y=mX + b)	
-1.19606282	-0.09170047	m	b
0.18220582	0.06513274	error m	error b
0.91505753	0.1525042	r^2	error Y

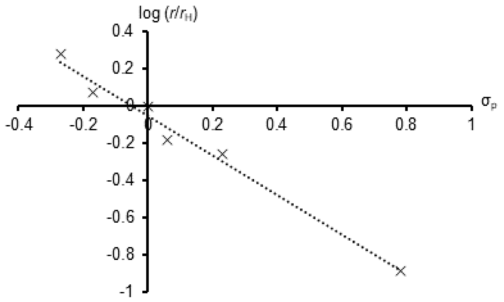

IDPi **3a**, Hammett Plot			
LINEST		SYNTAX (Y=mX + b)	
-1.06579054	-0.04804303	m	b
0.06936968	0.02479744	error m	error b
0.98333682	0.05806164	r^2	error Y

Because it is unusual to obtain initial rate kinetic data up to relatively high conversion of 30%, we re-analysed our dataset in an alternative way only up to 10% conversion (Figure S10). As can be seen, the outcome of our analysis remains essentially unchanged, except for an enhanced error in the linear regressions of the plots.

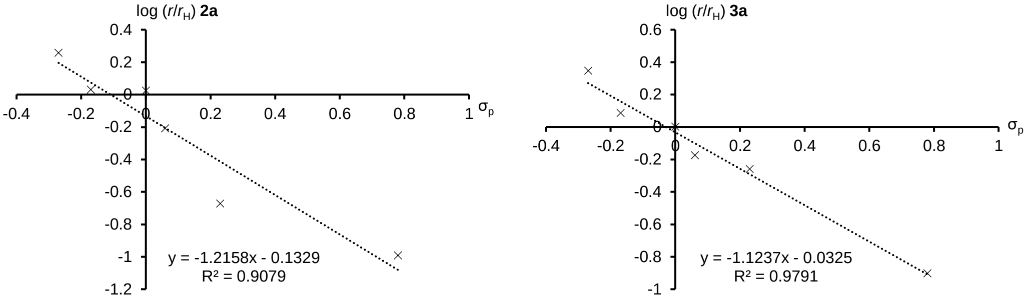

Figure S10 Alternative Hammett plots with data up to 10% conversion.

Kinetic Isotope Effects

A J Young NMR tube was charged with approximately a 1:1 mixture of deuterated and non-deuterated carbamate **7** (12.0 mg total, 0.05 mmol, 1.0 eq.), IDPi **2b** (1.60 mg, 1 mol%), triphenylmethane as internal standard (1.22 mg, 0.005 mmol, 0.10 eq.), and $CDCl_3$ (0.50 mL). The initial deuterium incorporation was determined by ^{1}H NMR spectroscopy. Hexanal or benzaldehyde dimethylacetal (0.065 mmol, 1.3 eq.) was added lastly, and the reaction progress was followed by ^{1}H NMR spectroscopy. Integration of the aromatic signals allowed for determination of the deuteration at each point of the reaction. The measured concentrations were furthermore averaged over three data points to reduce the signal to noise ratio.

$$c_a(6.83\text{–}6.76\text{ ppm}) = c(\mathbf{7_H}) + c(\mathbf{7_D})$$
$$c_b(6.75\text{–}6.65\text{ ppm}) = 2 \times c(\mathbf{7_H}) + c(\mathbf{7_D})$$

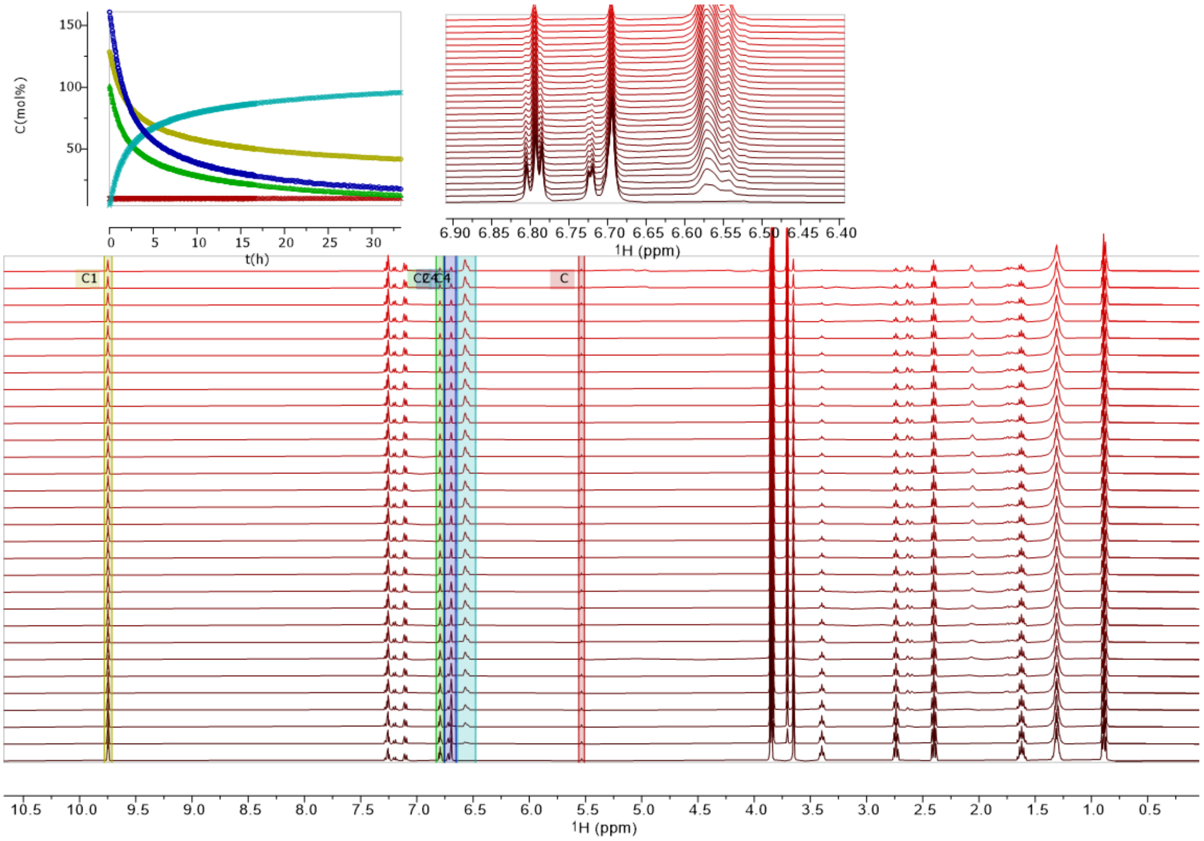

The following formulas were applied to calculate the KIE until every point of measurement (see the data file for a full table):

$X_{\mathrm{H}} = \frac{c_b}{c_a} - 1$	$X_{\mathrm{D}} = 1 - X_{\mathrm{H}}$	$R = \frac{X_{\mathrm{D}}}{X_{\mathrm{H}}}$
$c(\mathbf{7_H}) = c_a \times X_{\mathrm{H}}$	$F_{\mathrm{H}} = 1 - \frac{c(\mathbf{7})}{c_0(\mathbf{7_H})}$	$\mathrm{KIE} = \frac{\ln(1 - F_{\mathrm{H}})}{\ln\left[(1 - F_{\mathrm{H}})\frac{R}{R_0}\right]}$

Hexanal (KIE-A)

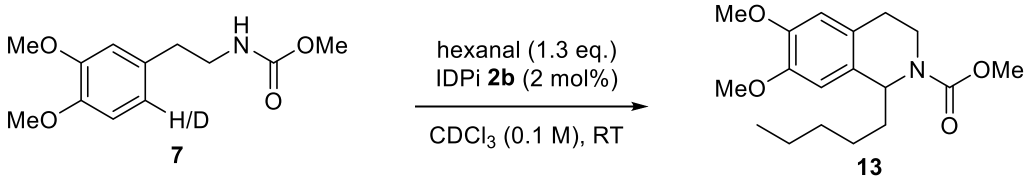

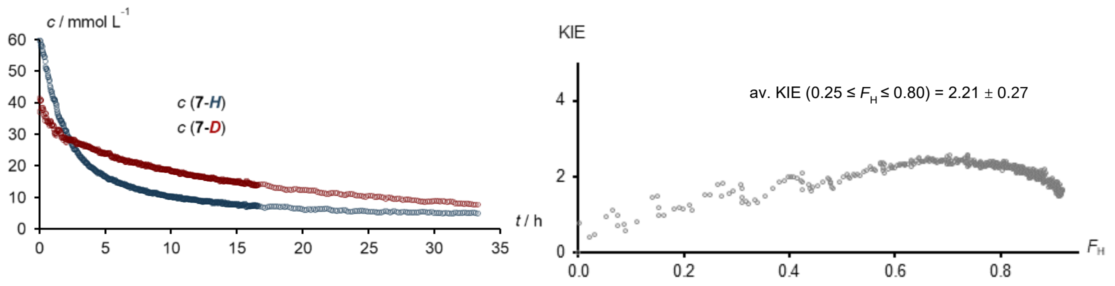

Benzaldehyde Dimethylacetal (KIE-B)

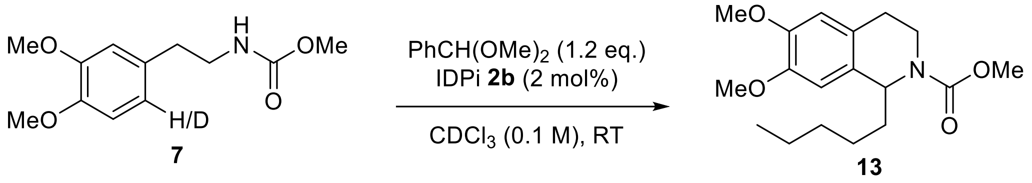

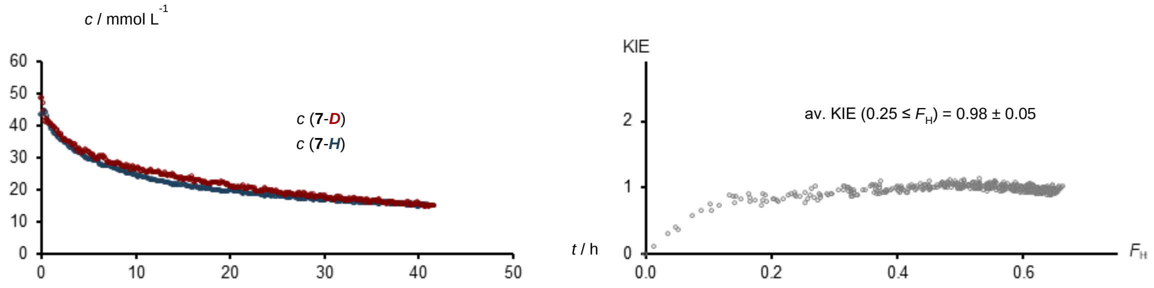

Determination of the Reaction Order

All NMR experiments regarding the reaction order and variable temperature studies were conducted from pre-measured substrate and catalyst samples to improve comparability and reproducibility. Separate GC-vials were filled with stock solutions of IDPi catalyst (0.001 mmol), carbamate **7** (0.10 mmol), or $CHPh_3$ (0.010 mmol) in DCM. The solvent was evaporated at 45 °C, and the samples were dried in a desiccator under reduced pressure. For each reaction, the appropriate amounts of substrate, internal standard, and IDPi catalyst were re-dissolved in dry $CDCl_3$ and mixed in an oven-dried J Young NMR tube. Hexanal was added last, and the reaction progress was monitored by ^{1}H NMR spectroscopy. For reactions at reduced temperatures, the NMR tube was cooled to the reaction temperature before addition of hexanal. Product formation was followed by integration of the newly appearing aromatic signals.

For the generalized rate equation $d[P]/dt = k[A]^{\alpha}[B]^{\beta}[cat]^{\gamma}$, the following formula was applied to normalize the x-axis for components A and B (see the data file for a full table):

$$x(component\ A) = \sum \Delta t \times [\mathrm{A}]^{\alpha} = \sum_{i=1}^{n} \left(\frac{[\mathrm{A}]_i + [\mathrm{A}]_{i-1}}{2} \right)^{\alpha} \times (t_i - t_{i-1})$$

By visual analysis, the respective orders were determined to be:

A = carbamate **7**	B = aldehyde **14**	cat = IDPi **2b**
$\alpha = 0.93$	$\beta = 1.00$	$\gamma = 1.00$

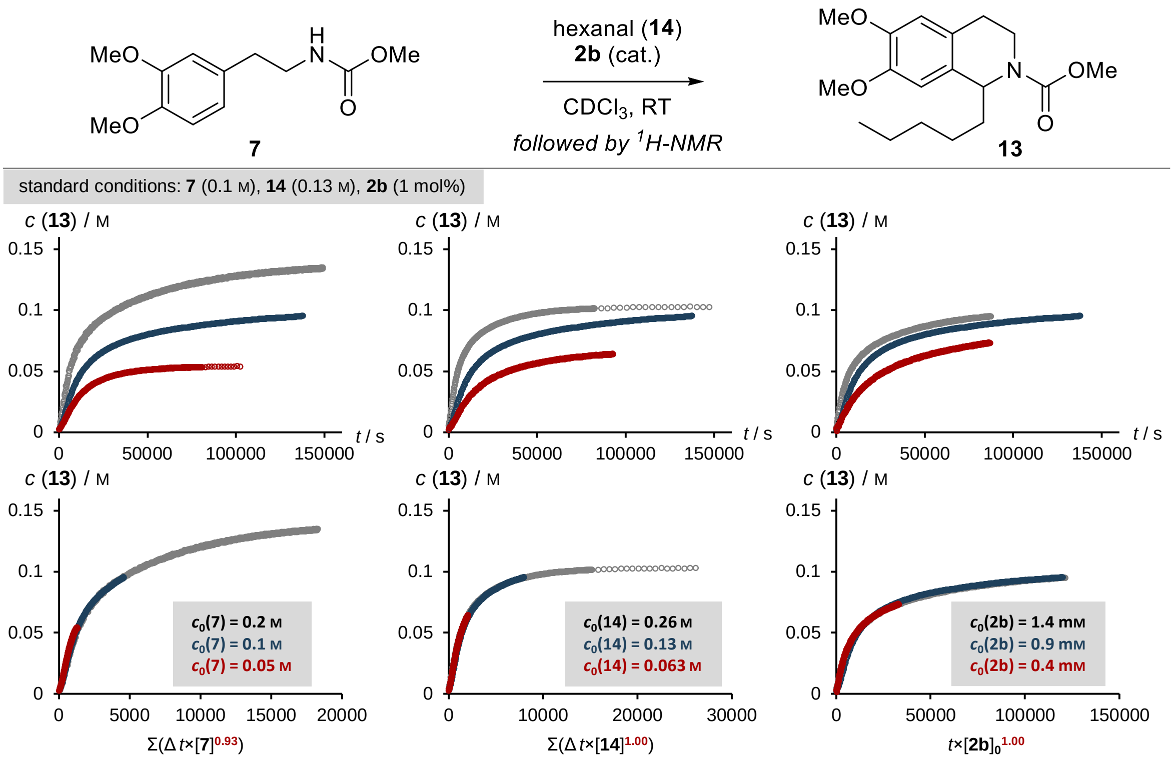

Figure S11 Determination of the reaction order by variable time normalization analysis.

Variable Temperature Studies

Determination of kinetic rate constants *k*

The reaction can be described as an overall catalytic second order reaction with d[P]/d*t* = *k*[A][B][cat]. The kinetic rate constants *k* at different temperatures with IDPi catalyst **2b** and **3a** were determined according to the integrated second order rate law.

$$\frac{\ln\left(\frac{[\mathbf{A}]\times[\mathbf{B}]_0}{[\mathbf{B}]\times[\mathbf{A}]_0}\right)}{[\mathbf{A}]_0-[\mathbf{B}]_0} = [\text{cat}]\times kt$$

The following equations were applied (see the data file for a full table):

$y = kx + b$, with:	$y = \frac{\ln\left(\frac{[\mathbf{14}]_0\times[\mathbf{7}]}{[\mathbf{7}]_0\times[\mathbf{14}]}\right)}{[\mathbf{7}]_0-[\mathbf{14}]_0}$
$x = \sum \Delta t \times [\text{cat}] = \sum_{i=1}^{n}\left(\frac{[\text{cat}]_i + [\text{cat}]_{i-1}}{2}\right)\times(t_i - t_{i-1})$	

The concentrations of carbamate **7** and aldehyde **14** were averaged over two data points, and the concentration of the IDPI catalyst was averaged over five data points to reduce the signal to noise ratio. All concentrations were plotted in mol L^{-1}.

IDPi 2b

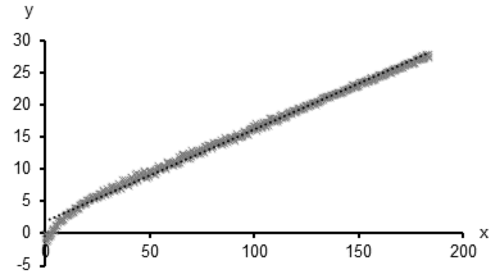

(E2b-A), IDPi **2b**, 283.15 K			
LINEST		SYNTAX (y = kx + b)	
0.14261997	1.859587187	k	b
0.000413981	0.04433924	error m	error b
0.994153313	0.585508233	r^2	error y

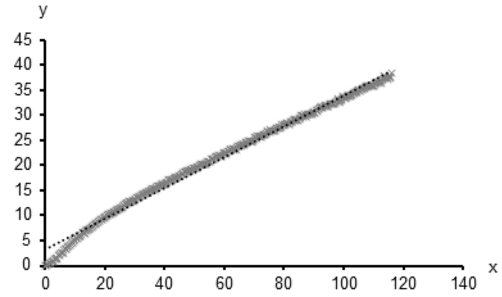

(E2b-B), IDPi **2b**, 298.15 K			
LINEST		SYNTAX (y = kx + b)	
0.308851894	3.089994016	k	b
0.001339269	0.091883591	error m	error b
0.991461595	0.9681706	r^2	error y

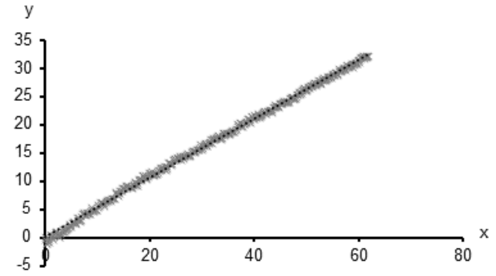

(E2b-C), IDPi **2b**, 308.15 K			
LINEST		SYNTAX (y = kx + b)	
0.523738765	0.035174803	k	b
0.001099121	0.040909993	error m	error b
0.997986962	0.423979379	r^2	error y

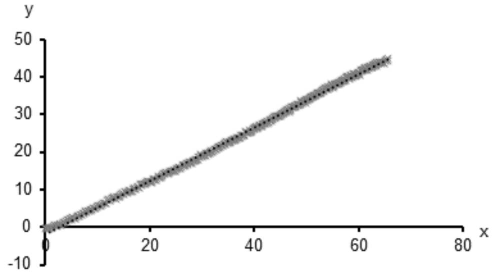

(E2b-D), IDPi **2b**, 318.15 K			
LINEST		SYNTAX (y = kx + b)	
0.712398263	-1.90696619	k	b
0.001029343	0.042779473	error m	error b
0.998753093	0.469339097	r^2	error y

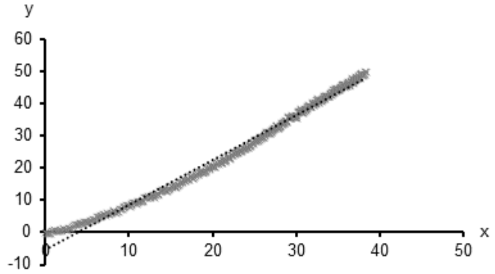

(E2b-E), IDPi **2b**, 333.15 K			
LINEST		SYNTAX (y = kx + b)	
1.388944956	-5.46288868	k	b
0.006919463	0.176330211	error m	error b
0.989003598	1.563858972	r^2	error y

IDPi 3a

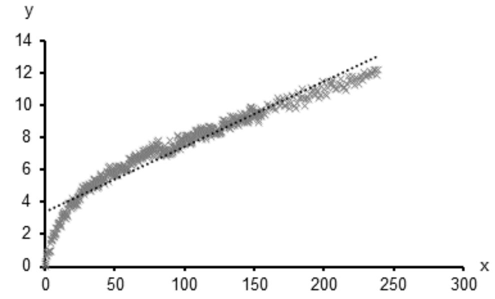

(E3a-A), IDPi **3a**, 283.15 K			
LINEST		SYNTAX (y = kx + b)	
0.040493442	3.379508752	k	b
0.000478909	0.054306746	error m	error b
0.924004349	0.702096477	r^2	error y

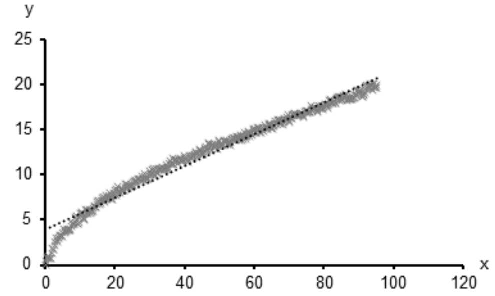

(E3a-B), IDPi **3a**, 298.15 K			
LINEST		SYNTAX (y = kx + b)	
0.176462654	3.908503972	k	b
0.001420758	0.078346635	error m	error b
0.969762534	0.860530385	r^2	error y

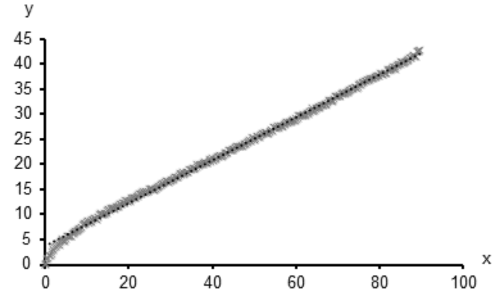

(E3a-C), IDPi **3a**, 308.15 K			
LINEST		SYNTAX (y = kx + b)	
0.428996618	3.615462101	k	b
0.001040204	0.053863195	error m	error b
0.997244322	0.588184044	r^2	error y

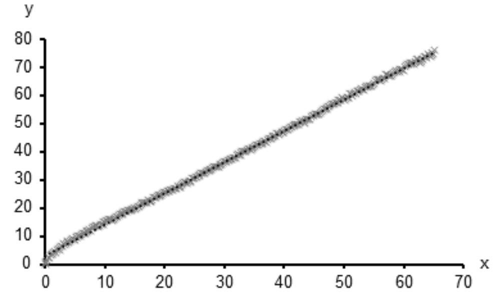

(E3a-D), IDPi **3a**, 318.15 K			
LINEST		SYNTAX (y = kx + b)	
1.10811533	3.168220812	k	b
0.001532776	0.057466915	error m	error b
0.999344162	0.540540412	r^2	error y

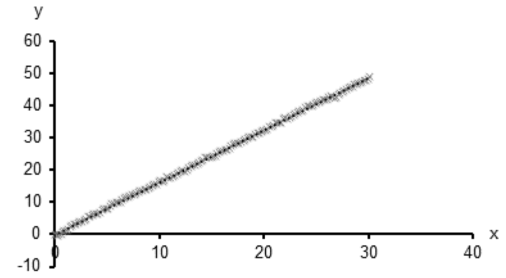

(E3a-E), IDPi **3a**, 333.15 K			
LINEST		SYNTAX (y = kx + b)	
1.620188734	-0.065101005	k	b
0.002540707	0.045960782	error m	error b
0.999596868	0.300216042	r^2	error y

Determination of absolute thermodynamic activation parameters

The kinetic rate constants *k* at each temperature with IDPi catalysts **2b** and **3a** were plotted according to the Eyring equation:

$\ln\left(\frac{kh}{k_B T}\right) = -\frac{\Delta H^{\ddagger}}{R}\frac{1}{T} + \frac{\Delta S^{\ddagger}}{R}$			$R = 8.314\ \frac{\mathrm{J}}{\mathrm{mol \cdot K}}$<br>$h = 6.626 \times 10^{-34}\ \mathrm{J \cdot s}$<br>$k_B = 1.381 \times 10^{-23}\ \frac{\mathrm{J}}{\mathrm{K}}$
$y = mx + b$, with	$y = \ln\left(\frac{kh}{k_B T}\right)$ and	$m = -\frac{\Delta H^{\ddagger}}{R}$ and	$b = \frac{\Delta S^{\ddagger}}{R}$

IDPi 2b

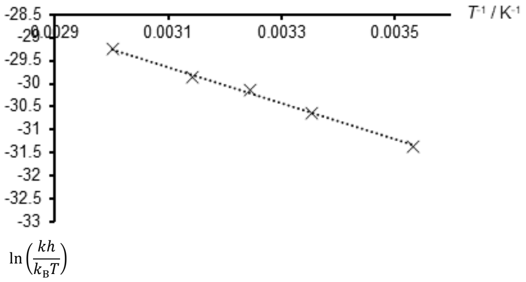

IDPi 2b, Eyring plot			
LINEST		SYNTAX (Y=mx + b)	
-3944.950743	-17.4031599	m	b
131.1039523	0.42741856	error m	error b
0.996697574	0.05295583	r^2	error Y

$\Delta H^{\ddagger}(\mathbf{2b}) = 7.84 \pm 0.26\,\frac{\mathrm{kcal}}{\mathrm{mol}}$	$\Delta S^{\ddagger}(\mathbf{2b}) = -34.58 \pm 0.85\,\frac{\mathrm{cal}}{\mathrm{mol \cdot K}}$
$T\Delta S^{\ddagger}_{298K}(\mathbf{2b}) = -10.31 \pm 0.25\,\frac{\mathrm{kcal}}{\mathrm{mol}}$	$\Delta G^{\ddagger}_{298K}(\mathbf{2b}) = 18.15 \pm 0.51\,\frac{\mathrm{kcal}}{\mathrm{mol}}$

IDPi 3a

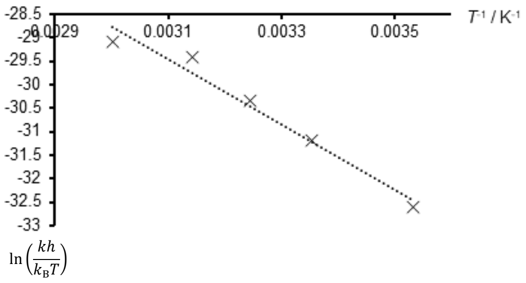

IDPi 3a, Eyring plot			
LINEST		SYNTAX (Y=mx + b)	
-6948.594432	-7.91038664	m	b
718.8334648	2.34350495	error m	error b
0.96889289	0.29035296	r^2	error Y

$\Delta H^{\ddagger}(\mathbf{3a}) = 13.81 \pm 1.43 \frac{\text{kcal}}{\text{mol}}$	$\Delta S^{\ddagger}(\mathbf{3a}) = -15.72 \pm 4.66 \frac{\text{cal}}{\text{mol} \cdot \text{K}}$
$T\Delta S^{\ddagger}_{298K}(\mathbf{3a}) = -4.69 \pm 1.39 \frac{\text{kcal}}{\text{mol}}$	$\Delta G^{\ddagger}_{298K}(\mathbf{3a}) = 18.49 \pm 2.82 \frac{\text{kcal}}{\text{mol}}$

Determination of relative thermodynamic activation parameters

The reactions were conducted according to the general reaction optimization procedure with carbamate **7** (5.98 mg, 0.025 mmol, 1.0 eq.), hexanal (**14**, 4.0 µL, 0.0325 mmol, 1.30 eq.), and IDPi **2b** (0.80 mg, 0.50 µmol, 2 mol%) at temperatures between −30 and 40 °C.

***T* / °C**	**er**	**ln(er)**
−30	95.960:4.040	3.168
−20	95.495:4.505	3.054
−10	94.943:5.057	2.933
0	94.336:5.664	2.813
10	93.736:6.264	2.706
20	93.241:6.759	2.624
30	93.146:6.854	2.609
40	92.572:7.428	2.523

The relative kinetic rate constants *k*(*R*)/*k*(*S*) at each temperature with IDPi catalyst **2b** were plotted according to the Eyring equation:

$\ln\left(\frac{k(R)}{k(S)}\right) = \ln(er) = -\frac{\Delta\Delta H^{\ddagger}}{R}\frac{1}{T} + \frac{\Delta\Delta S^{\ddagger}}{R}$			$R = 8.314 \frac{\text{J}}{\text{mol} \cdot \text{K}}$<br>$h = 6.626 \times 10^{-34}\ \text{J} \cdot \text{s}$<br>$k_{\text{B}} = 1.381 \times 10^{-23}\ \frac{\text{J}}{\text{K}}$
$y = mx + b$, with	$y = \ln(er)$ and	$m = -\frac{\Delta\Delta H^{\ddagger}}{R}$ and	$b = \frac{\Delta\Delta S^{\ddagger}}{R}$

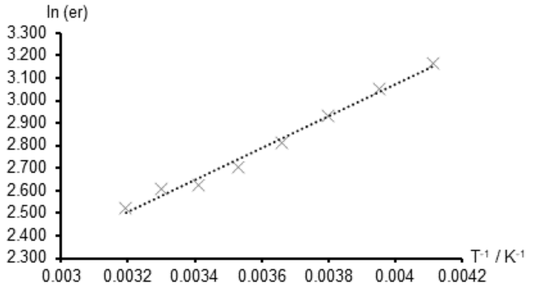

IDPi 2b, Eyring plot			
LINEST		SYNTAX (Y=mx + b)	
711.29600731	.22880404	m	b
32.89761699	.11949495	error m	error b
.98732812	.02796407	r^2	error Y

$\Delta\Delta H^{\ddagger}(\mathbf{2b}) = -1.41 \pm 0.065 \frac{\text{kcal}}{\text{mol}}$	$\Delta\Delta S^{\ddagger}(\mathbf{2b}) = -0.45 \pm 0.24 \frac{\text{cal}}{\text{mol} \cdot \text{K}}$
$T\Delta\Delta S^{\ddagger}_{298K}(\mathbf{2b}) = -0.14 \pm 0.071 \frac{\text{kcal}}{\text{mol}}$	$\Delta G^{\ddagger}_{298K}(\mathbf{2b}) = -1.55 \pm 0.14 \frac{\text{kcal}}{\text{mol}}$

8. Computational Studies

Possible transition state conformations were explored by the artificial force induced reaction (AFIR) method[10] implemented in the global route reaction mapping (GRRM) program.[11] An extensive conformational search has been performed on possible catalyst substrate orientations at GFN2-xTB level of theory[12] implemented in Orca 4.2.1,[13] using SC-AFIR with constraint. Molecular geometries were optimized at r2SCAN-3c[14] implemented in Orca 5.0.3 program, in a gas phase.[15,16] In the case of transition state optimization, they were combined with GRRM program. Thermal free energy corrections have been performed at the same level of theory using Orca 5.0.3 program, and the temperature was set at 298.15 K. Transition state structures were verified by the presence of a single imaginary vibrational frequency and the corresponding intrinsic reaction coordinates. Solvation effect has been accounted by using CPCM ($CHCl_3$) solvation model[17] as implemented in Orca 5.0.3 program. All single point energies were calculated at CPCM($CHCl_3$)-ωB97M-V/(ma)-def2-TZVPP level of theory.[18] The (ma)-def2-TZVPP basis set refers to ma-def2-TZVPP[19] on P, N, S, and O in the catalytic active site, and def2-TZVPP[20] on all others for a better evaluation of anionic species. RI approximation was used with AutoAux option implemented in Orca 5.0.3. IGMH analysis was performed using Multiwfn[21,22] with VMD for visualization.[23] The wavefunction file was generated at ωB97XD/def2-TZVPP level of theory using Gaussian 16.[24] Conversion of enantiomeric ratio and $\Delta\Delta G$ was performed based on the Boltzmann distribution as follows: $\Delta\Delta G = RT\ \ln(\text{pdt}(R)/\text{pdt}(S))$.[25]

To elucidate key interactions in the enantiodetermining steps, IGMH analysis was performed using Multiwfn program. Intrinsic bond strength index for weaker bonds (IBSIW) was also calculated with the same program. The key interactions are depicted in Figure S12. By comparing two diastereomers leading to the major enantiomer, ***cis*-TS4** and ***trans*-TS4**, the main difference appears to stem from the lack of interactions. While the acidic C-H bonds from arenium interact with the electronically enriched benzofuran in both cases, the orientation of the arenium hinders efficient interactions in ***trans*-TS-4**. For instance, the largest IBSIW indices for fluorine atoms are 1.20 for ***cis*-TS4** and 0.47 for ***trans*-TS4**, respectively, indicating that the former has more interactions between the substrate and fluorine atoms, as also evident from the IGMH map. When comparing two diastereomers leading to the minor enantiomer, ***cis*-TS4'** and ***trans*-TS4'**, the main difference seems to arise again from the lack of interactions. While the interactions of C-H bonds from arenium are very similar between the two isomers, the nitrogen in the catalytic active site lacks interactions in ***trans*-TS-4**. IBSIW indices also support this hypothesis, and the contribution of the corresponding nitrogen are 1.39 for ***cis*-TS4'** and 0.45 for ***trans*-TS4'**, respectively, as again evident from the IGMH map.

To obtain further insights into the origin of stereoselectivity, distortion interaction analysis was conducted following the Houk-Bickelhaupt protocol.[26] The gas phase electronic energy of the optimized TS structures has been decomposed into the catalyst counteranion and the substrate at ωB97M-V/(ma)-def2-TZVPP level of theory (Table S5–Table S7). When ***cis*-TS4** and ***trans*-TS4'** are compared, the latter has more interactions between the two fragments. Meanwhile, each fragment, especially the substrate, is more stable in the former case, which appears to be the predominant factor controlling overall selectivity. This indicates that the substrate is distorted to a twist-boat-like structure in ***trans*-TS4'** to maximize the interactions

between the two fragments. When comparing ***cis*-TS4** vs ***trans*-TS4**, the interaction overrides the distortion effect, which is consistent with the IGMH analysis. The same was observed in the comparison between ***trans*-TS4'** and ***cis*-TS4'**; the interaction is suggested to be the major factor controlling stereoselectivity.

The Cartesian coordinates of all optimized structures, as well as the IGMH analysis are attached as tables in individual tabstop-separated txt files. The files are named as follows:

**Cartesian Coordinates**	**Data file**	**Structure**
	geom_7	**7**
	geom_16	**16**
	geom_2b	**2b**
	geom_H2O	**H_2O**
	geom_I	**I**
	geom_TS1	**TS1**
	geom_II	**II**
	geom_TS2	**TS2**
	geom_III	**III**
	geom_IV	**IV**
	geom_TS3	**TS3**
	geom_cis-V	***cis*-V**
	geom_trans-V	***trans*-V**
	geom_cis-V'	***cis*-V'**
	geom_trans-V'	***trans*-V'**
	geom_cis-TS4	***cis*-TS4**
	geom_trans-TS4	***trans*-TS4**
	geom_cis-TS4'	***cis*-TS4'**
	geom_trans-TS4'	***trans*-TS4'**
	geom_VI	**VI**
**IBSIW Analysis**	**Data file**	**Structure**
	IBSIW_cis-TS4	***cis*-TS4**
	IBSIW_trans-TS4	***trans*-TS4**
	IBSIW_cis-TS4'	***cis*-TS4'**
	IBSIW _trans-TS4'	***trans*-TS4'**

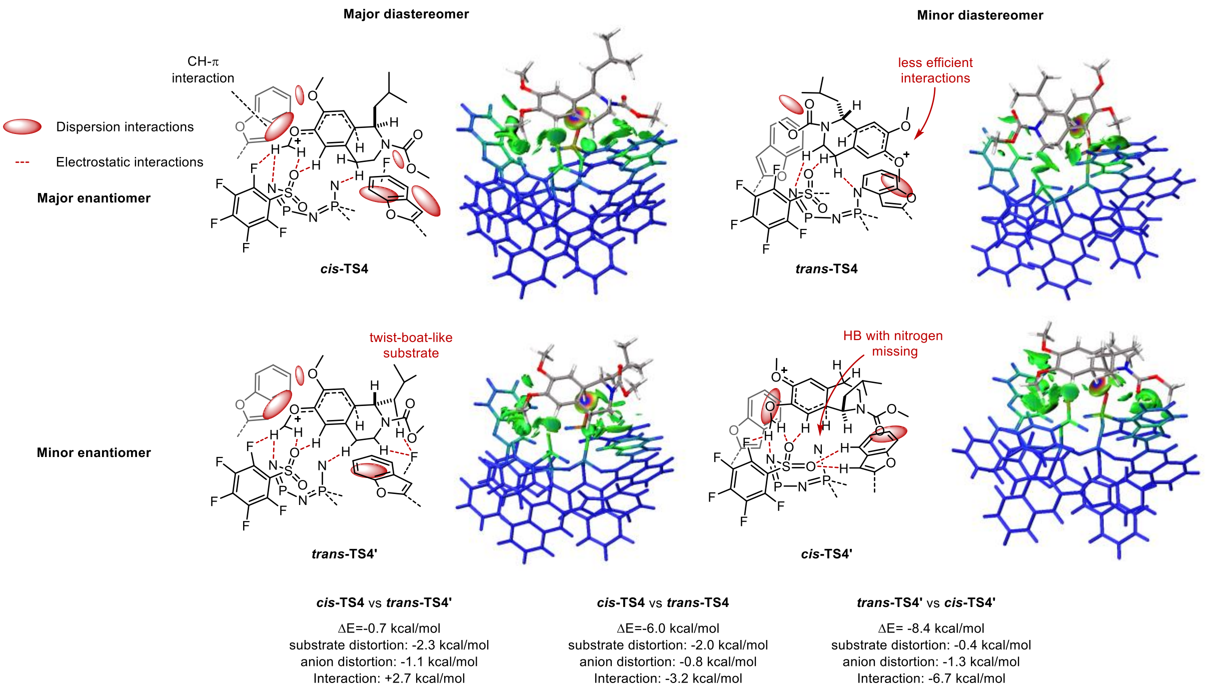

Figure S12 Summary of the four transition states for **TS4** and their comparison. For IGMH analysis, isosurface value is set as 0.005.

Table S5 Distortion-interaction analysis (***cis*-TS4** vs ***trans*-TS4'**).

TS No.	ωB97M-V/(ma)-def2-TZVPP single point energy (in hartree)	Relative energy Δ*E* / kcal mol^{-1}
***cis*-TS4**	-7789.305009	-0.68
***trans*-TS4'**	-7789.303928	
Only substrate		
***cis*-TS4_subst**	-1018.828809	-2.30
***trans*-TS4'_subst**	-1018.825141	
Only catalyst anion		
***cis*-TS4_anion**	-6770.293797	-1.10
***trans*-TS4'_anion**	-6770.292038	
Total distortion	(predominant factor)	-3.41
Total interaction		+2.73

Table S6 Distortion-interaction analysis (***cis*-TS4** vs ***trans*-TS4**).

TS No.	ωB97M-V/(ma)-def2-TZVPP single point energy (in hartree)	Relative energy Δ*E* / kcal mol^{-1}
***cis*-TS4**	-7789.305009	-5.96
***trans*-TS4**	-7789.295508	
Only substrate		
***cis*-TS4_subst**	-1018.828809	-1.95
***trans*-TS4_subst**	-1018.825696	
Only catalyst anion		
***cis*-TS4_anion**	-6770.293797	-0.75

***trans*-TS4_anion**	-6770.292597	
Total distortion		-2.70
Total interaction	(predominant factor)	-3.25

Table S7 Distortion-interaction analysis (***trans*-TS4'** vs ***cis*-TS4'**).

TS No.	ωB97M-V/(ma)-def2-TZVPP single point energy (in hartree)	Relative energy ΔE / kcal mol^{-1}
***trans*-TS4'**	-7789.303928	-8.37
***cis*-TS4'**	-7789.290593	
Only substrate		
***trans*-TS4'_subst**	-1018.825141	-0.37
***cis*-TS4'_subst**	-1018.824557	
Only catalyst anion		
***cis*-TS4'_anion**	-6770.292038	-1.25
***trans*-TS4'_anion**	-6770.290042	
Total distortion		-1.62
Total interaction	(predominant factor)	-6.75

Table S8 Energy table of the optimized structures. Energies are given in Hartree. Computed single point energies (E), Gibbs free energy corrections (G_{corr}), Gibbs free energies (G), and imaginary frequencies for transition states are provided.

Structures	E (solv) (in Hartree)	G_{corr} (at 298.15 K)	G (solv) (in Hartree)	Imaginary Frequency
**7**	-823.1982779	0.23825969	-822.9600182	-
**16**	-271.7509111	0.10880591	-271.6421052	-
**2b**	-6770.826433	0.95775485	-6769.868679	-
**H_2O**	-76.44121318	0.00378546	-76.43742772	-
**I**	-7865.82104	1.35497789	-7864.466062	-
**TS1**	-7865.813887	1.3603823	-7864.453505	-23.50984515
**II**	-7865.830605	1.36519994	-7864.465405	-
**TS2**	-7865.818073	1.36148737	-7864.456586	-967.63607172
**III**	-7865.82986	1.36124	-7864.468625	-
**IV**	-7789.373084	1.336145	-7788.036939	-
**TS3**	-7789.358629	1.33769015	-7788.020939	-200.81548521
***cis*-V**	-7789.366426	1.33729354	-7788.029132	-
***trans*-V**	-7789.357939	1.33748132	-7788.020457	-
***cis*-V'**	-7789.361415	1.33953905	-7788.021876	-
***trans*-V'**	-7789.366376	1.33801853	-7788.028357	-
***cis*-TS4**	-7789.349016	1.33356852	-7788.015447	-814.95835426
***trans*-TS4**	-7789.342033	1.33301047	-7788.009023	-717.53090742
***cis*-TS4'**	-7789.334724	1.33511818	-7787.999606	-820.83490550
***trans*-TS4'**	-7789.346911	1.33362531	-7788.013286	-900.30582806
**VI**	-7789.364015	1.33688042	-7788.027135	-
**pdt**	-1018.532766	0.35185127	-1018.180914	-

We were interested in assessing the effect of the benzofuran units on the counteranion stability in ***cis*-TS4**, as this could serve as a valuable insight into the acidifying effects of the 2-benzofuranyl substituents. We hypothesized that intramolecular electrostatic interactions might play a significant role. To verify this theory, the interactions between the 2-benzofuranyl substituents and the sulfonamide core of the catalyst were visualized by IGMH analysis (Figure S13). Relatively strong noncovalent interactions were observed between the C-H of benzofuran and the Lewis basic sulfonyl oxygens (distance: 2.49 Å and 2.42 Å each), presumably due to the polarization of the C-H bonds in the benzofuran substituents. Considering these observations, intramolecular electrostatic interaction seem to play a role in stabilizing the IDPi counteranion.

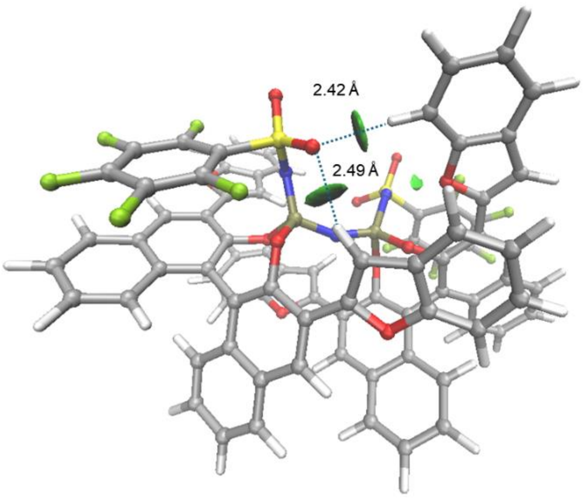

Figure S13 IGMH analysis of ***cis*-TS4** anion, visualizing NCIs between the benzofuran substituents and the core sulfonamide moieties.

To further probe the effect of the solvent polarity on the enantioselectivity, we systematically conducted a study using the CPCM solvation model, demonstrating that all the values fall within a reasonable range of enantioselectivities (Table S9). All the described energies are ΔE in kcal/mol at the CPCM(solvent)-wB97M-V/(ma)-def2-TZVPP level of theory. As diethyl ether is not listed in the solvent model, e=4.33 and refractive Index= 1.3524 were used.

Table S9 Evaluation of solvent effects using the CPCM model.

	Gas phase	Hexane ε=1.89	Toluene ε=2.4	Et_2O ε=4.33	$CHCl_3$ ε=4.9	THF ε=7.25	CH_2Cl_2 ε=9.08
***cis*-TS4**	0.0	0.0	0.0	0.0	0.0	0.0	0.0
***trans*-TS4**	5.96	5.08	4.85	4.44	4.38	4.23	4.16
***cis*-TS4'**	9.05	9.24	9.20	9.01	8.97	8.83	8.77
***trans*-TS4'**	0.68	1.02	1.12	1.29	1.32	1.39	1.42

9. NMR Spectra

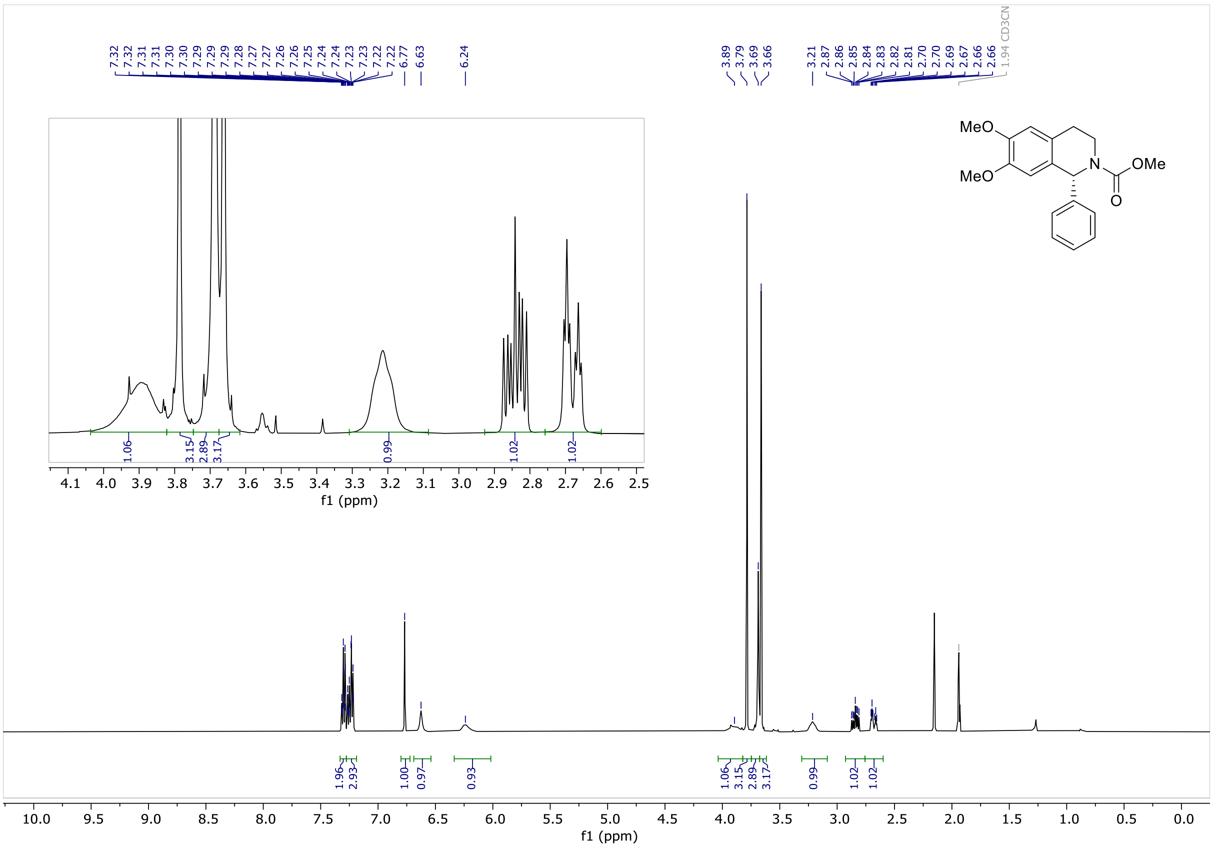

^{1}**H NMR** spectrum of compound **18a**.

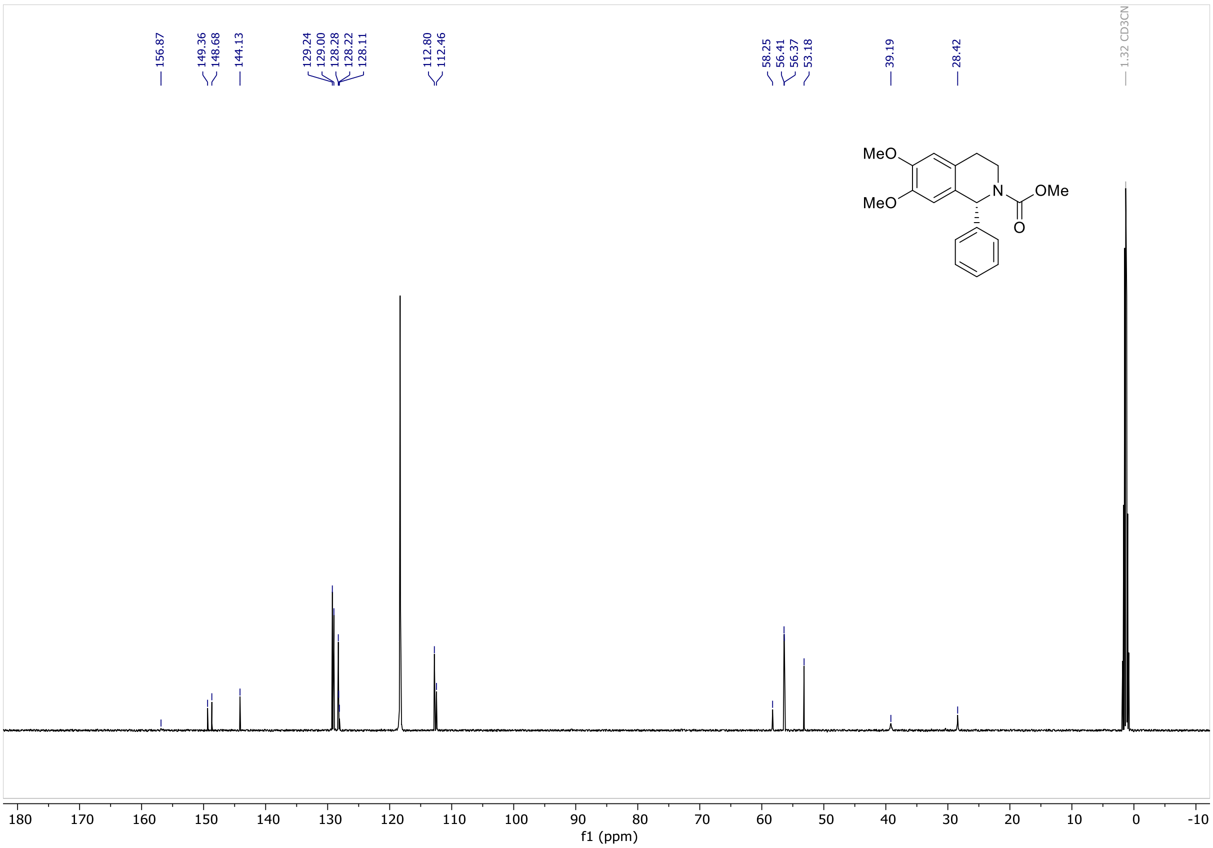

^{13}**C NMR** spectrum of compound **18a**.

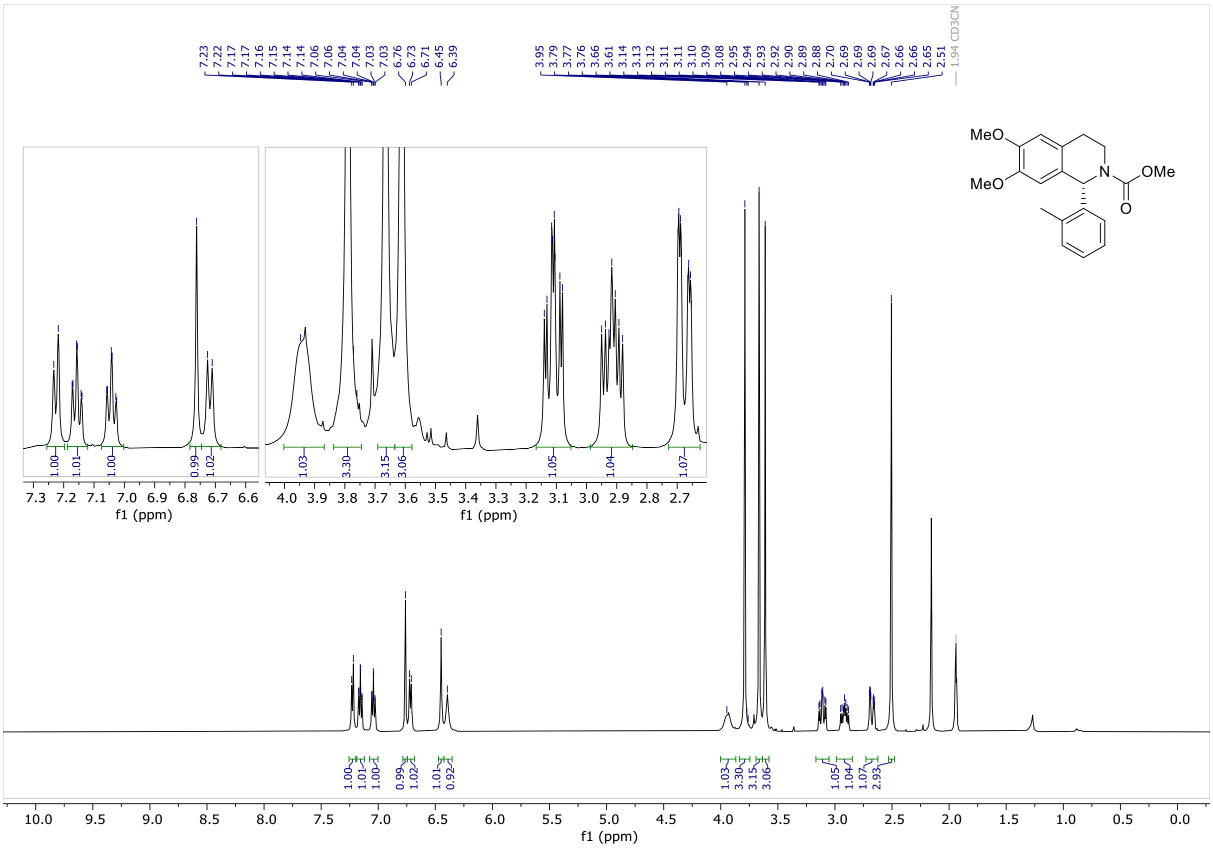

^{1}H NMR spectrum of compound **18b**.

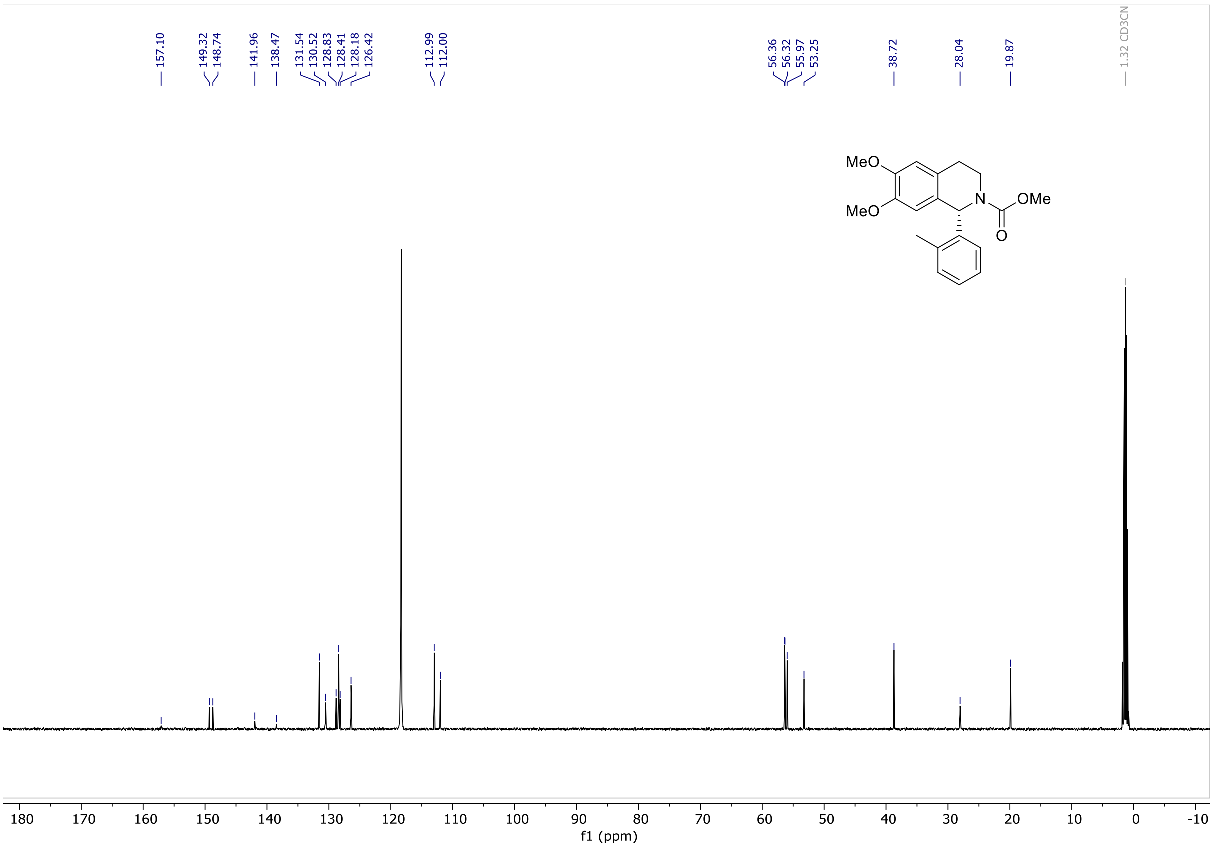

^{13}C NMR spectrum of compound **18b**.

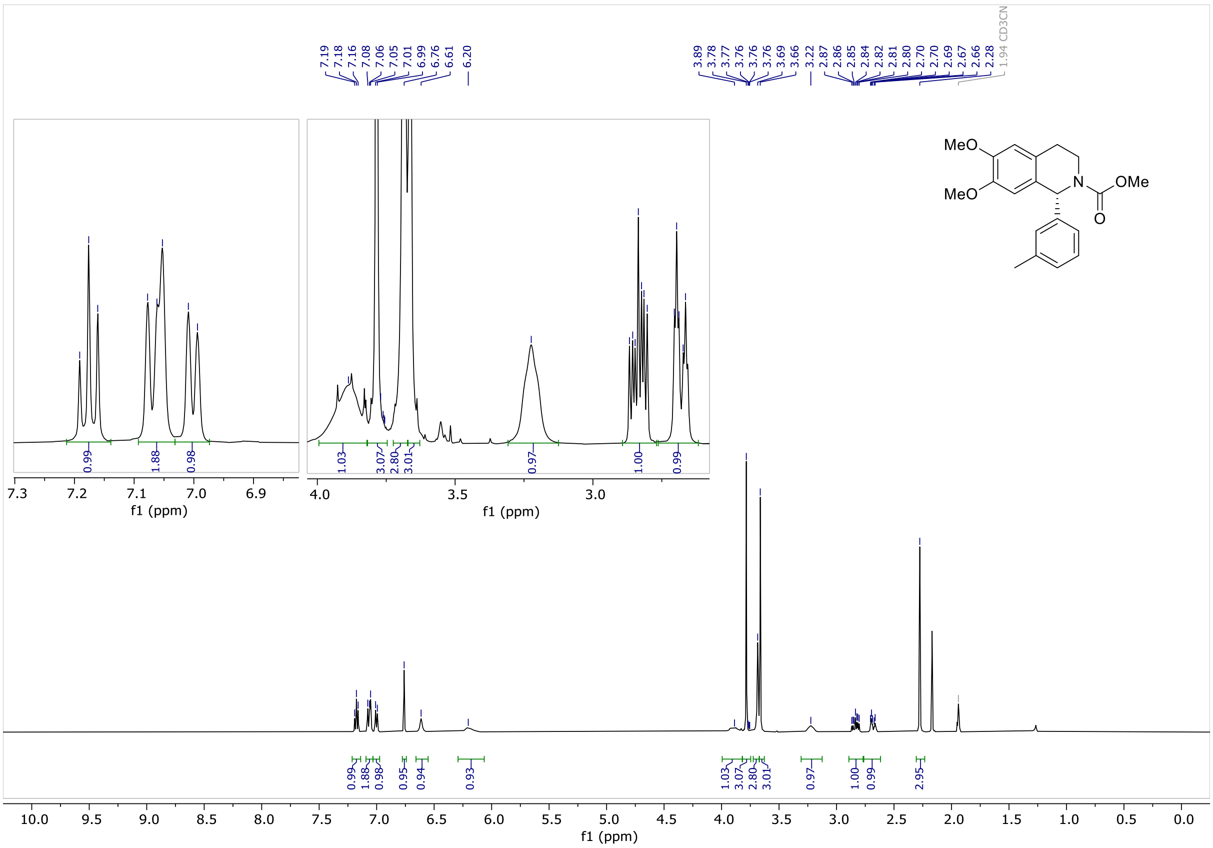

^{1}H NMR spectrum of compound **18c**.

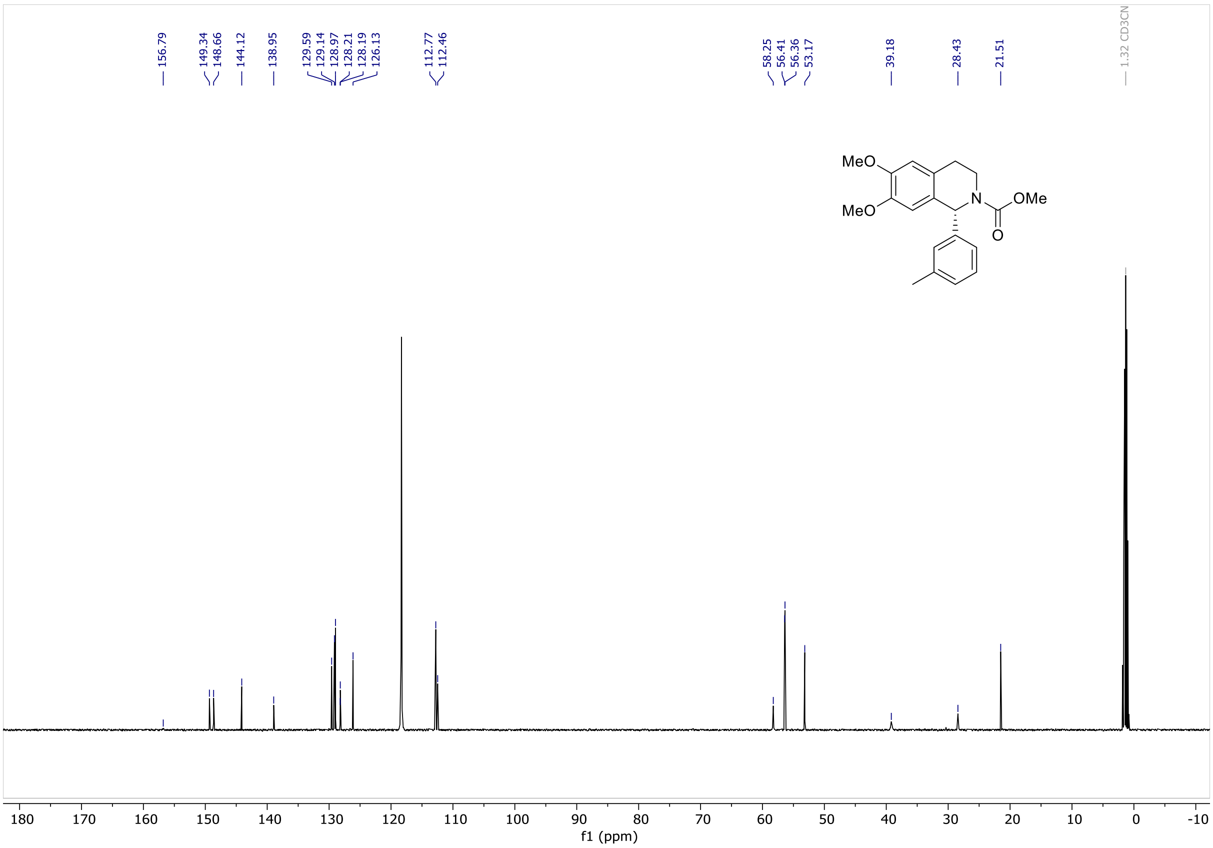

^{13}C NMR spectrum of compound **18c**.

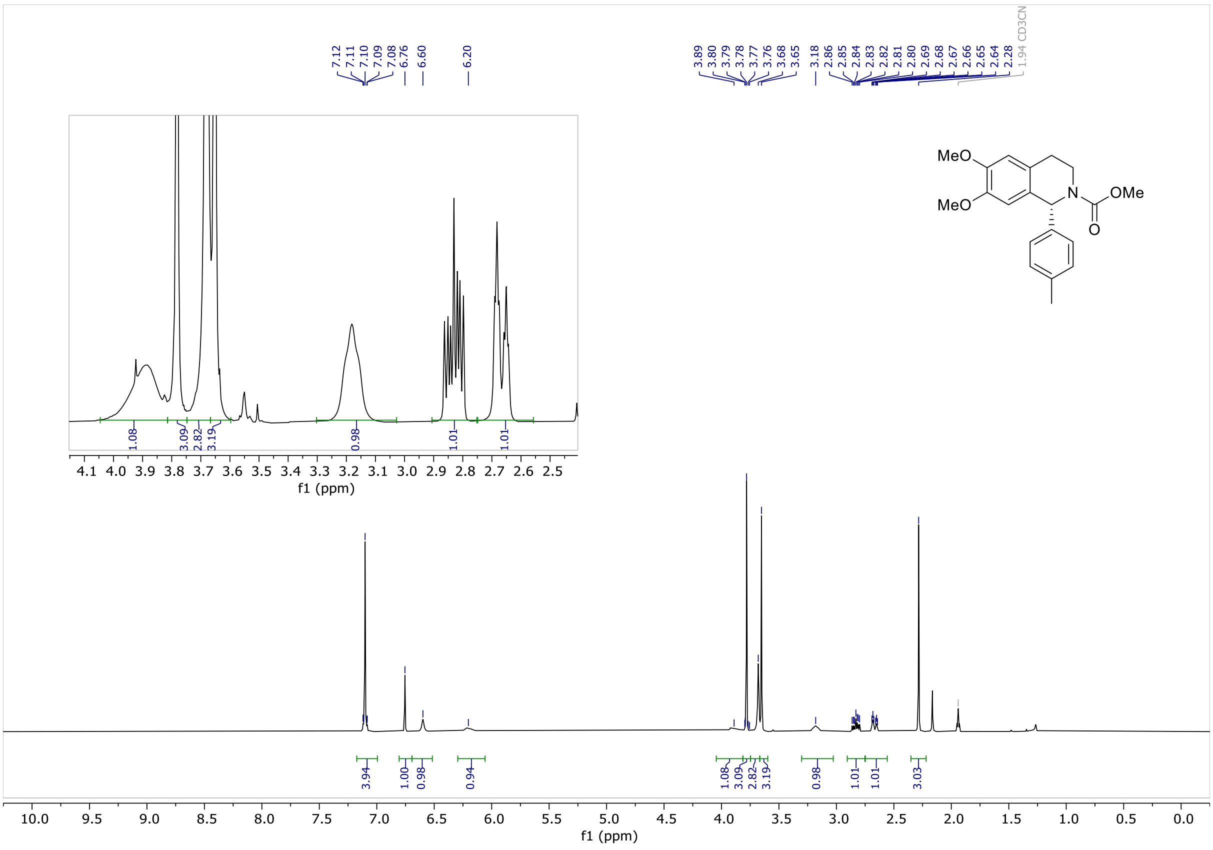

^{1}H NMR spectrum of compound **18d**.

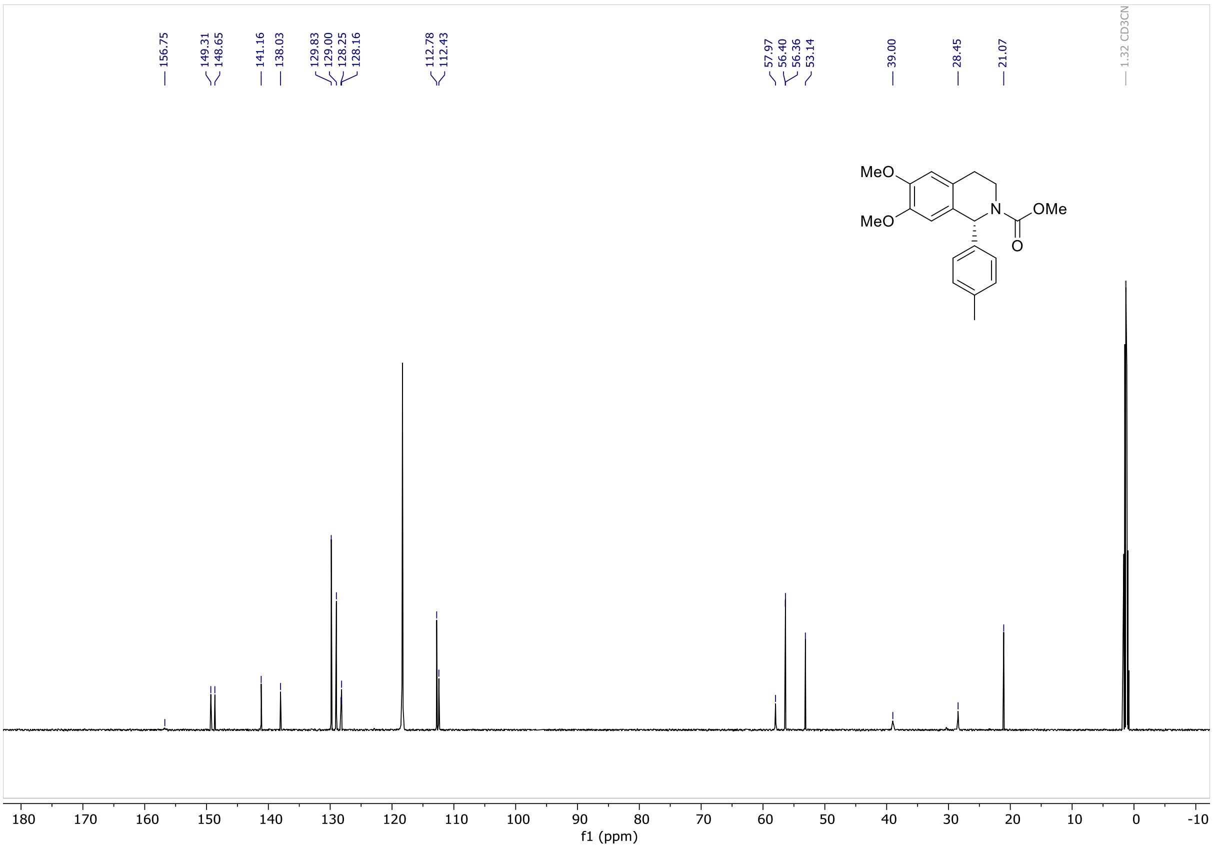

^{13}C NMR spectrum of compound **18d**.

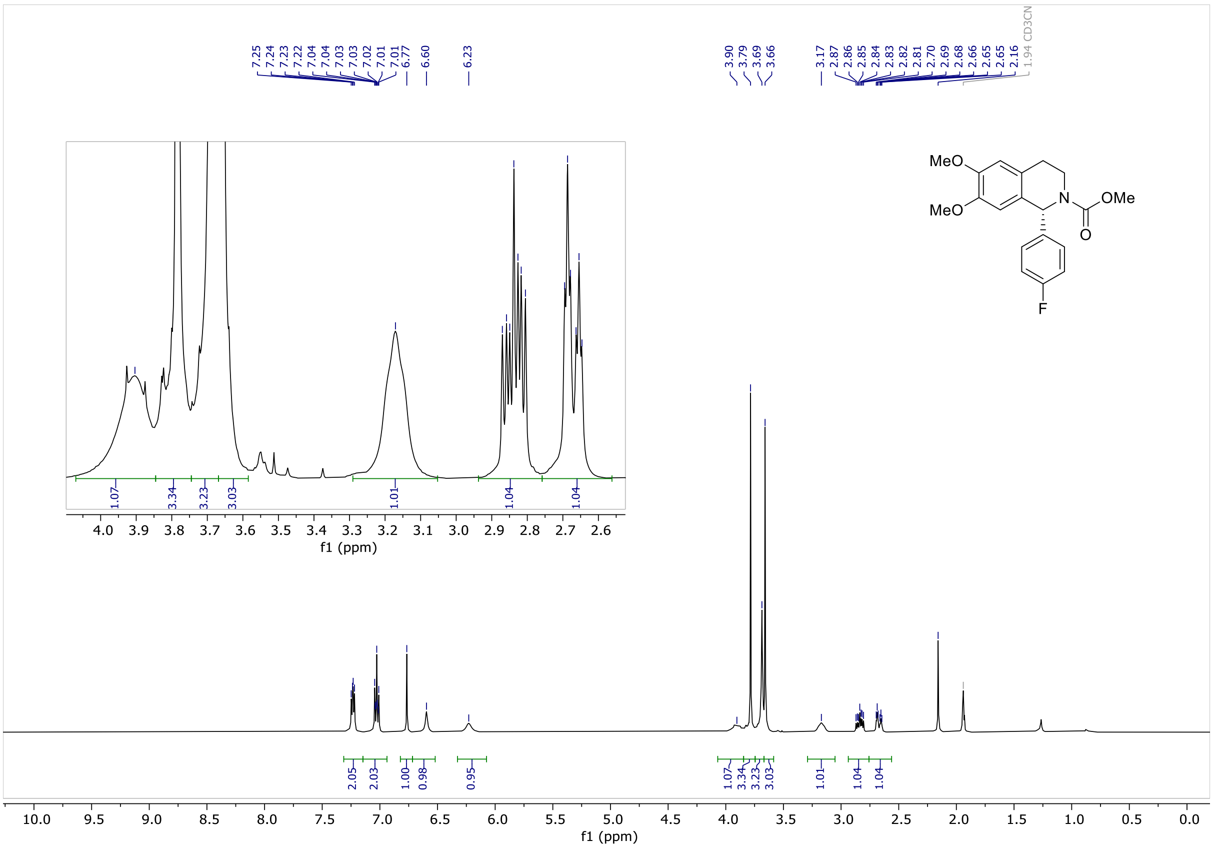

^{1}H NMR spectrum of compound **18e**.

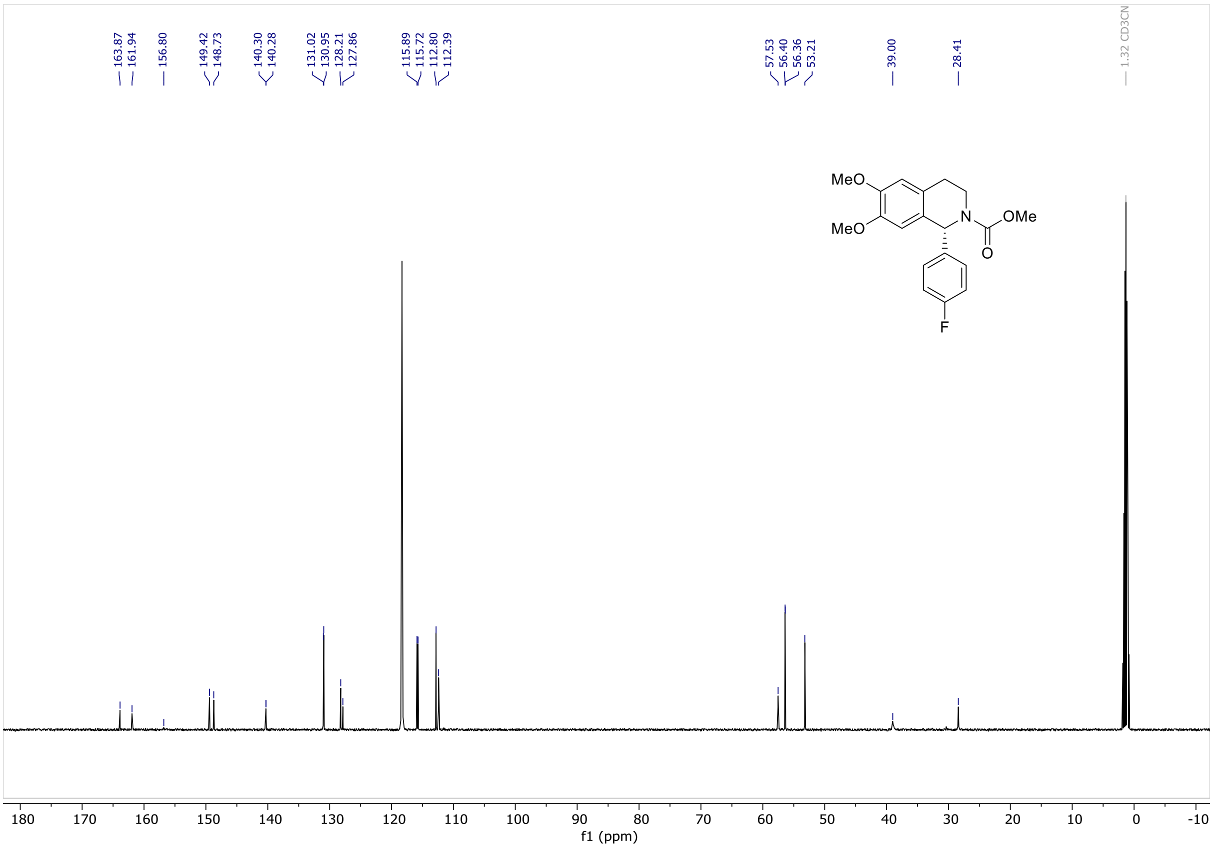

^{13}C NMR spectrum of compound **18e**.

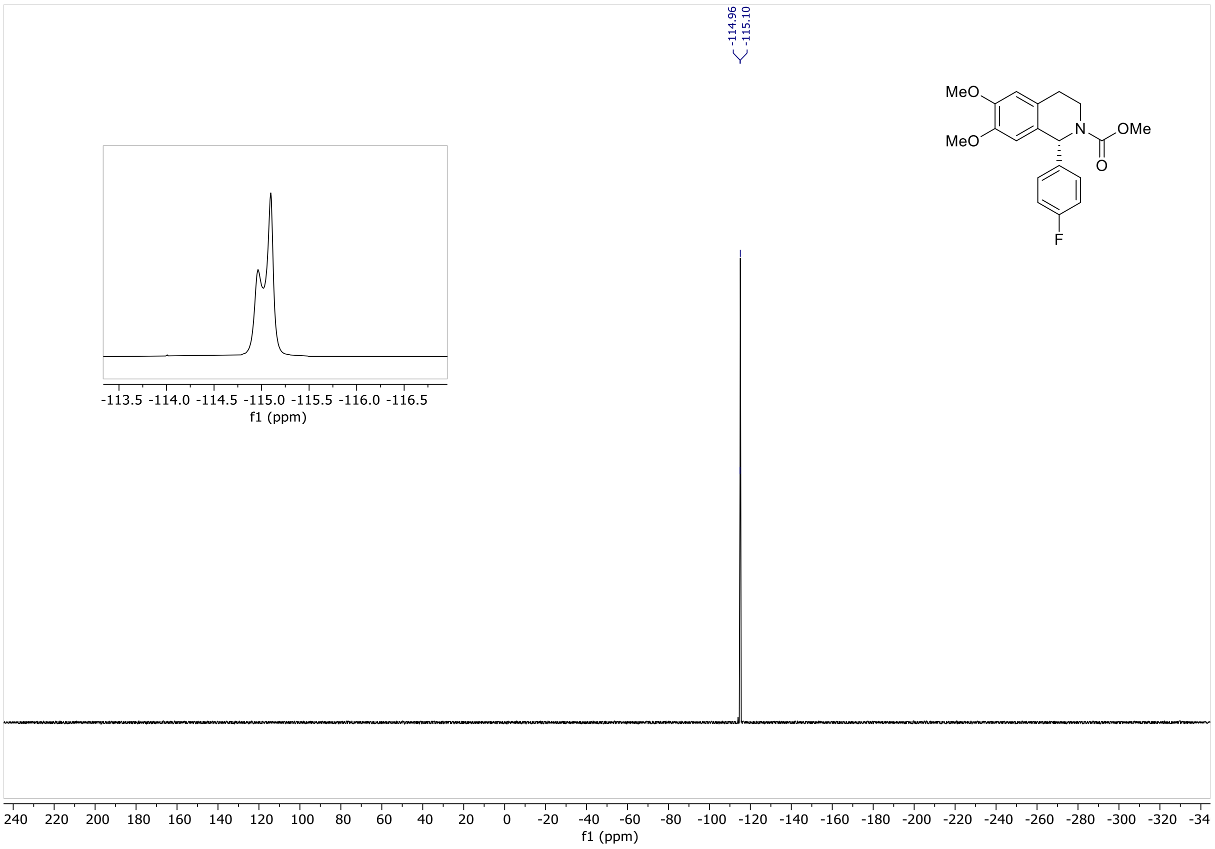

^{19}F NMR spectrum of compound **18e**.

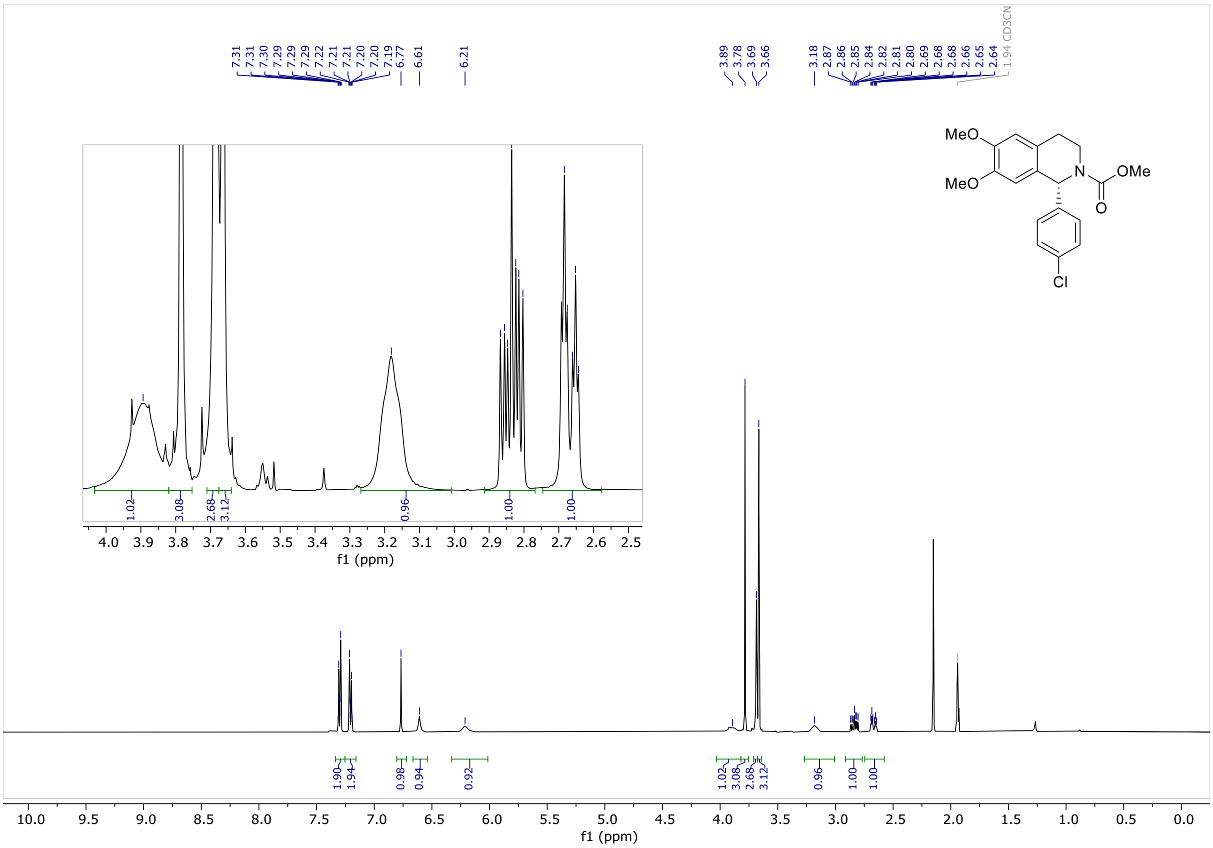

^{1}H NMR spectrum of compound **18f**.

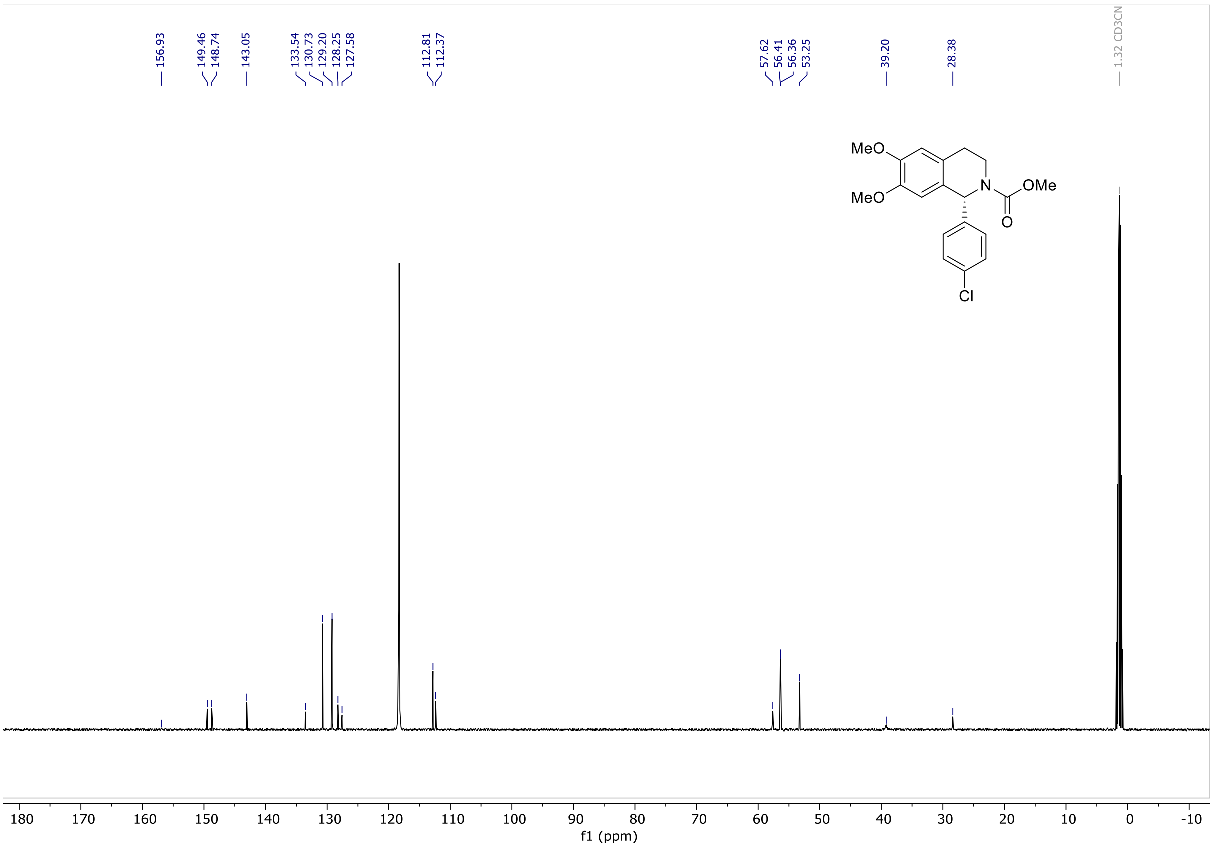

^{13}C NMR spectrum of compound **18f**.

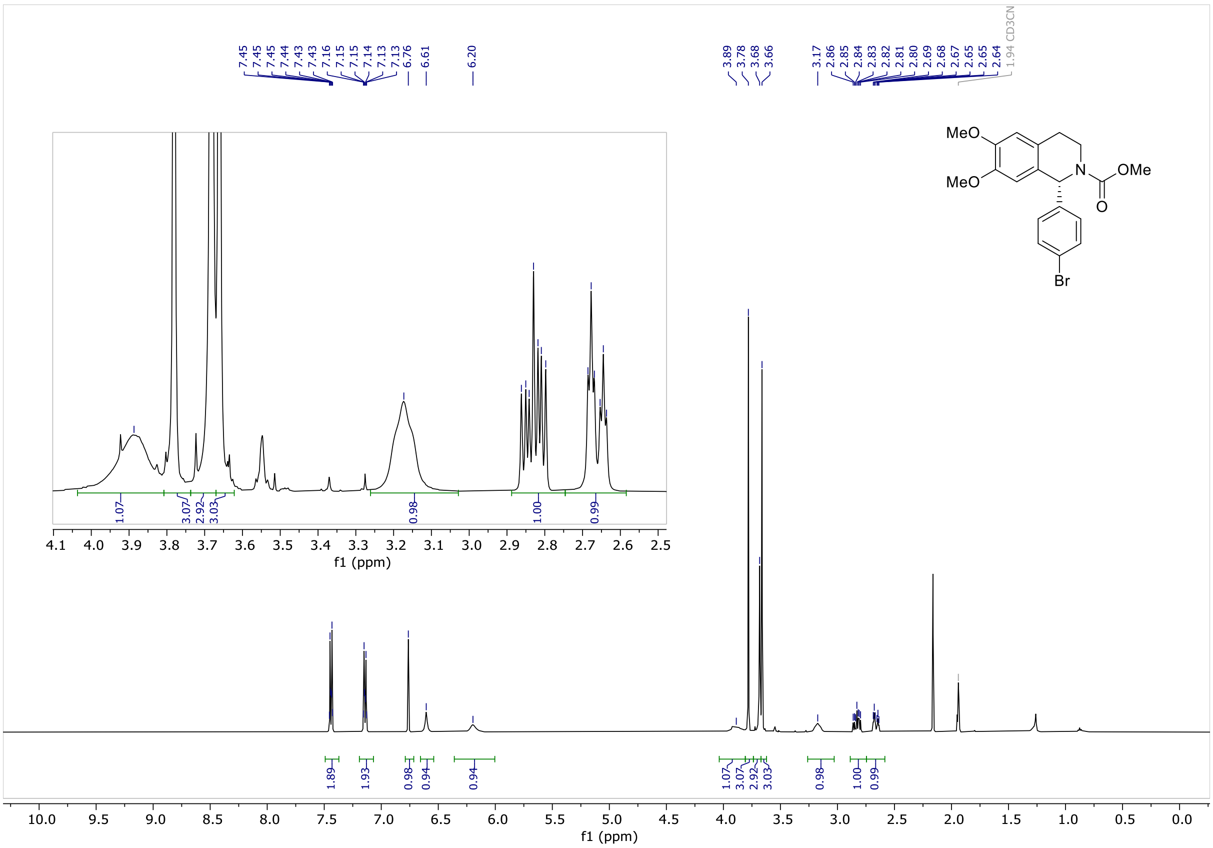

^{1}H NMR spectrum of compound **18g**.

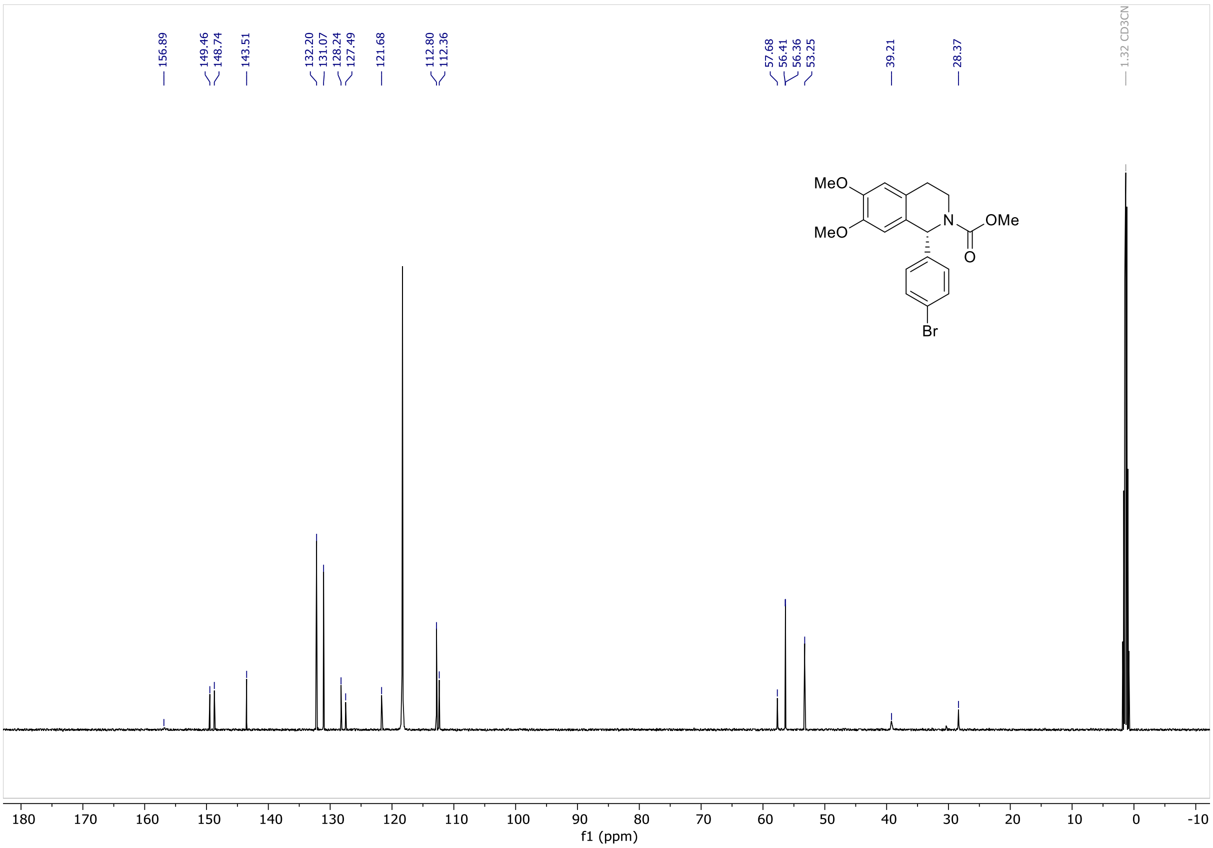

^{13}C NMR spectrum of compound **18g**.

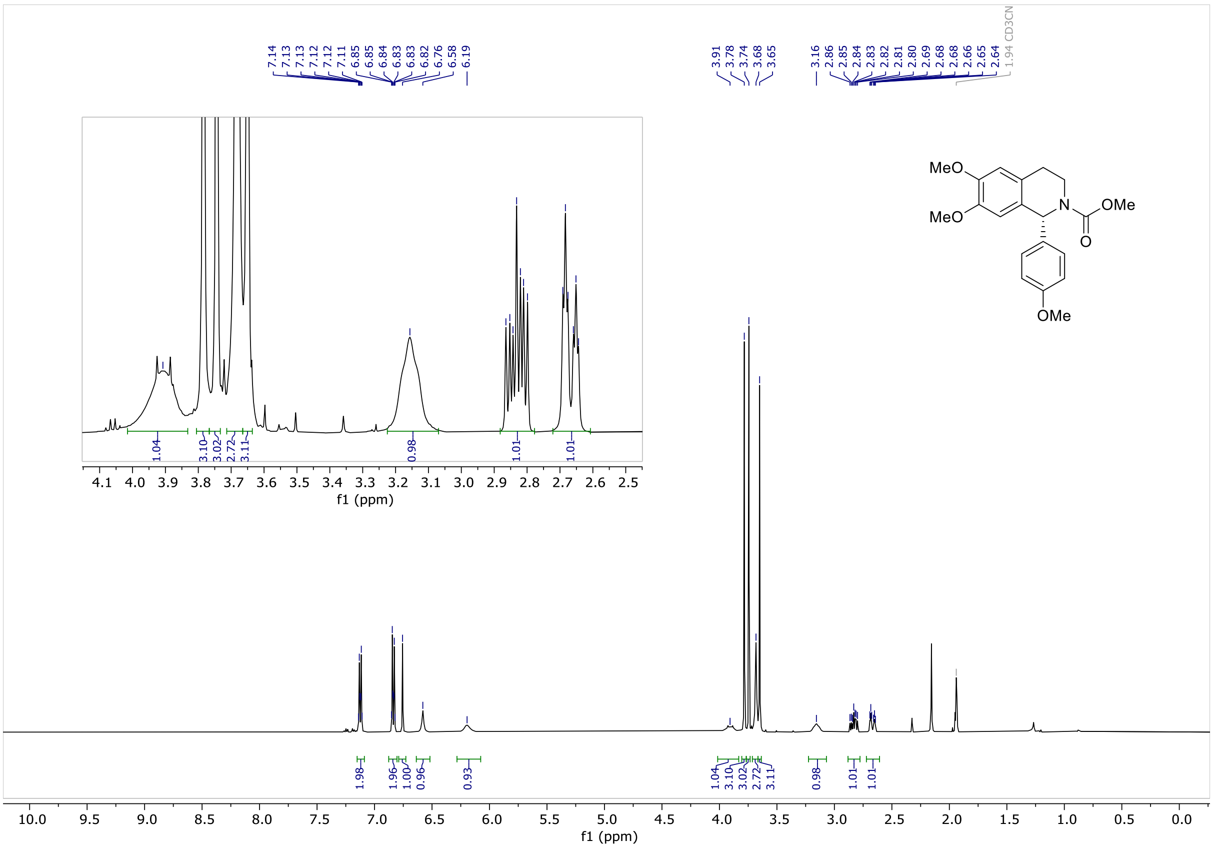

^{1}**H NMR** spectrum of compound **18h**.

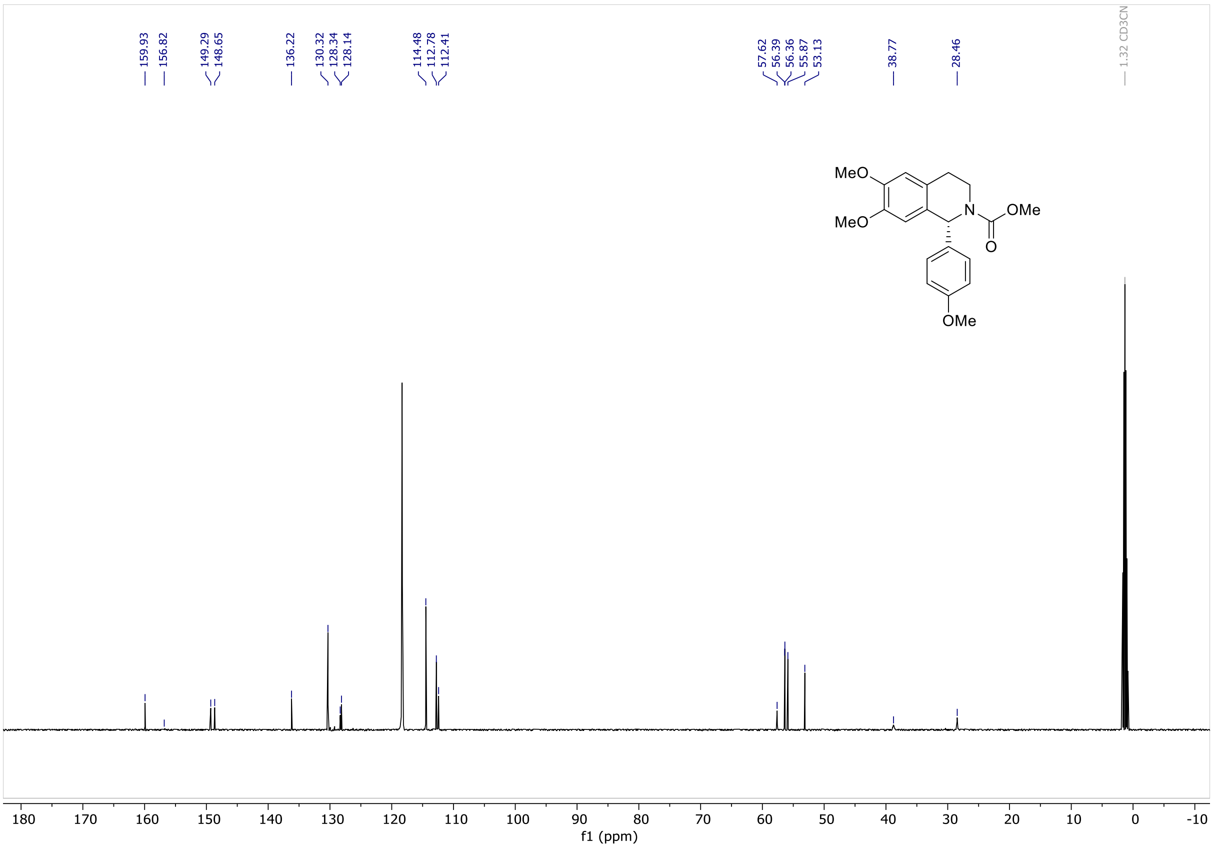

^{13}**C NMR** spectrum of compound **18h**.

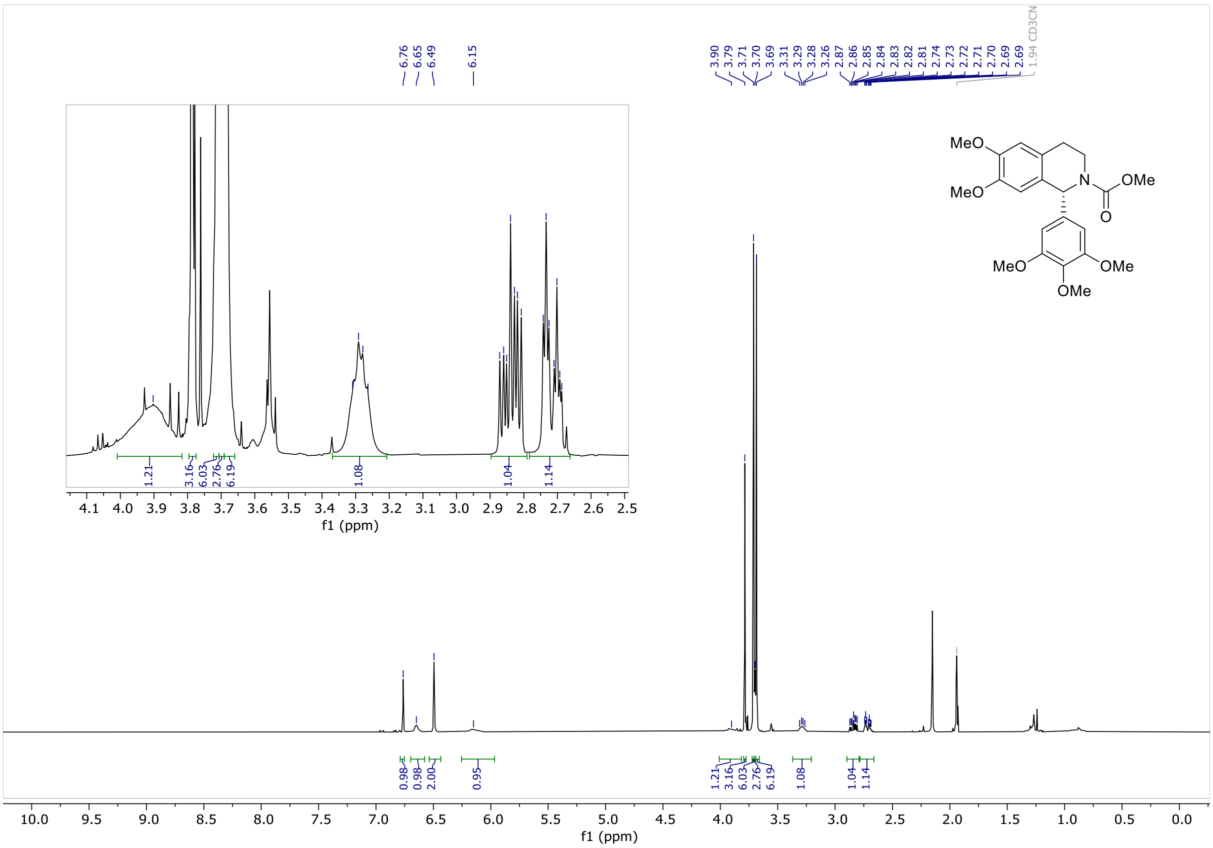

^{1}H NMR spectrum of compound **18i**.

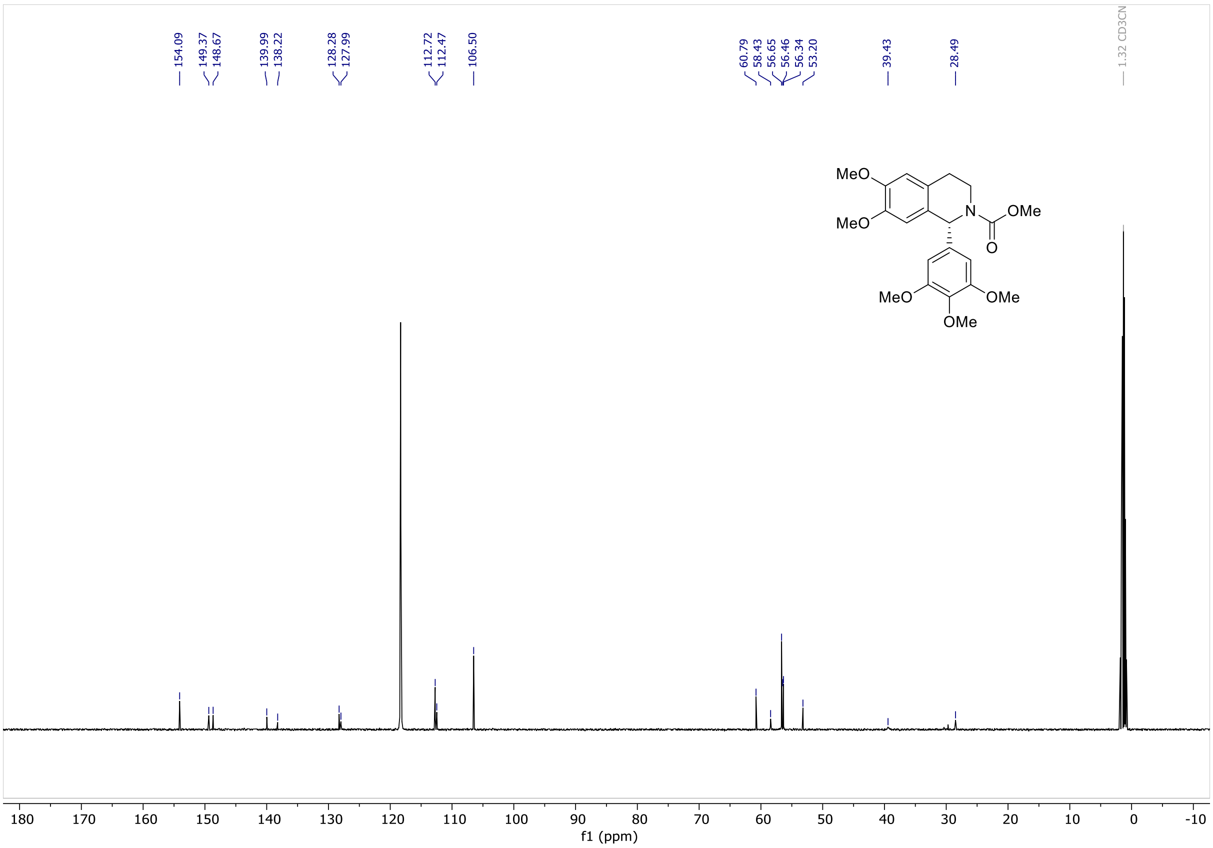

^{13}C NMR spectrum of compound **18i**.

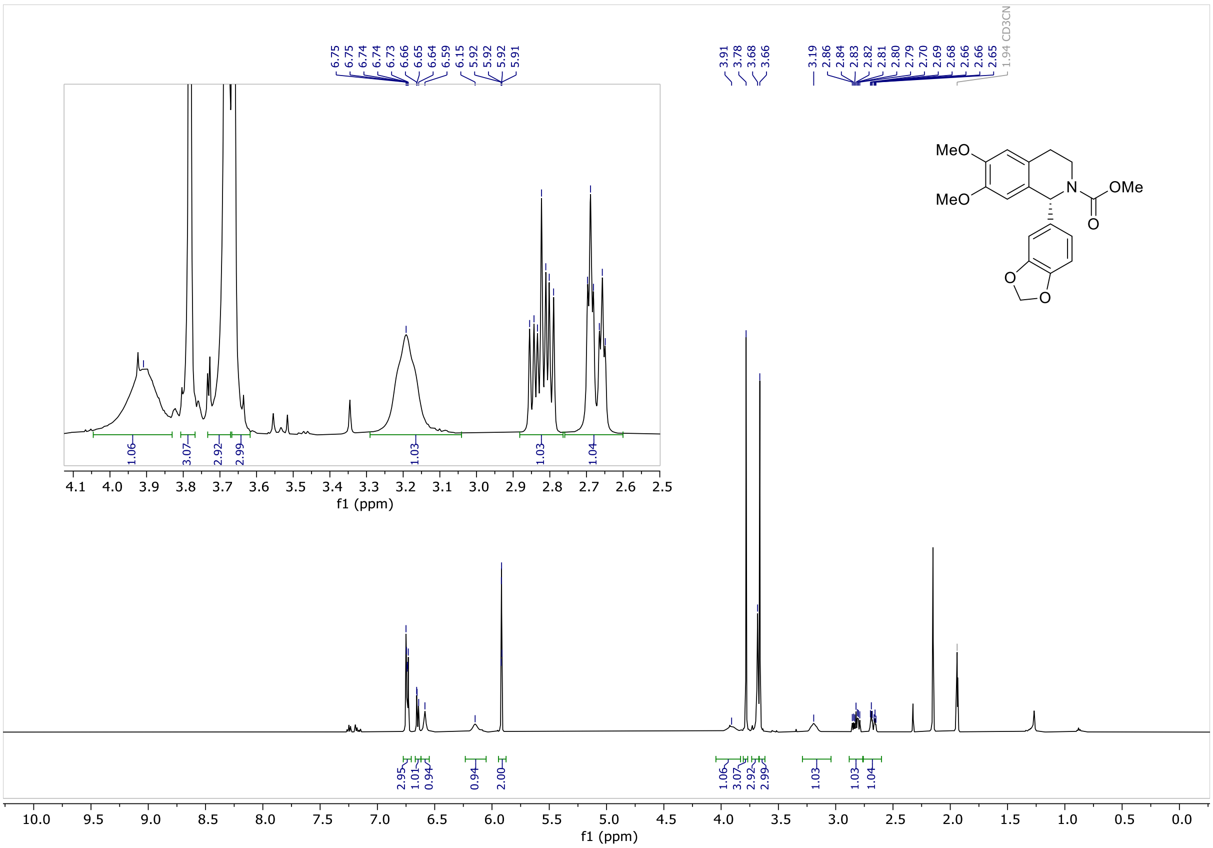

^{1}H NMR spectrum of compound **18j**.

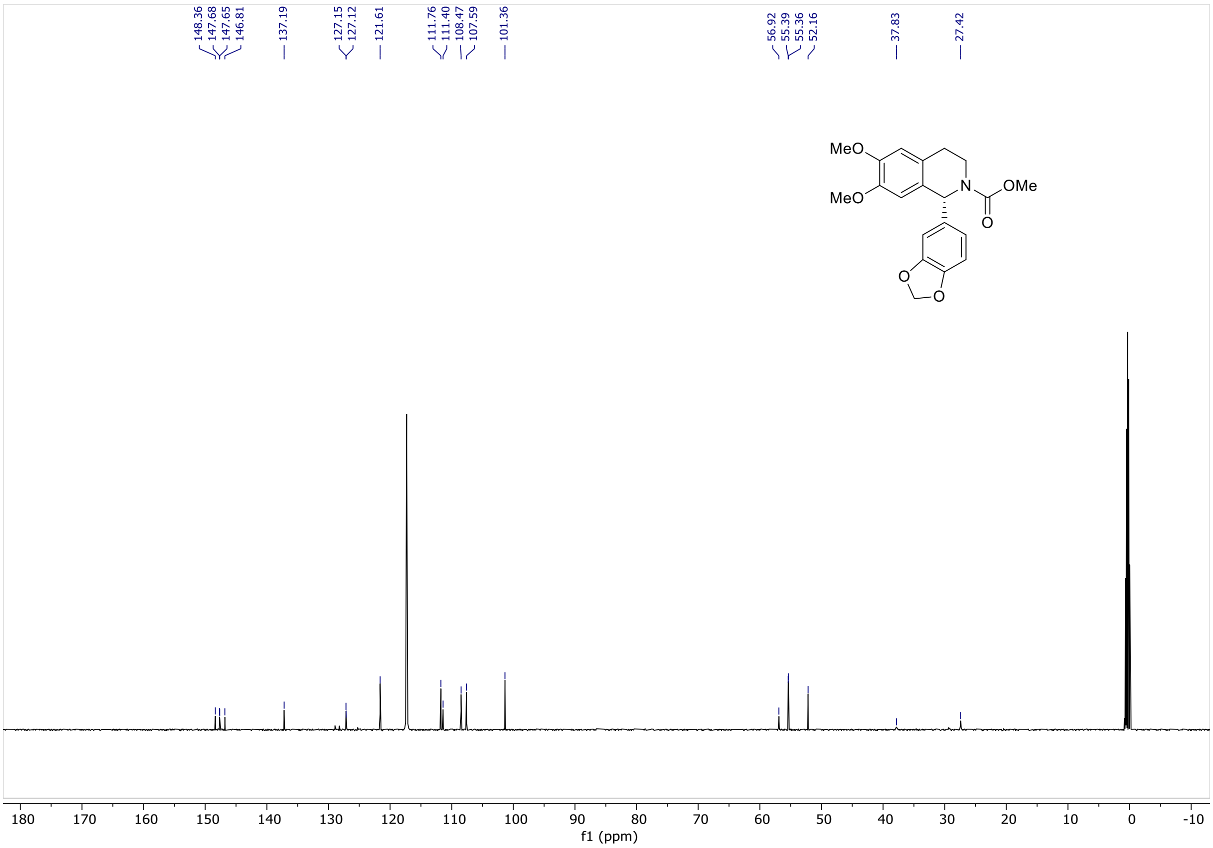

^{13}C NMR spectrum of compound **18j**.

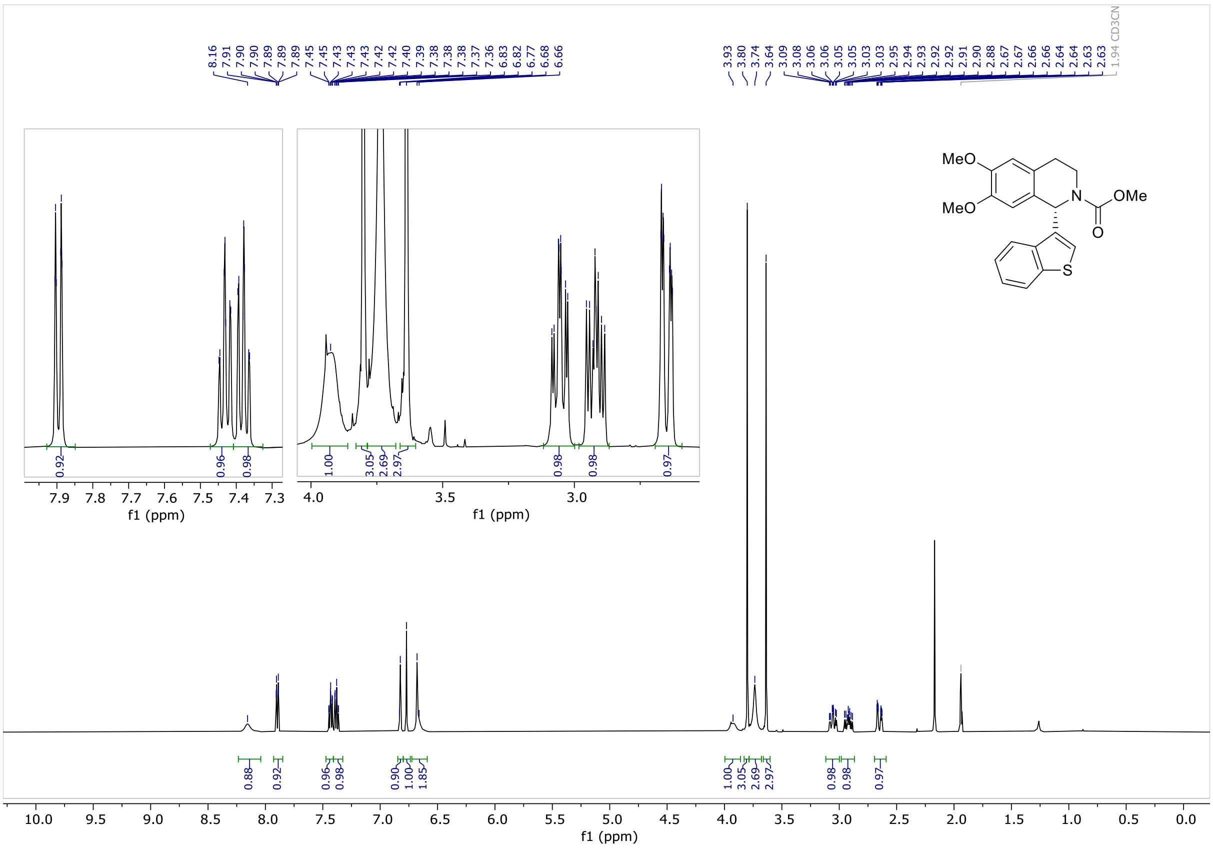

^{1}H NMR spectrum of compound **18k**.

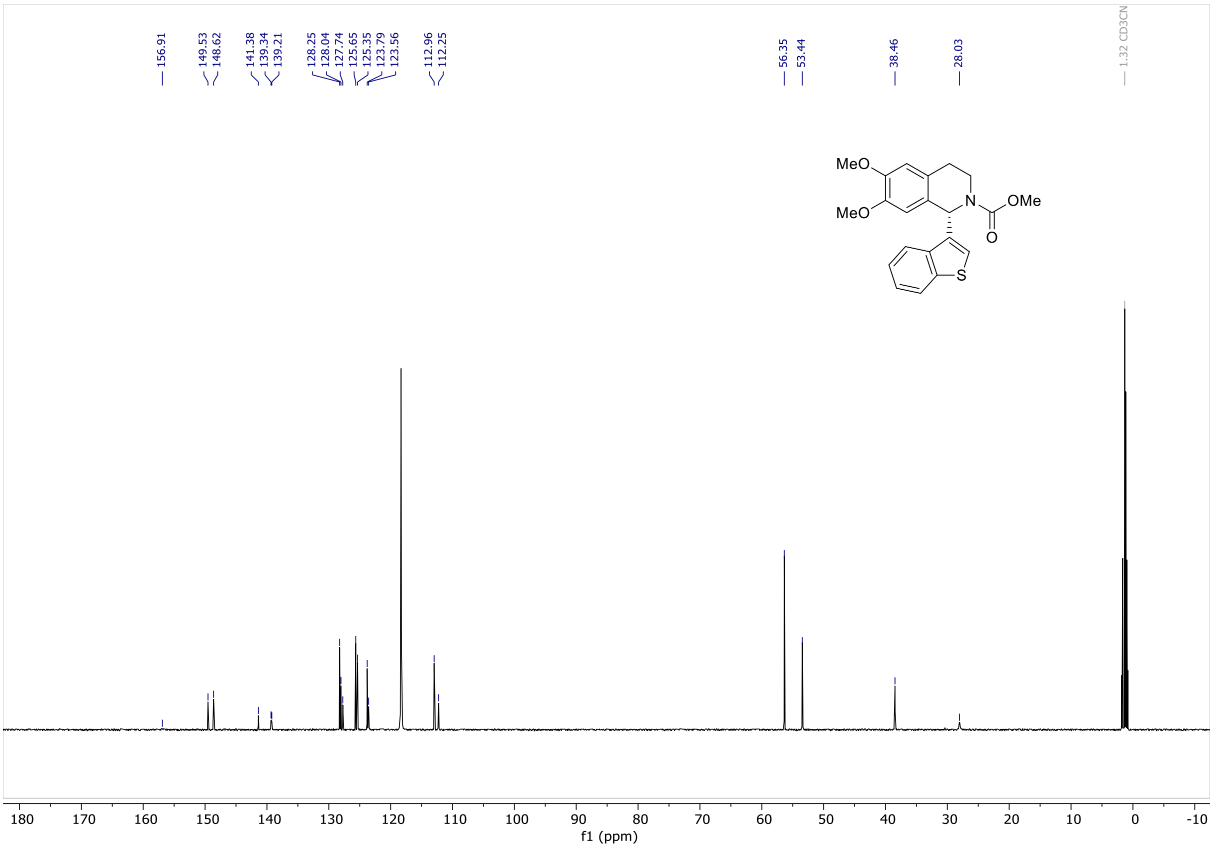

^{13}C NMR spectrum of compound **18k**.

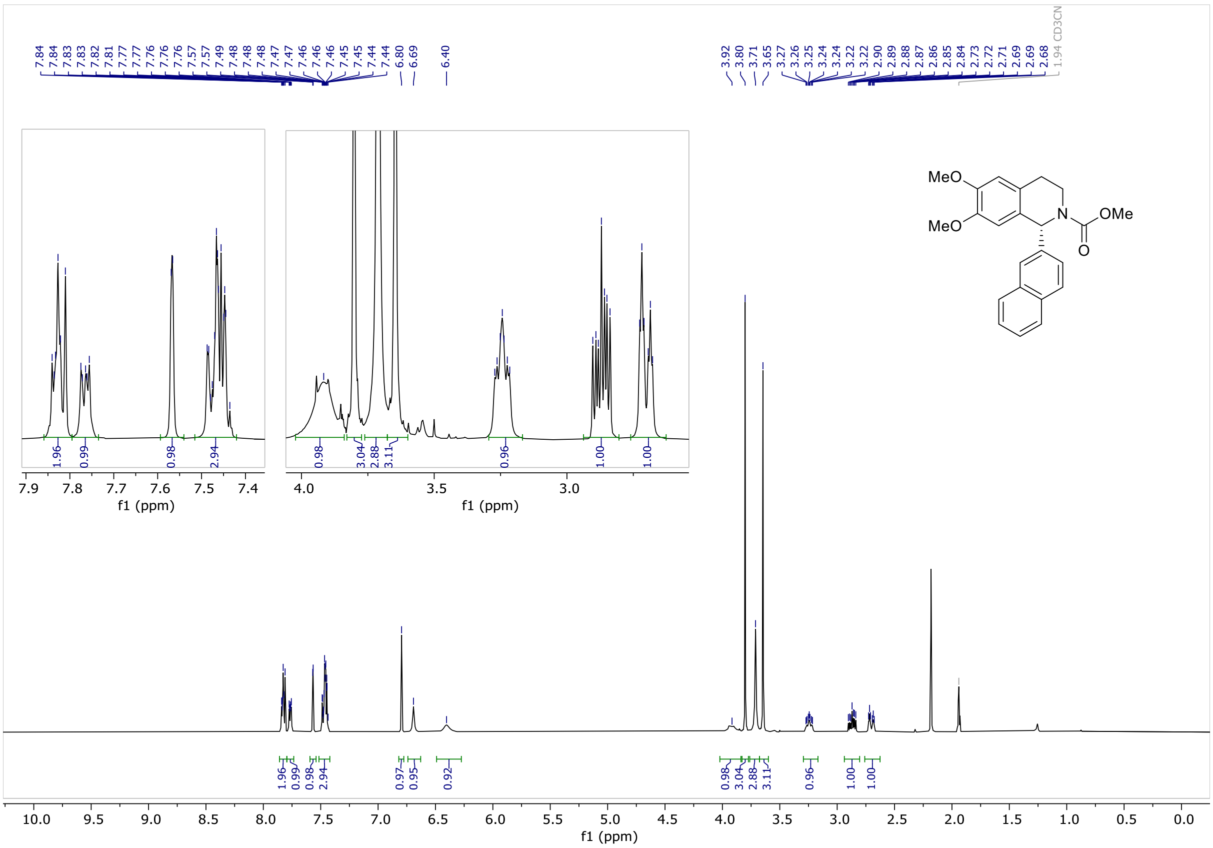

^{1}H NMR spectrum of compound **18l**.

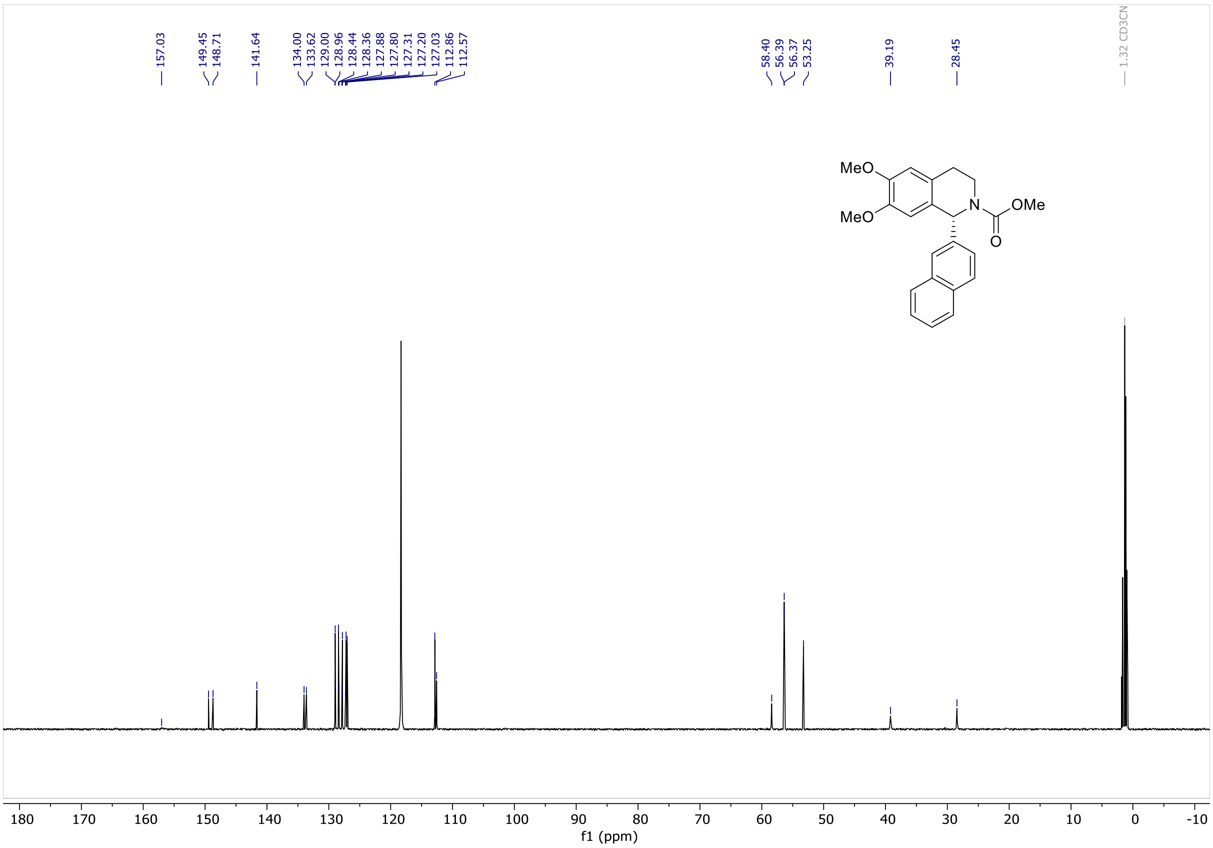

^{13}C NMR spectrum of compound **18l**.

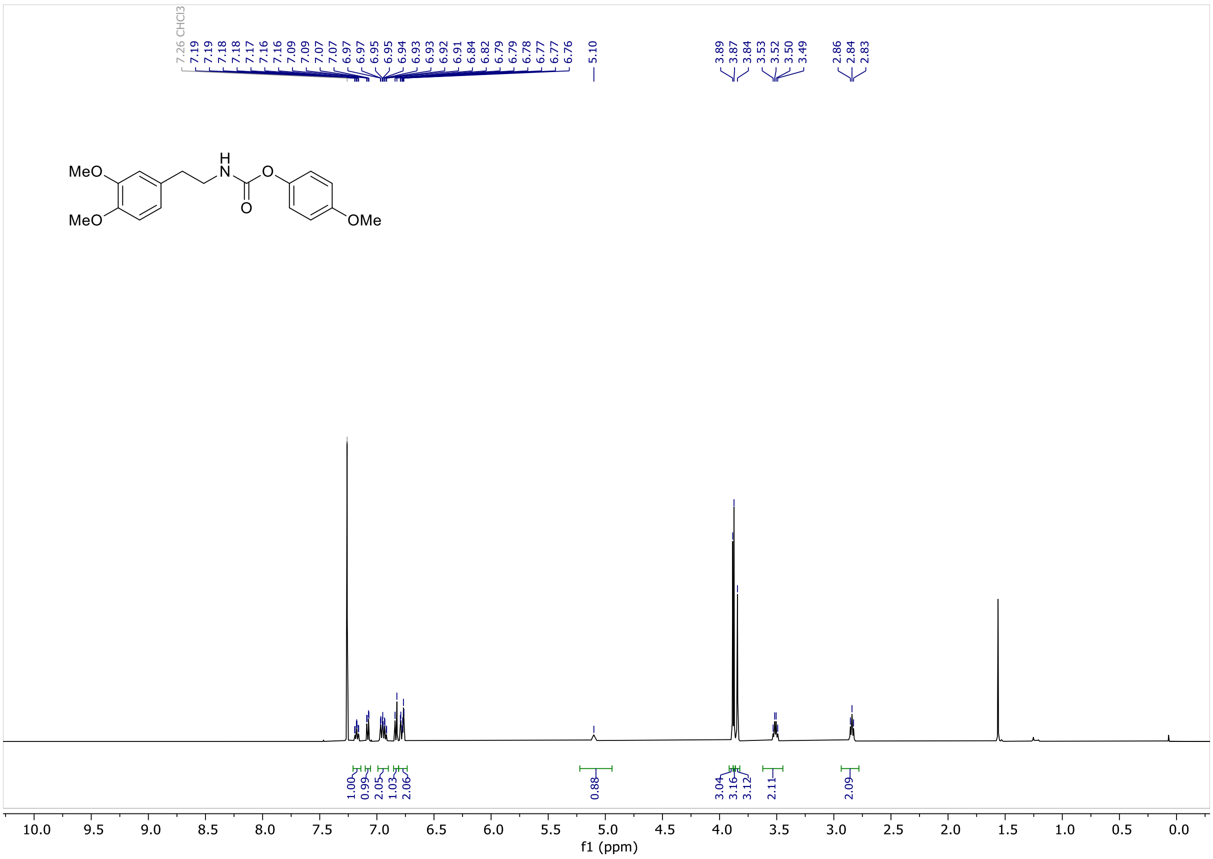

^{1}H NMR spectrum of compound **11a**.

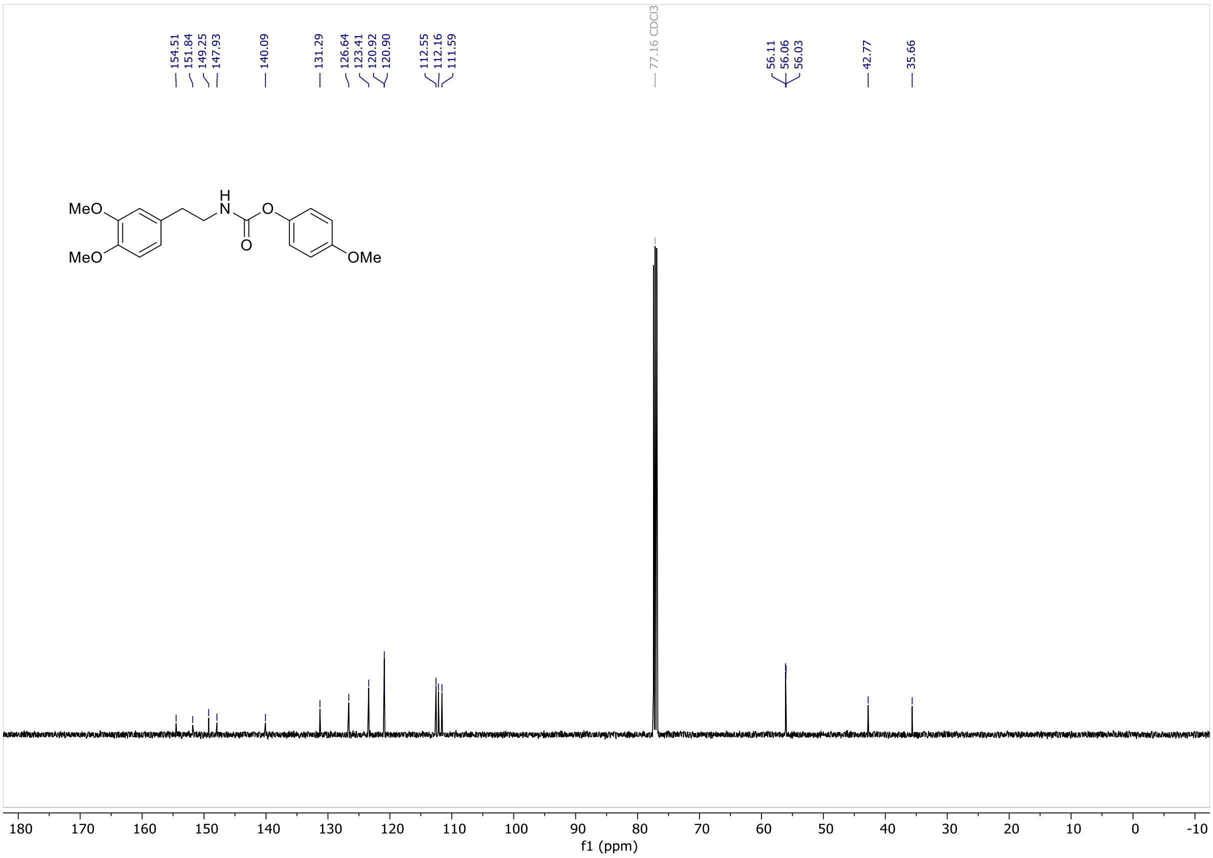

^{13}C NMR spectrum of compound **11a**.

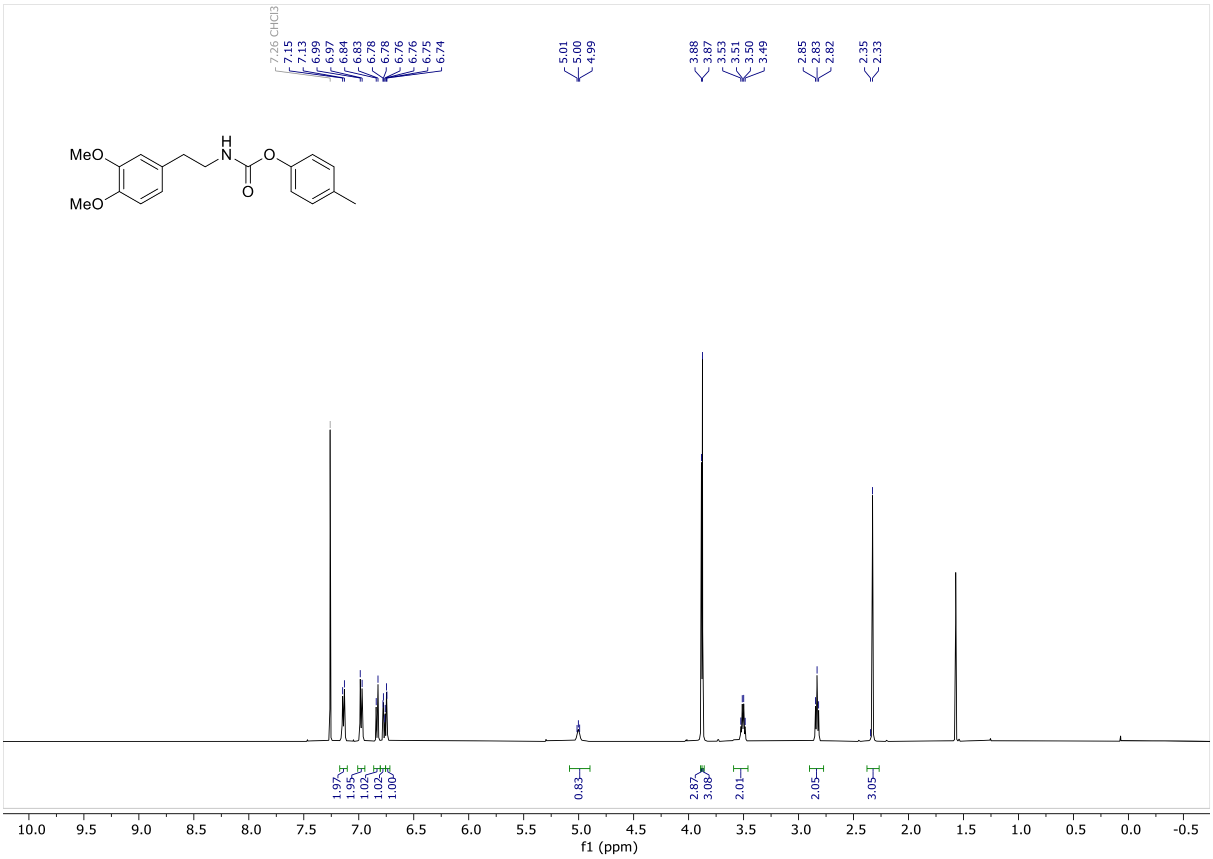

^{1}H NMR spectrum of compound **11b**.

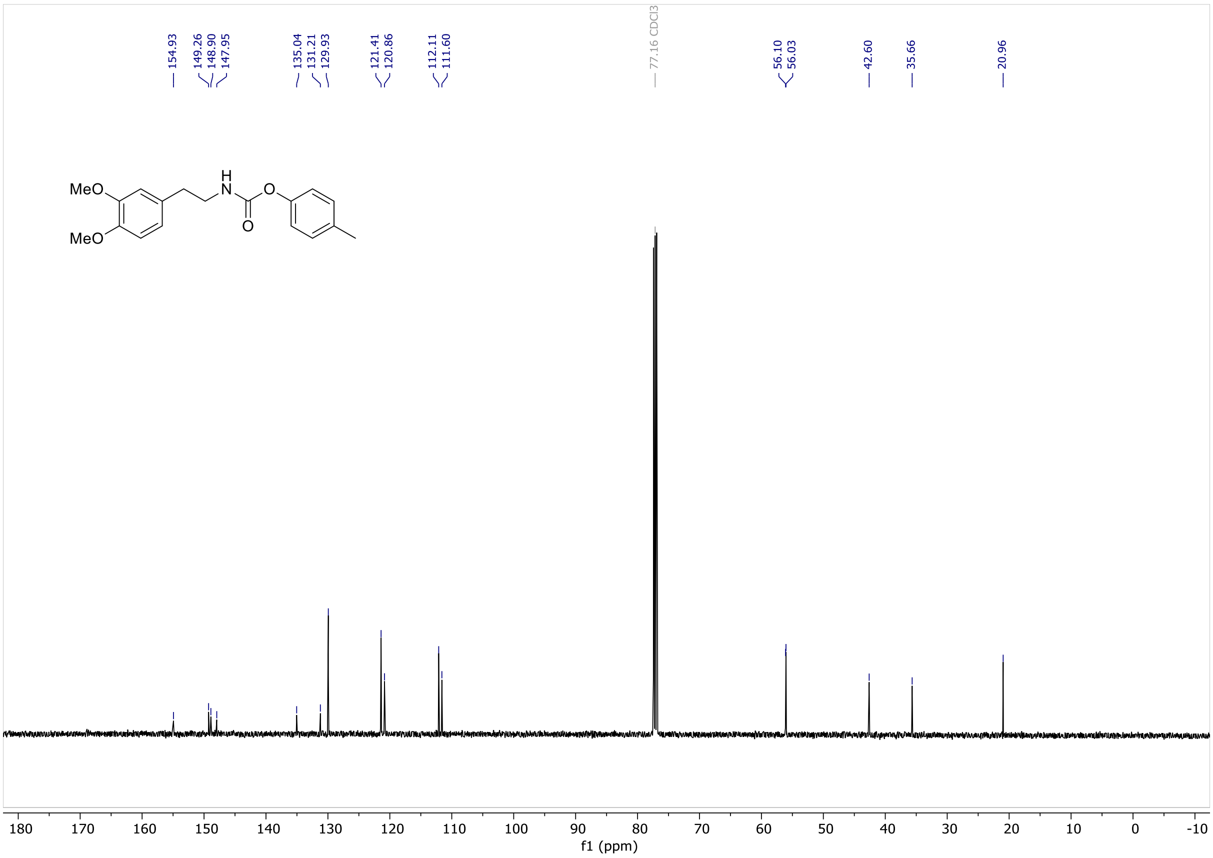

^{13}C NMR spectrum of compound **11b**.

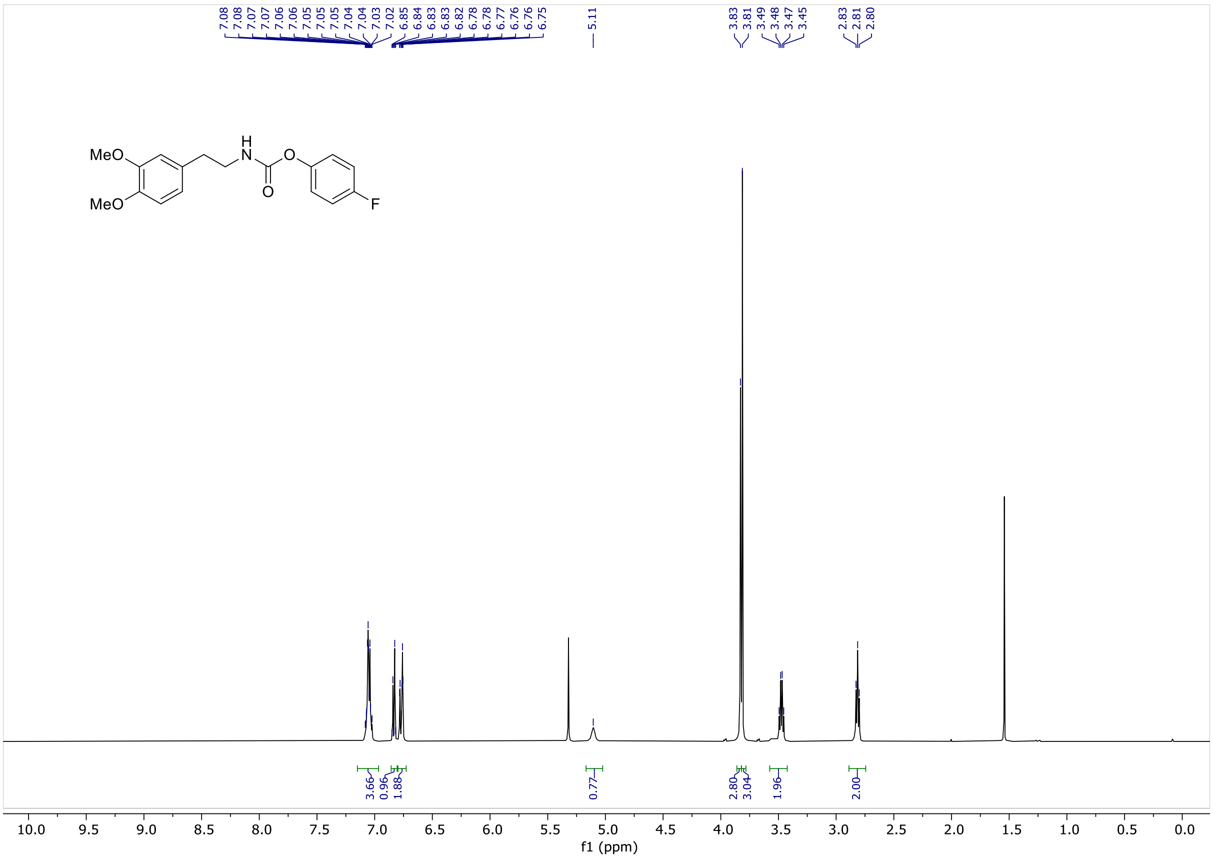

^{1}H NMR spectrum of compound **11d**.

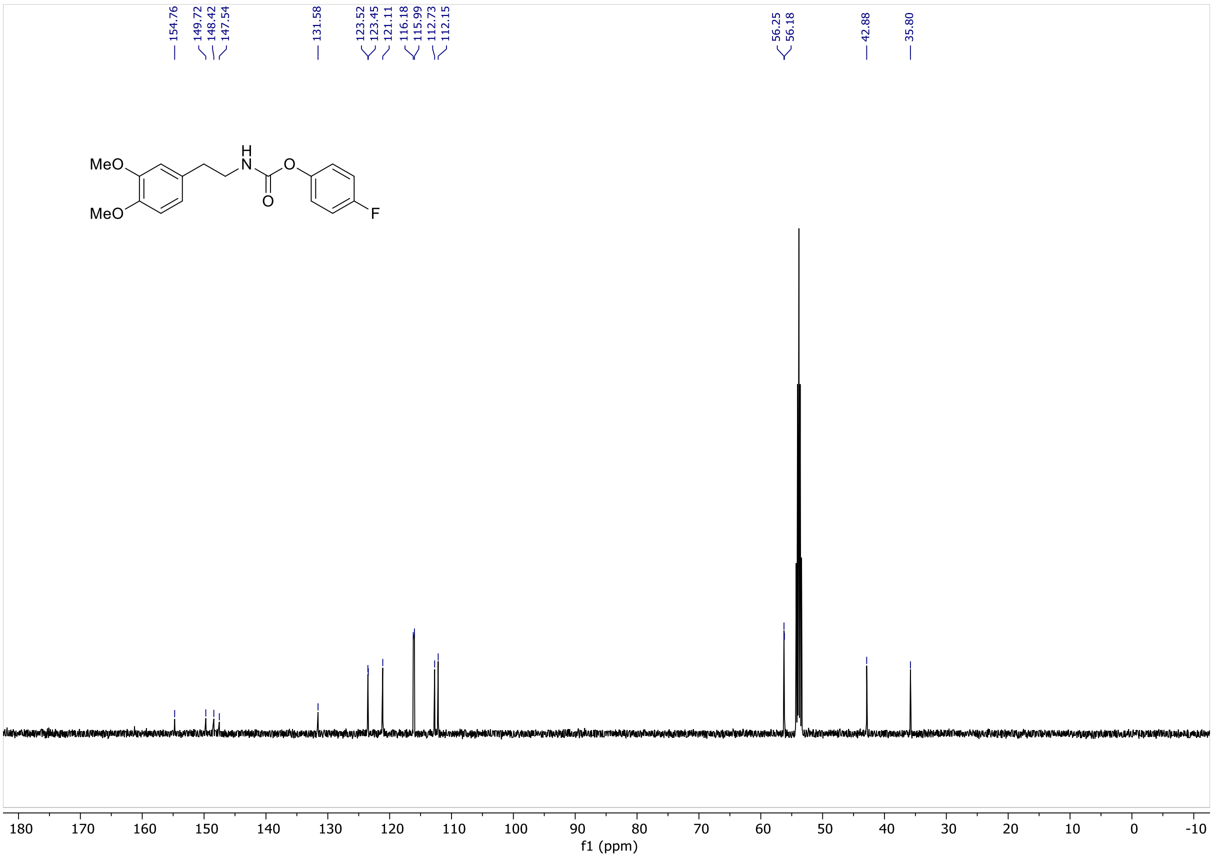

^{13}C NMR spectrum of compound **11d**.

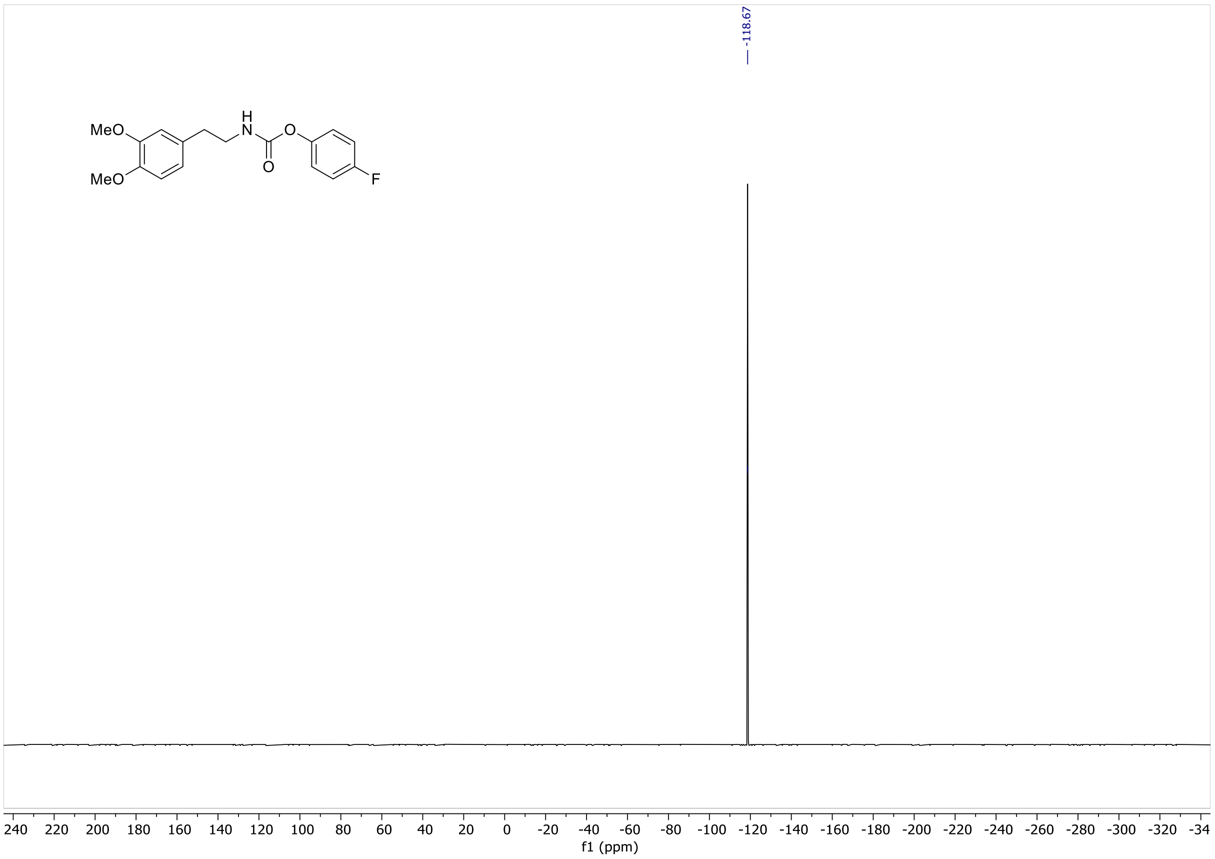

^{19}**F NMR** spectrum of compound **11d**.

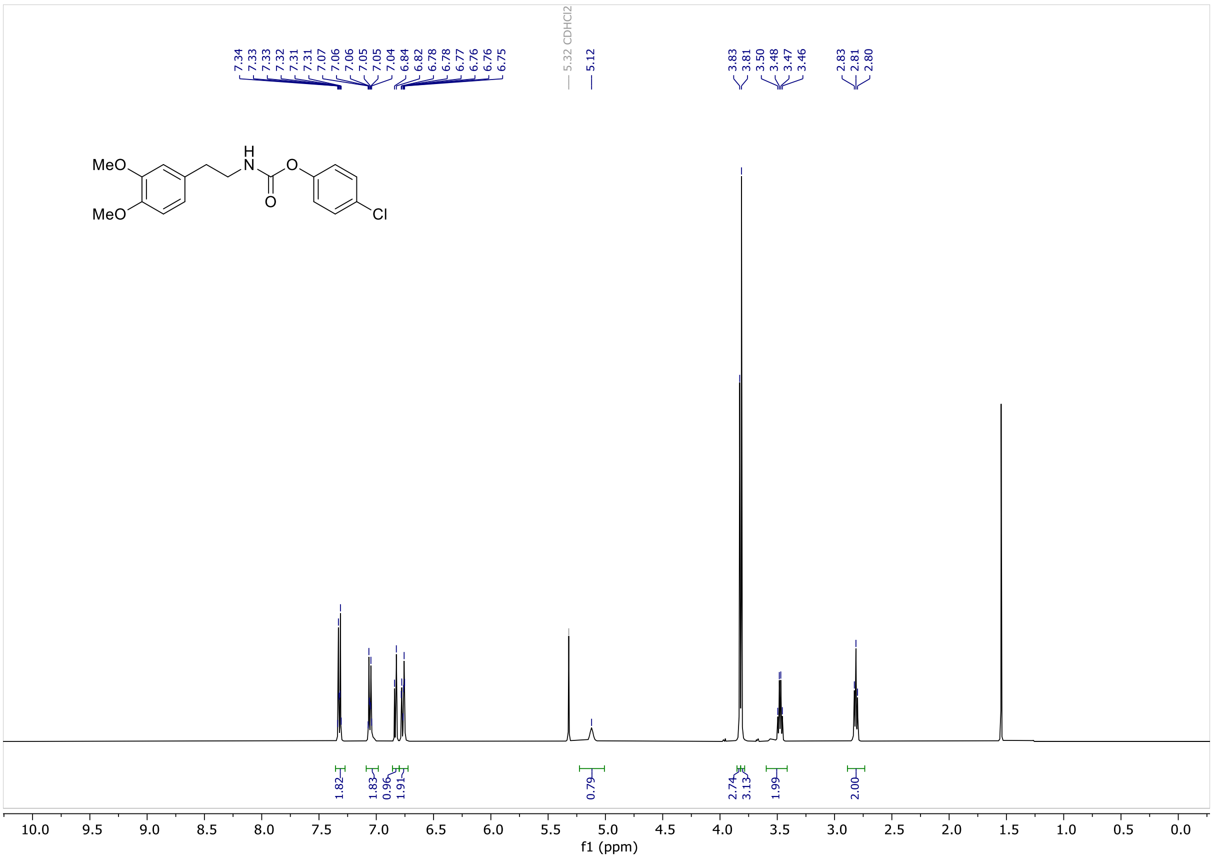

^{1}H NMR spectrum of compound **11e**.

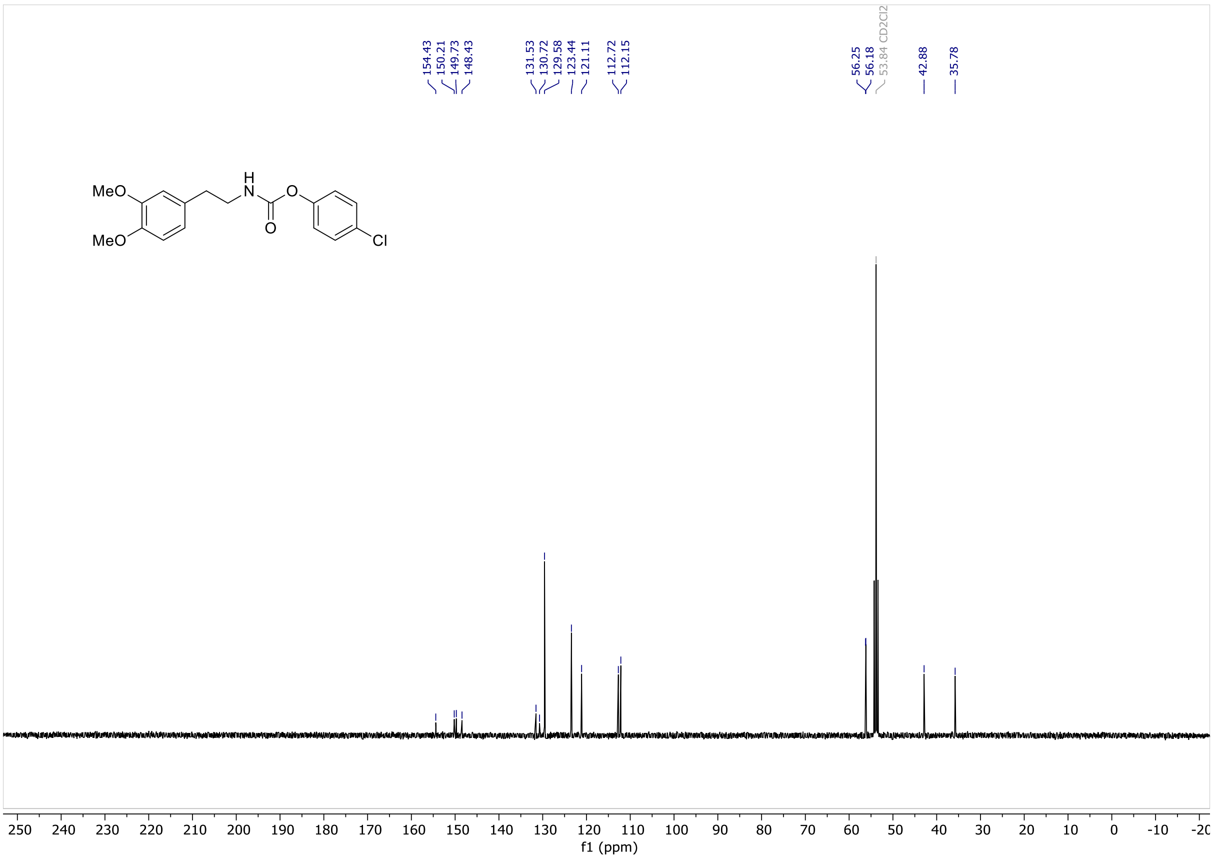

^{13}C NMR spectrum of compound **11e**.

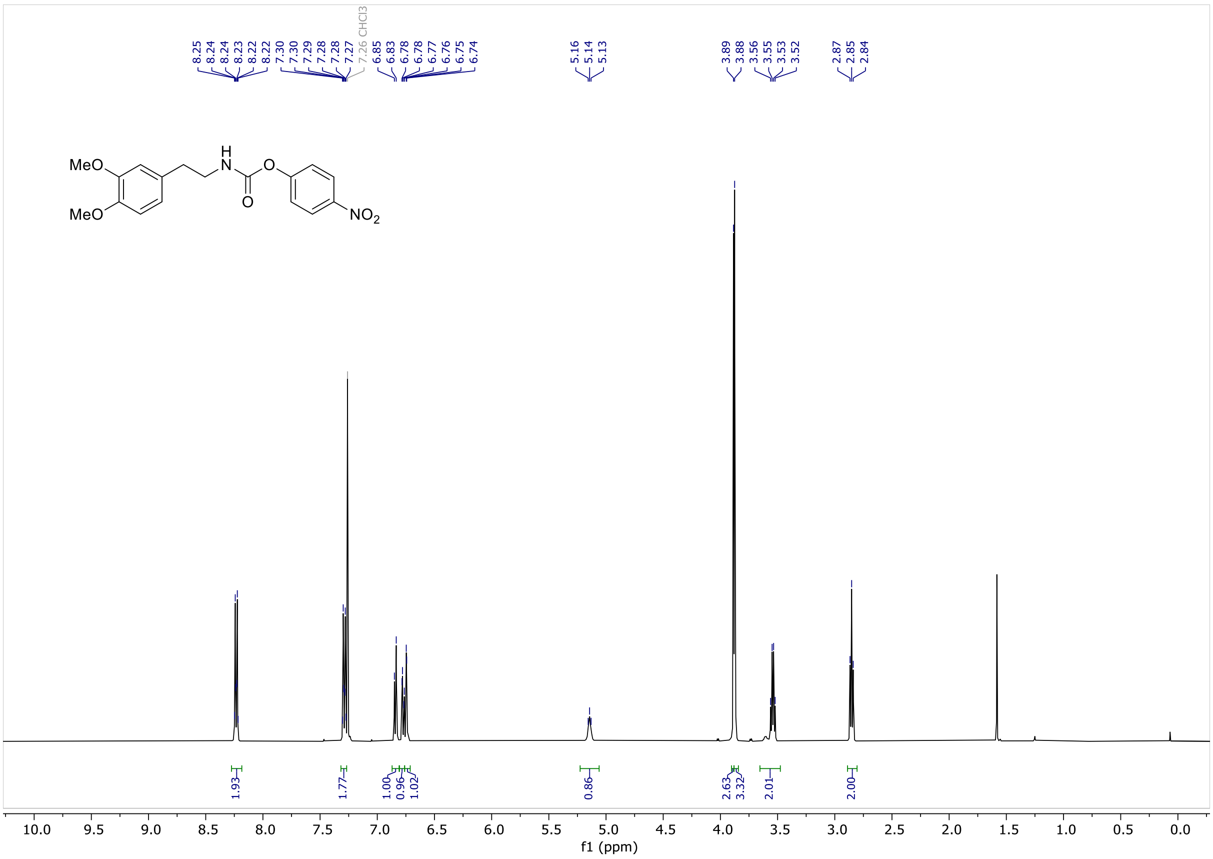

^{1}H NMR spectrum of compound **11f**.

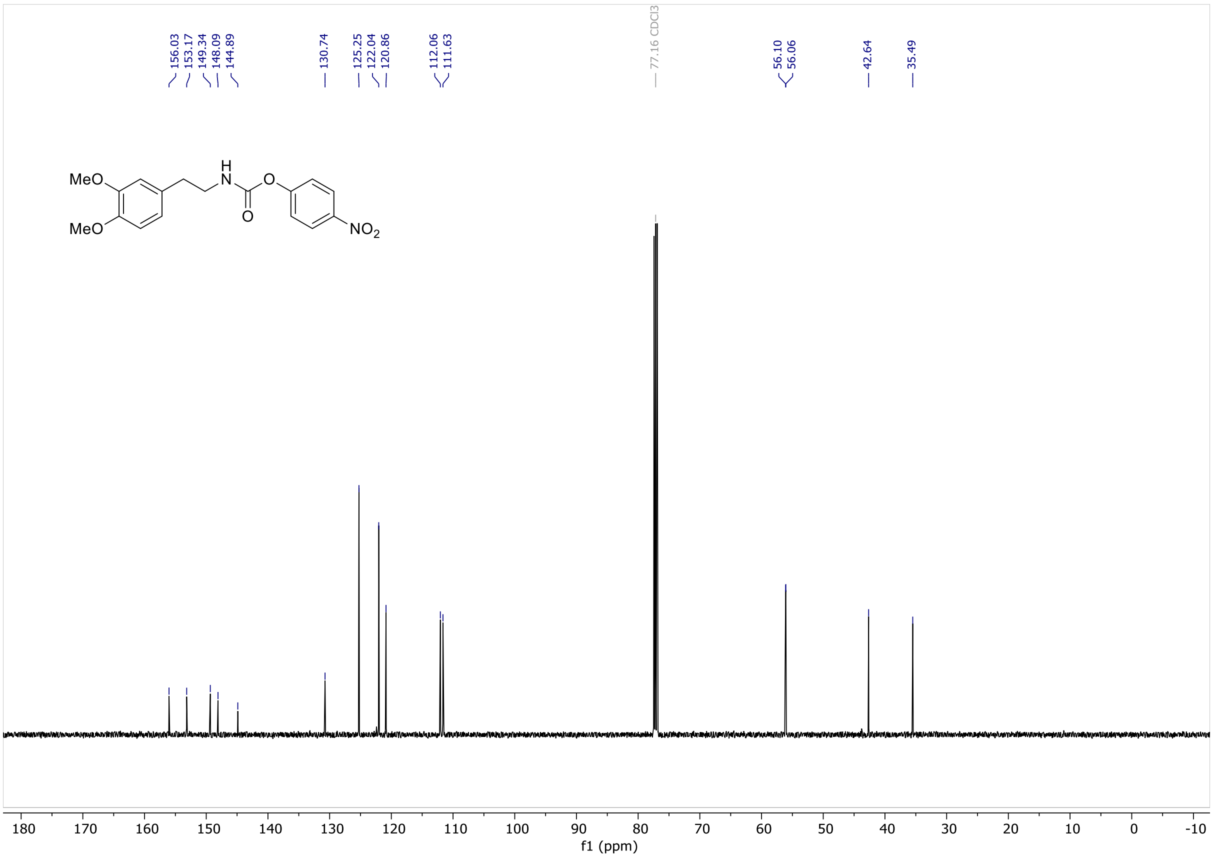

^{13}C NMR spectrum of compound **11f**.

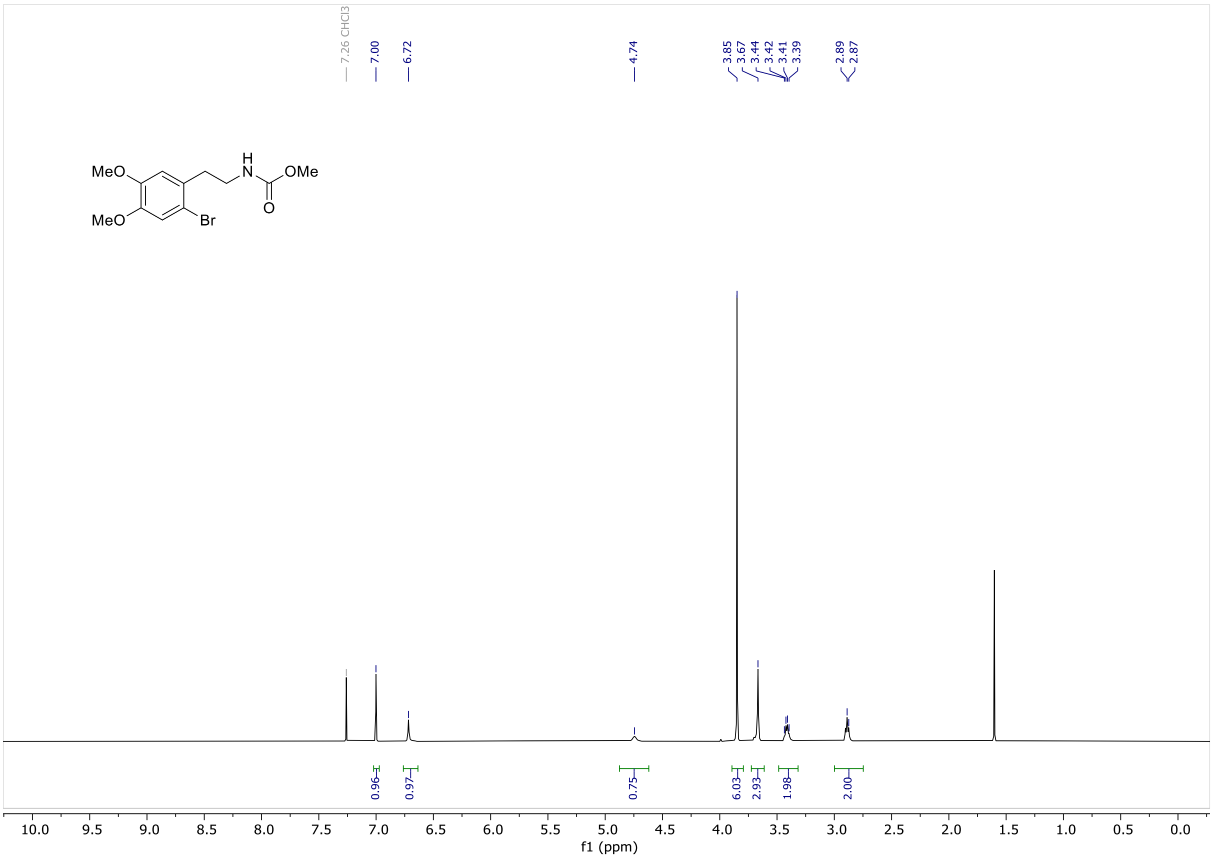

^{1}H NMR spectrum of compound **S2**.

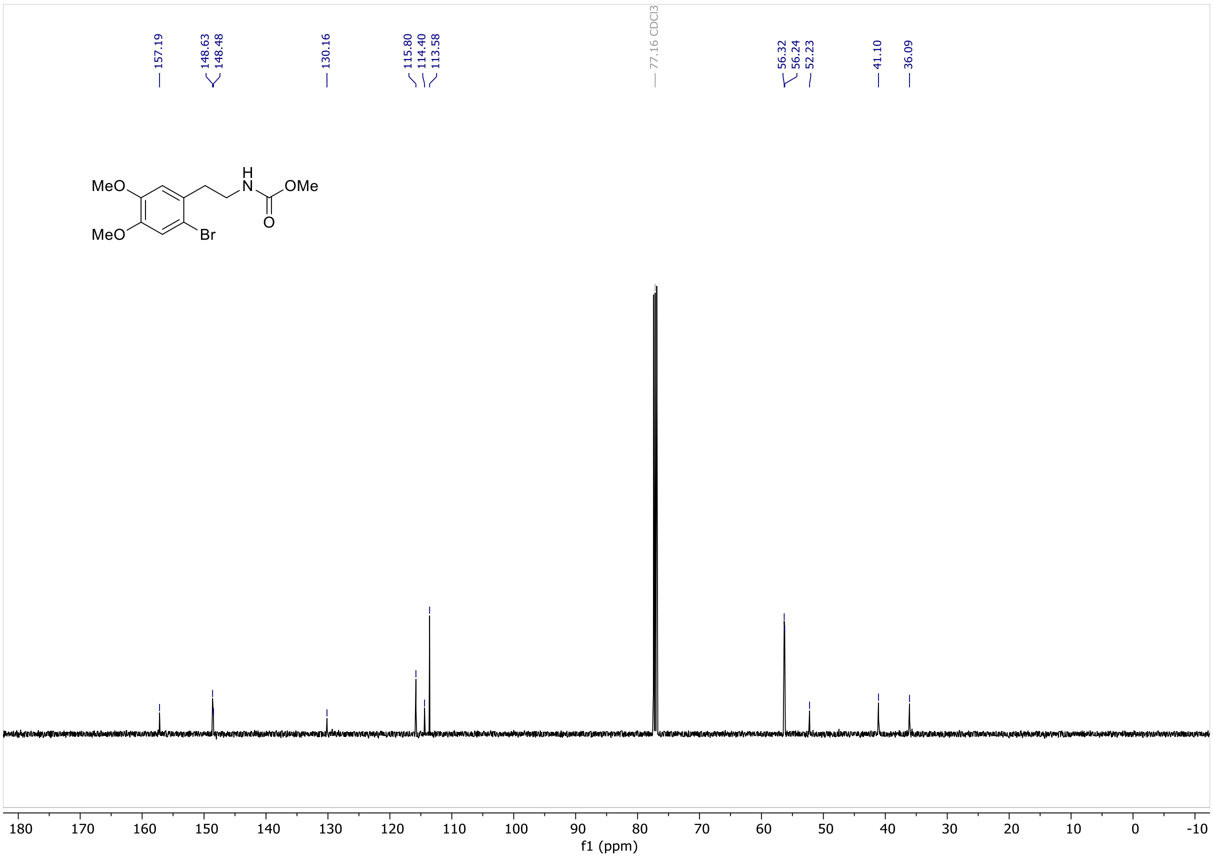

^{13}C NMR spectrum of compound **S2**.

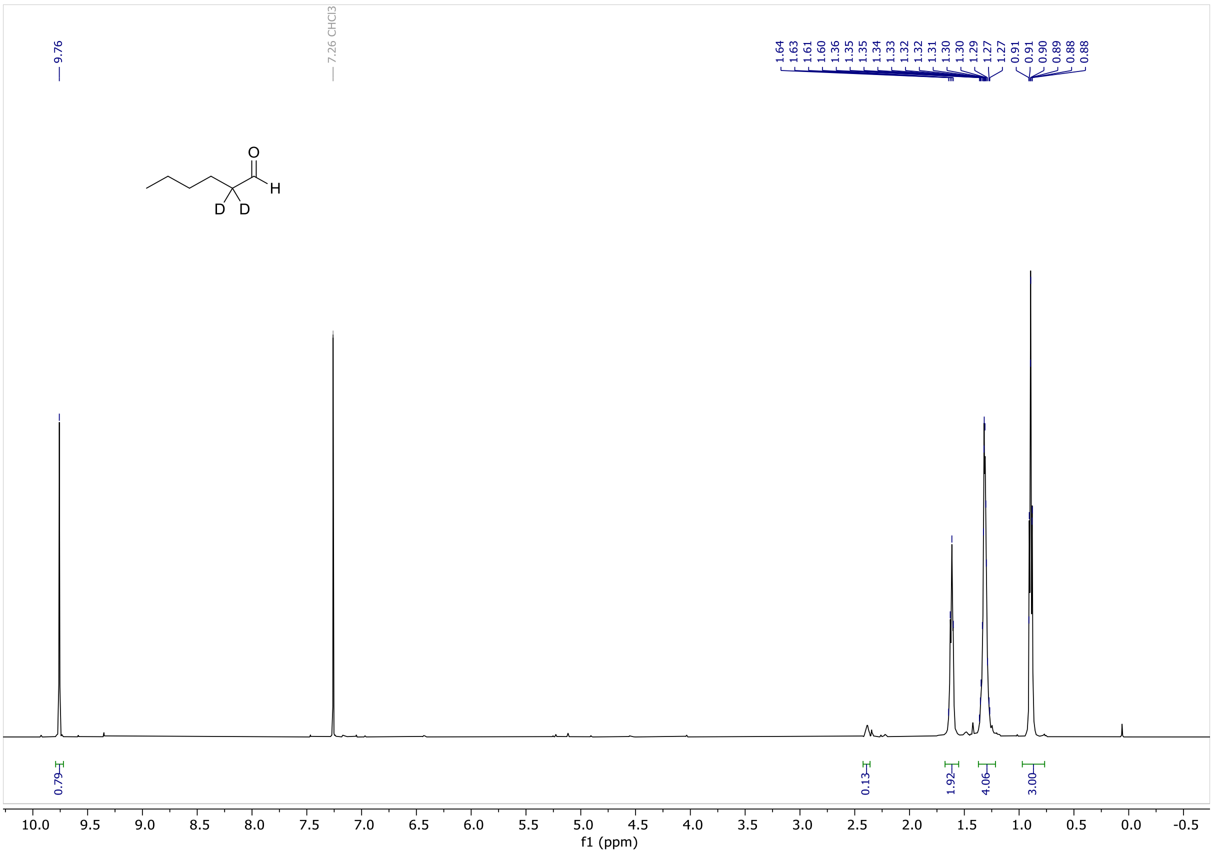

^{1}H NMR spectrum of compound **14-D$_2$**.

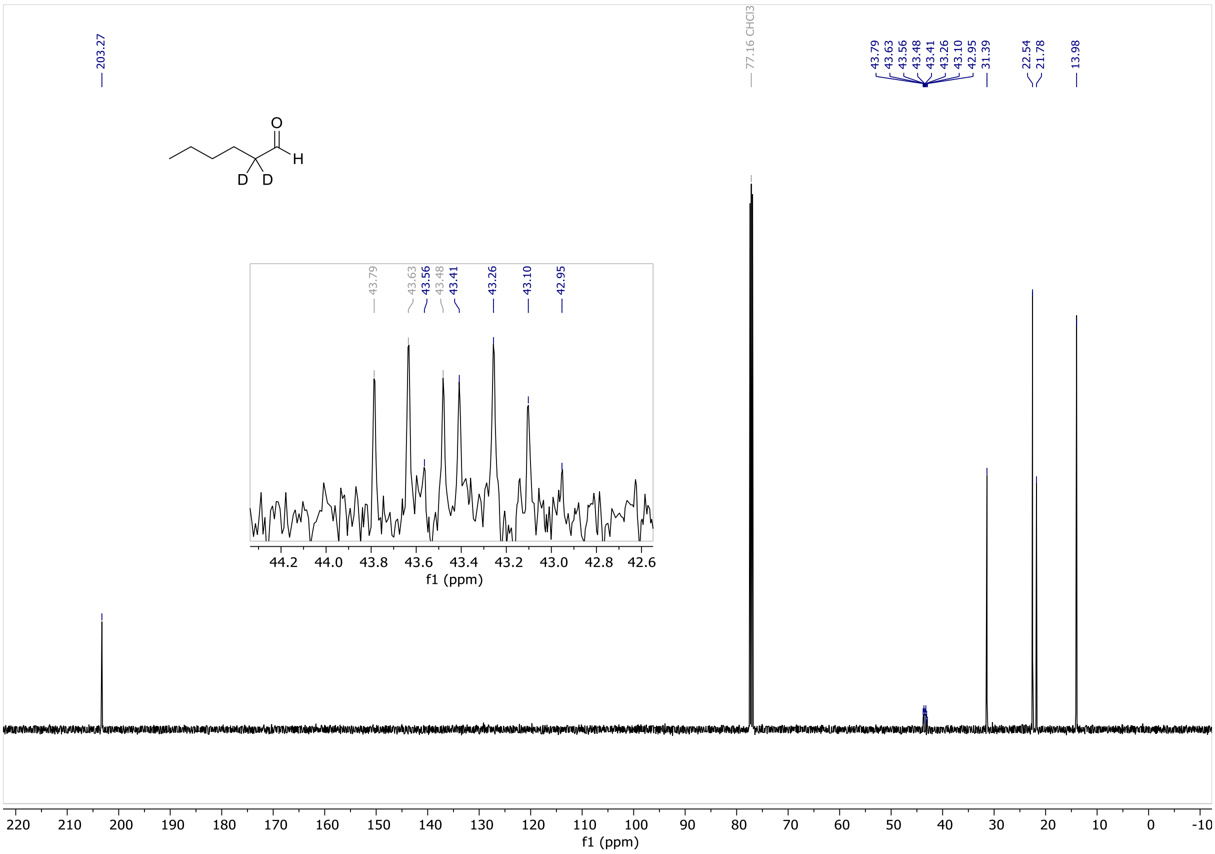

^{13}C NMR spectrum of compound **14-D$_2$**. *Note: Remaining triplet of mono-deuterated compound at 43.63 ppm.*

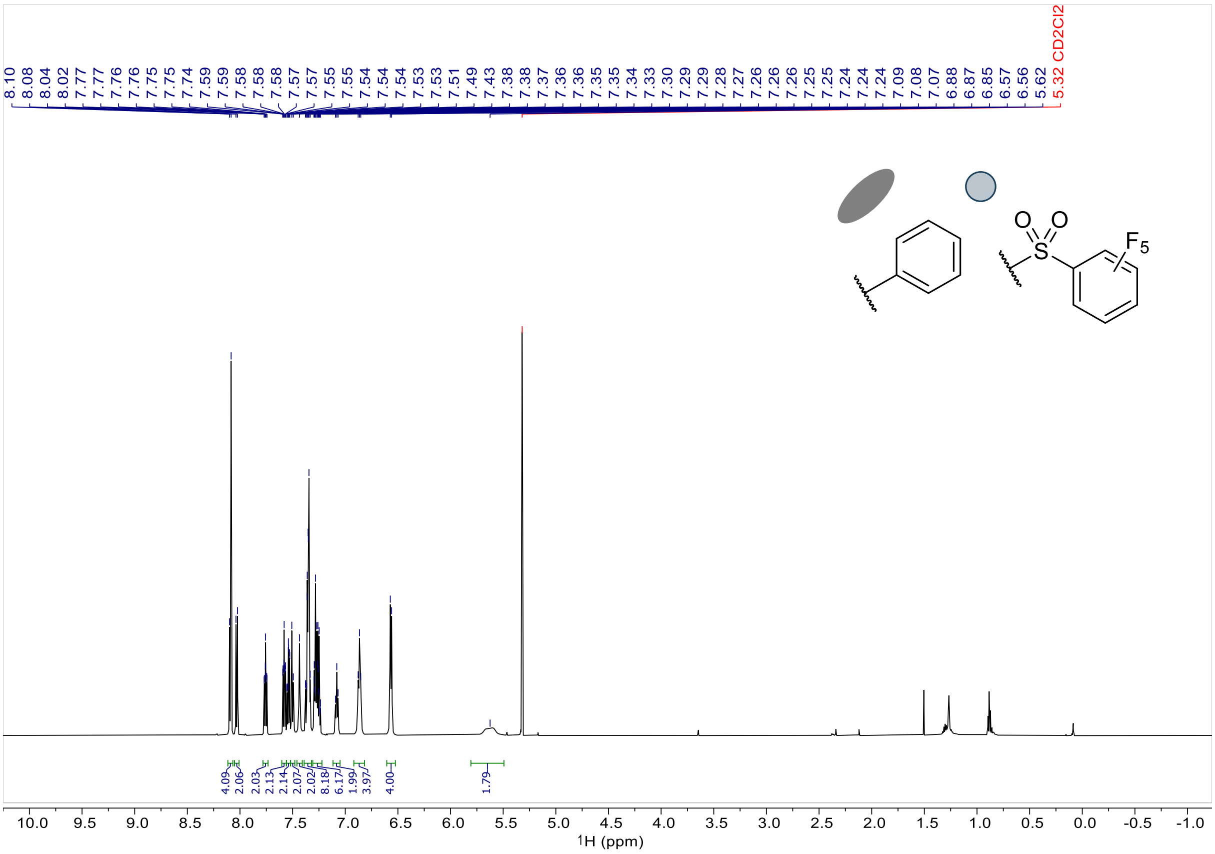

¹H NMR spectrum of IDPi **1b**.

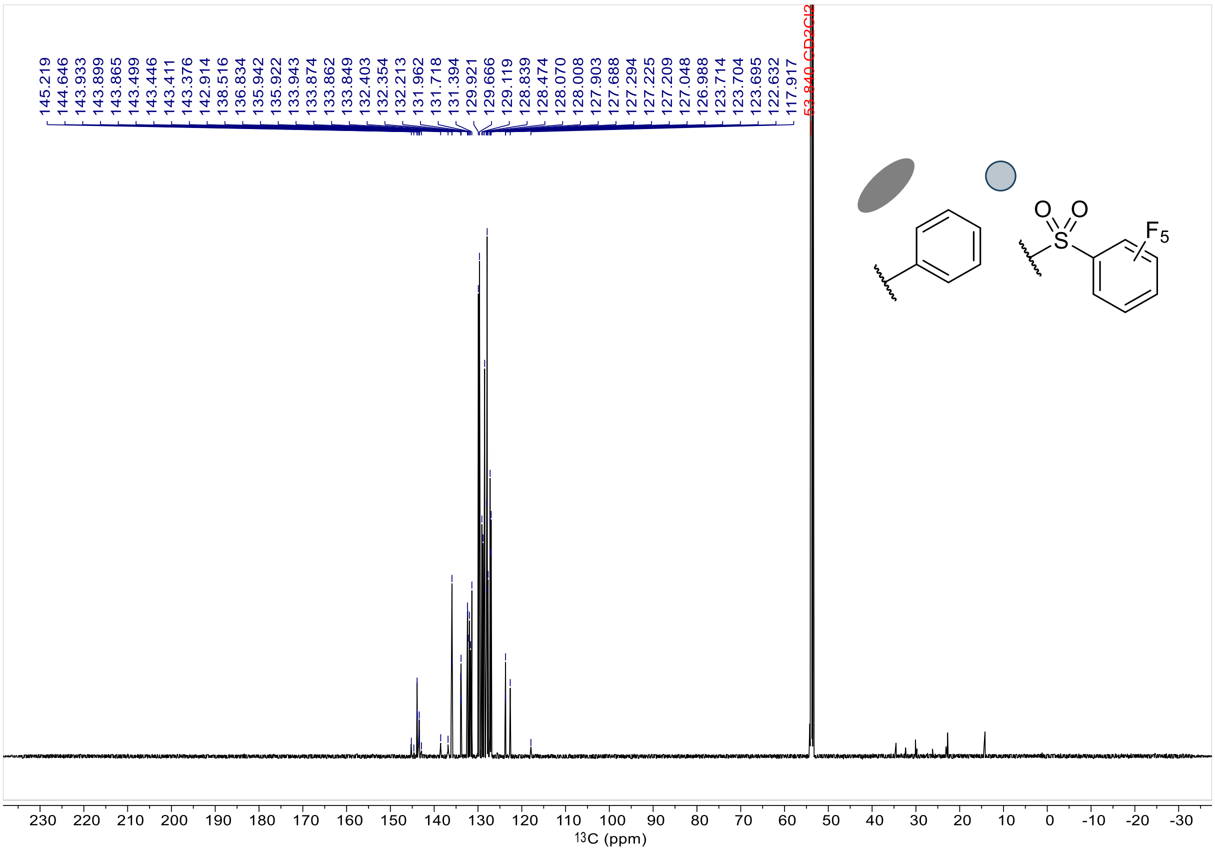

¹³C NMR spectrum of IDPi **1b**.

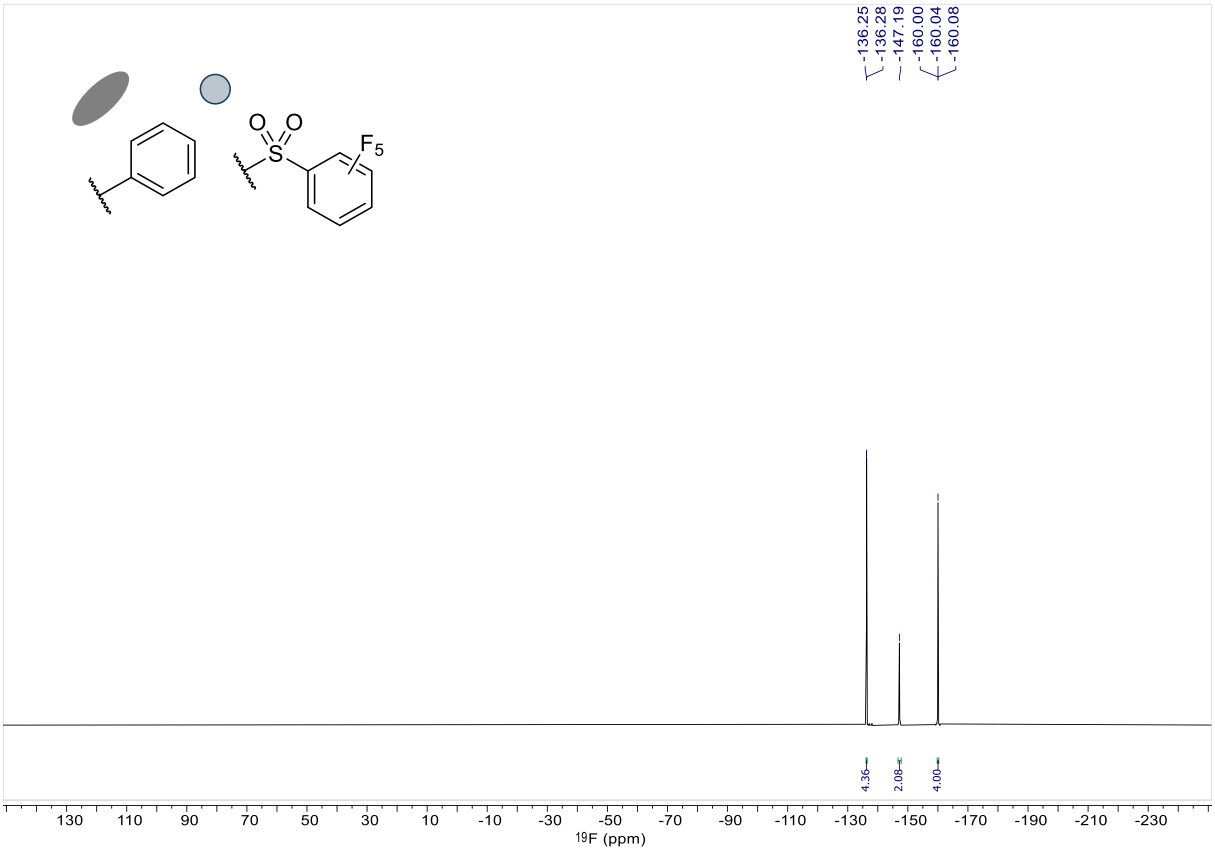

^{19}F NMR spectrum of IDPi **1b**.

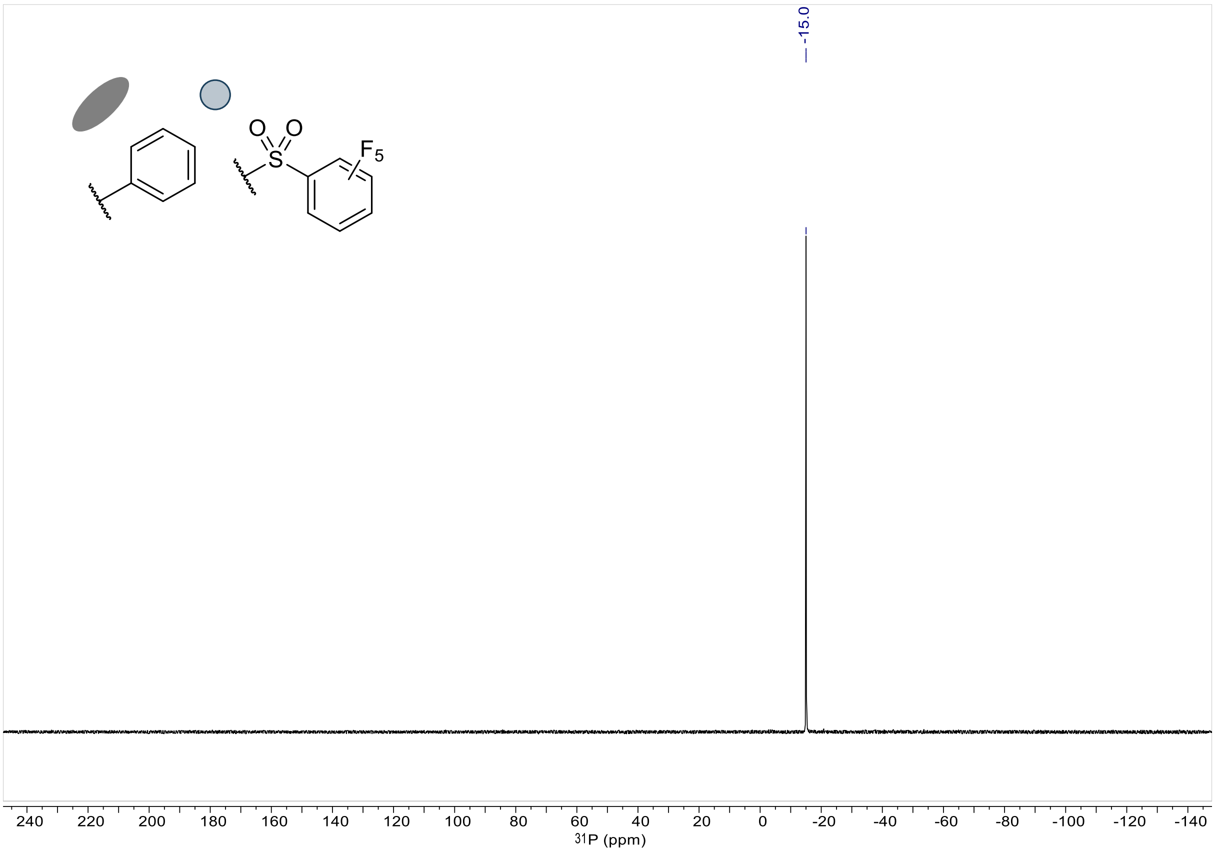

^{31}P NMR spectrum of IDPi **1b**.

10. HPLC Traces

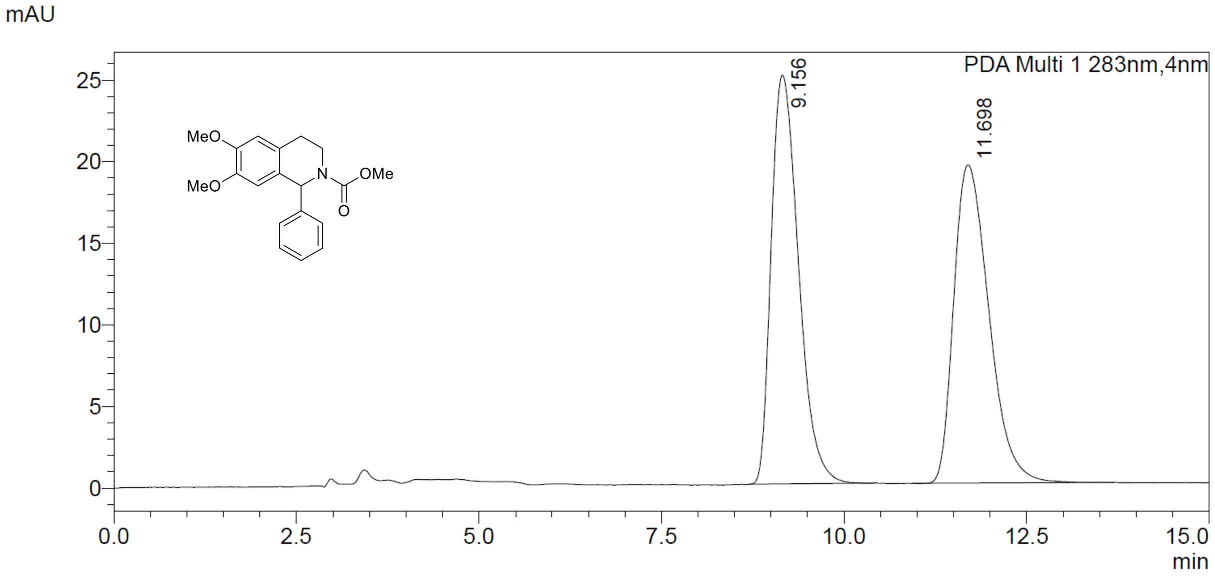

IA-3, *n*-heptane/*i*-PrOH 80:20, 298 K, 283 nm		
peak #	t_R / min	area / %
1	9.156	49.932
2	11.698	50.068

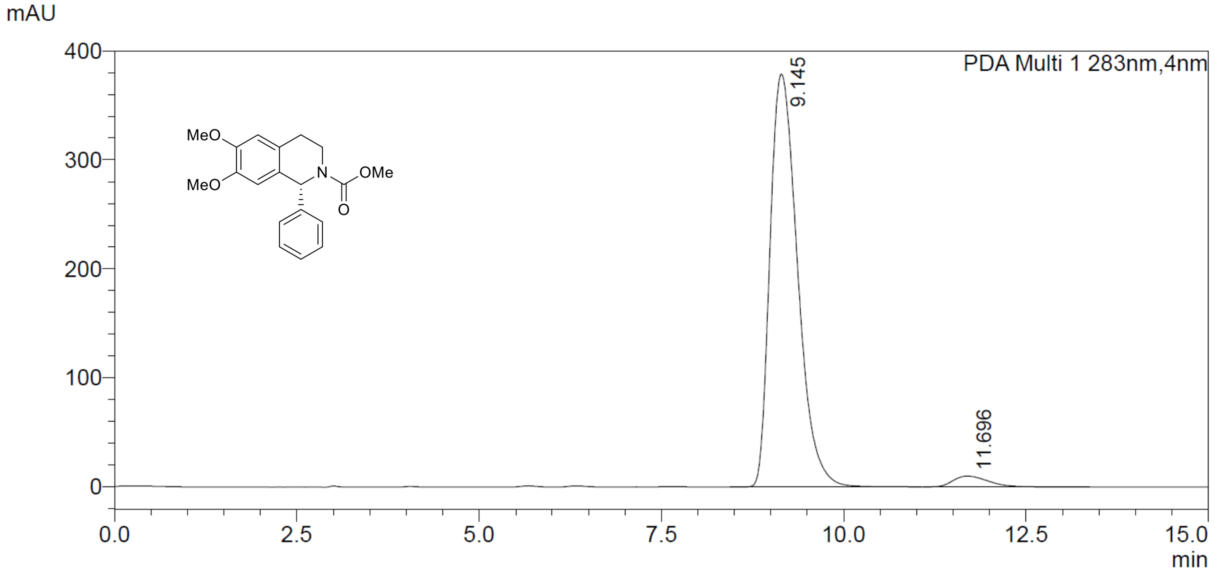

IA-3, *n*-heptane/i-PrOH 80:20, 298 K, 283 nm		
peak #	t_R / min	area / %
1	9.145	96.753
2	11.696	3.247

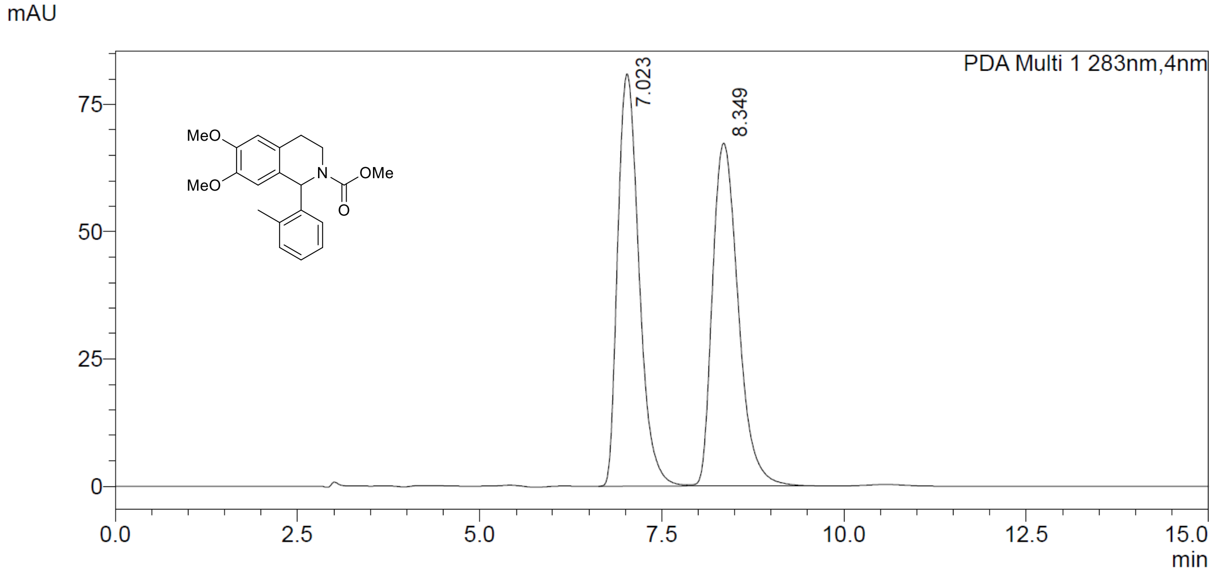

IA-3, *n*-heptane/i-PrOH 80:20, 298 K, 283 nm		
peak #	t_R / min	area / %
1	7.023	49.806
2	8.349	50.194

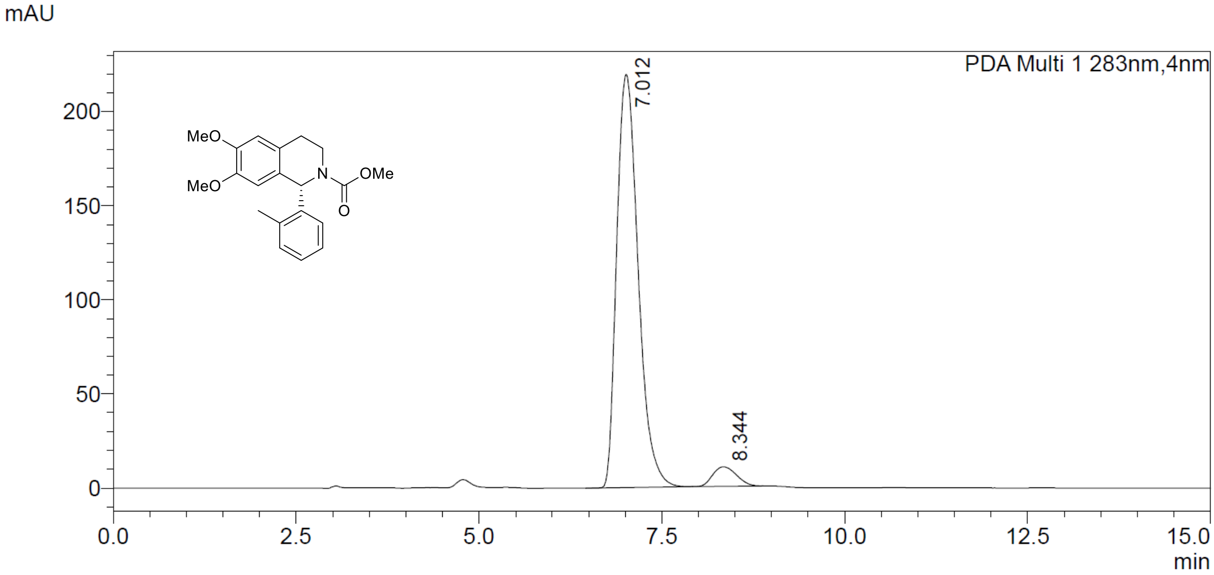

IA-3, *n*-heptane/i-PrOH 80:20, 298 K, 283 nm		
peak #	t_R / min	area / %
1	7.012	95.044
2	8.344	4.956

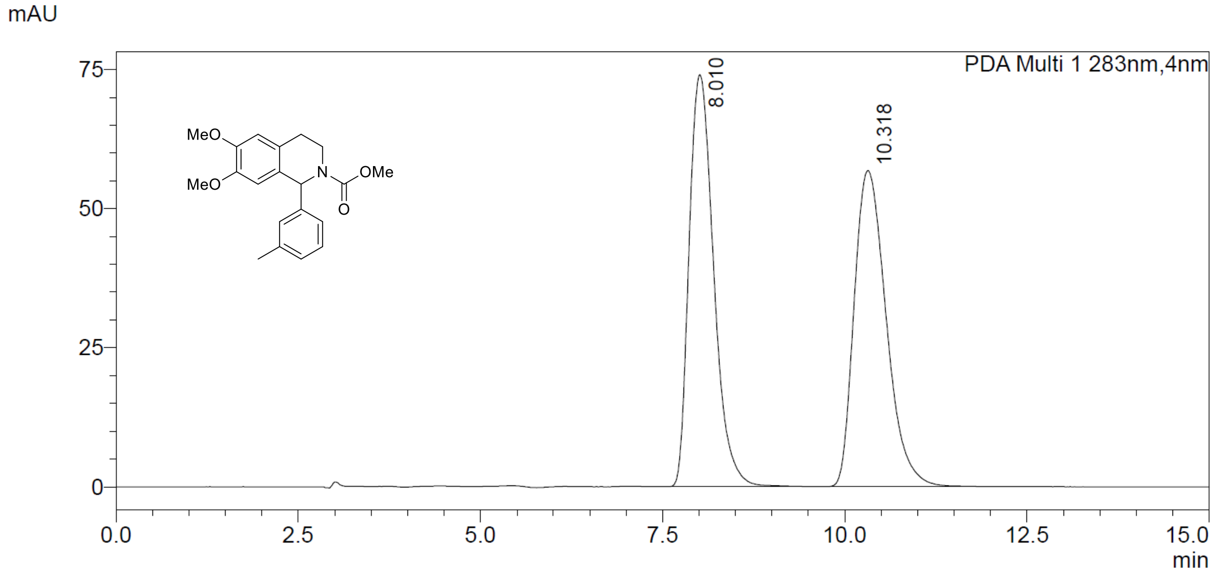

IA-3, *n*-heptane/i-PrOH 80:20, 298 K, 283 nm		
peak #	t_R / min	area / %
1	8.010	50.007
2	10.318	49.993

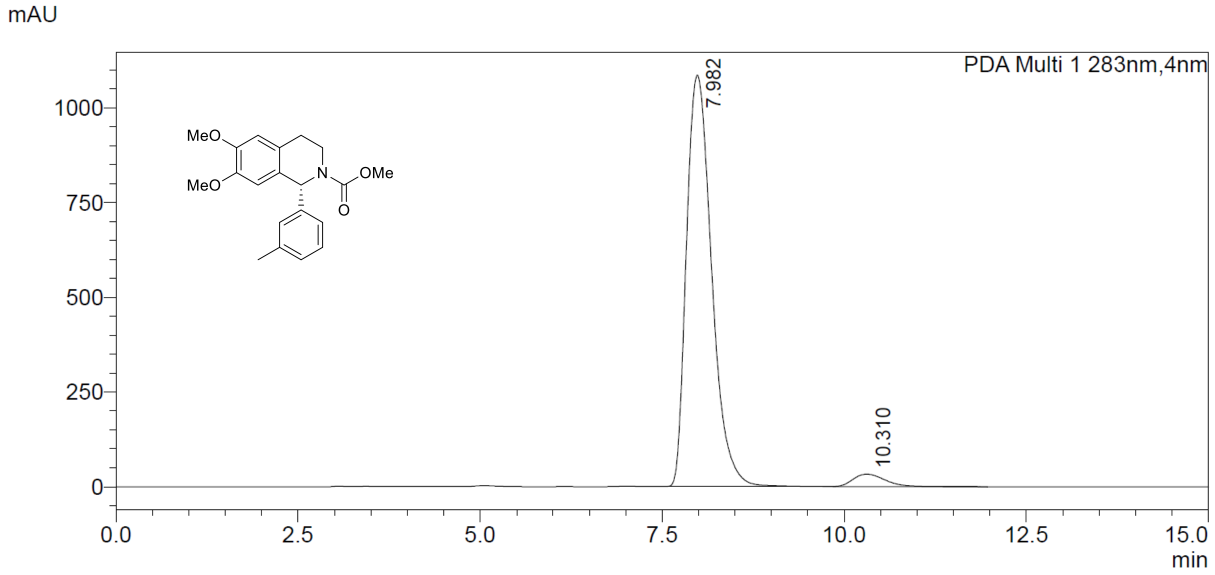

IA-3, *n*-heptane/i-PrOH 80:20, 298 K, 283 nm		
peak #	t_R / min	area / %
1	7.982	96.314
2	10.310	3.686

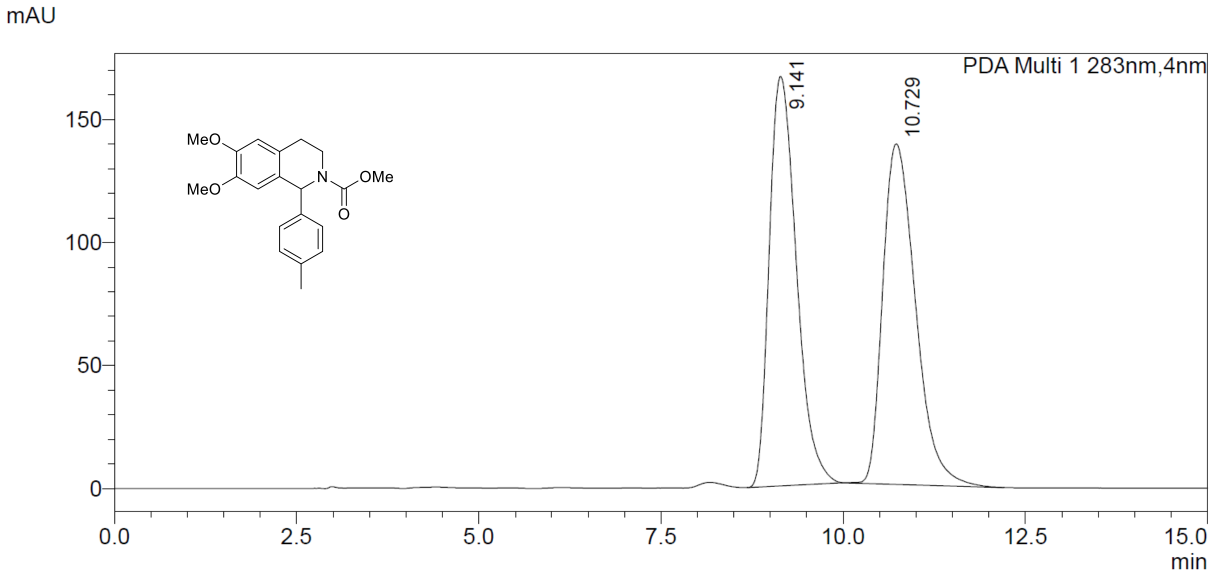

IA-3, *n*-heptane/i-PrOH 80:20, 298 K, 283 nm		
peak #	t_R / min	area / %
1	9.141	49.902
2	10.729	50.098

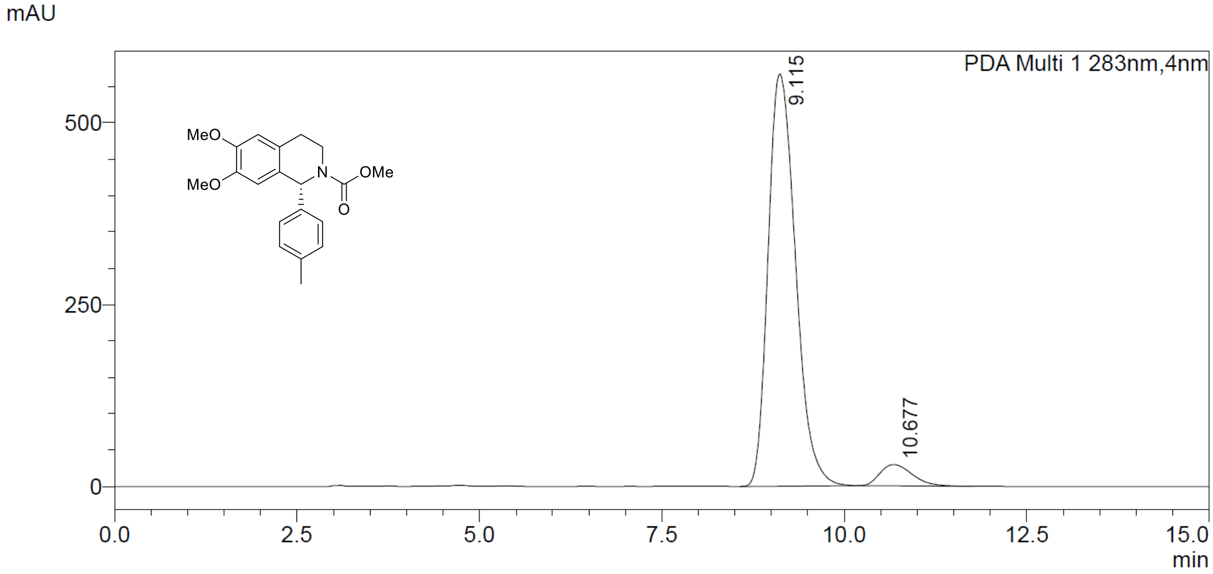

IA-3, *n*-heptane/i-PrOH 80:20, 298 K, 283 nm		
peak #	t_R / min	area / %
1	9.115	94.239
2	10.677	5.761

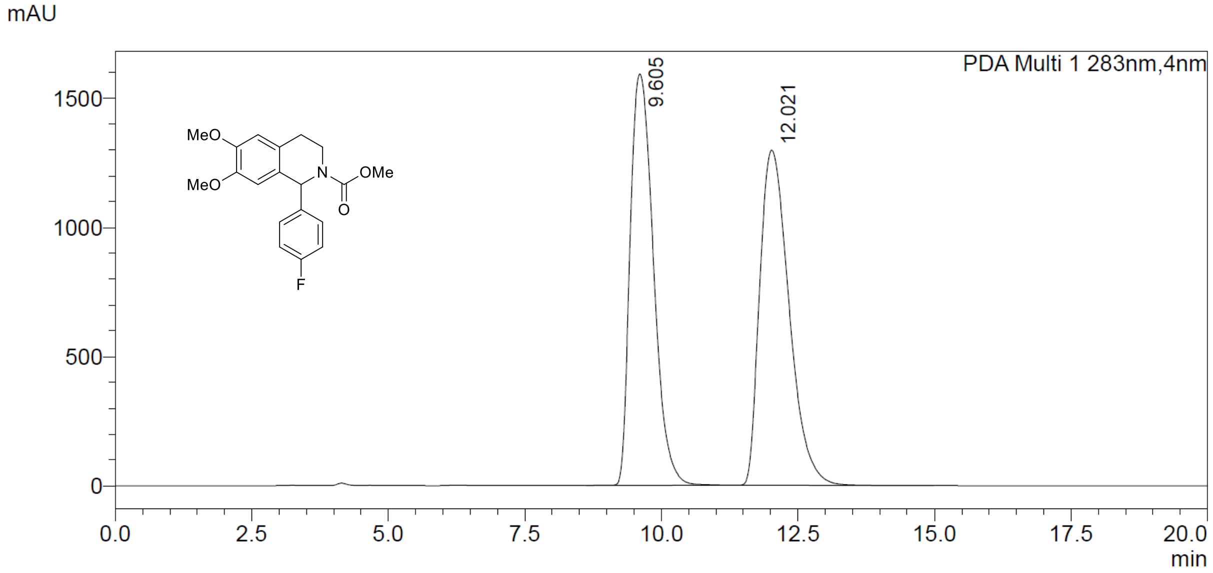

IA-3, *n*-heptane/i-PrOH 80:20, 298 K, 283 nm		
peak #	t_R / min	area / %
1	9.605	49.300
2	12.021	50.700

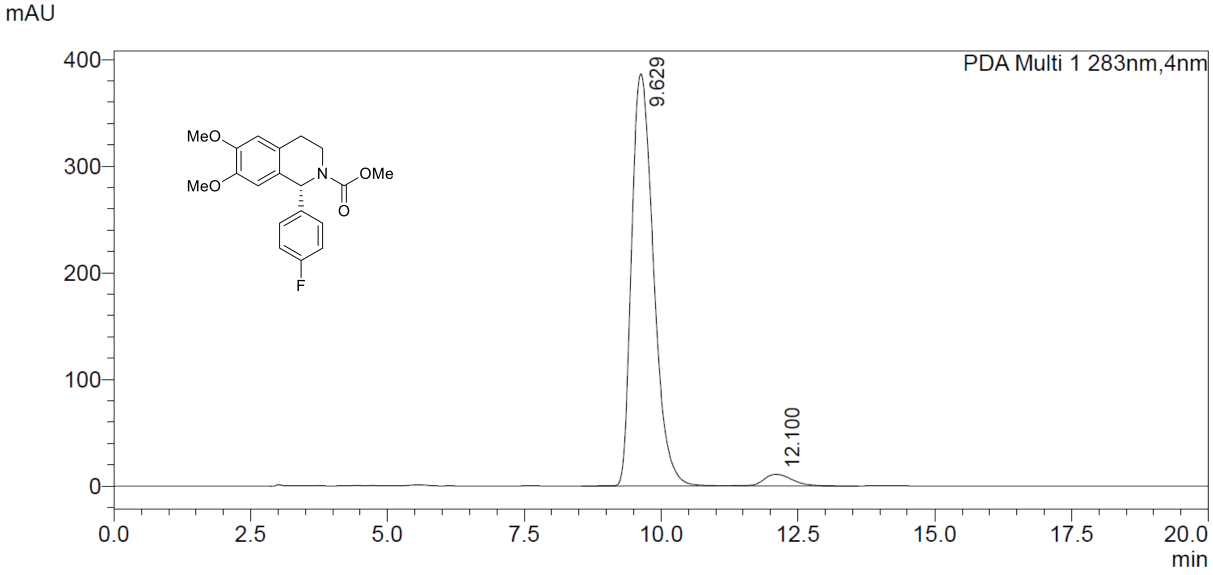

IA-3, *n*-heptane/i-PrOH 80:20, 298 K, 283 nm		
peak #	t_R / min	area / %
1	9.629	96.531
2	12.100	3.469

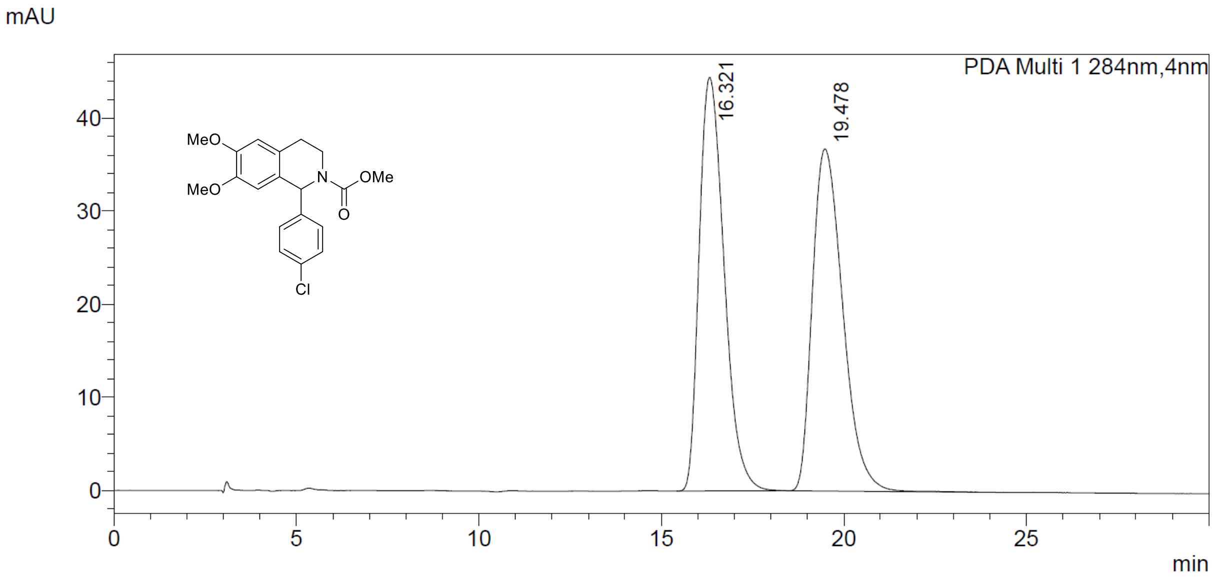

IA-3, *n*-heptane/i-PrOH 90:10, 298 K, 284 nm		
peak #	t_R / min	area / %
1	16.321	49.872
2	19.478	50.128

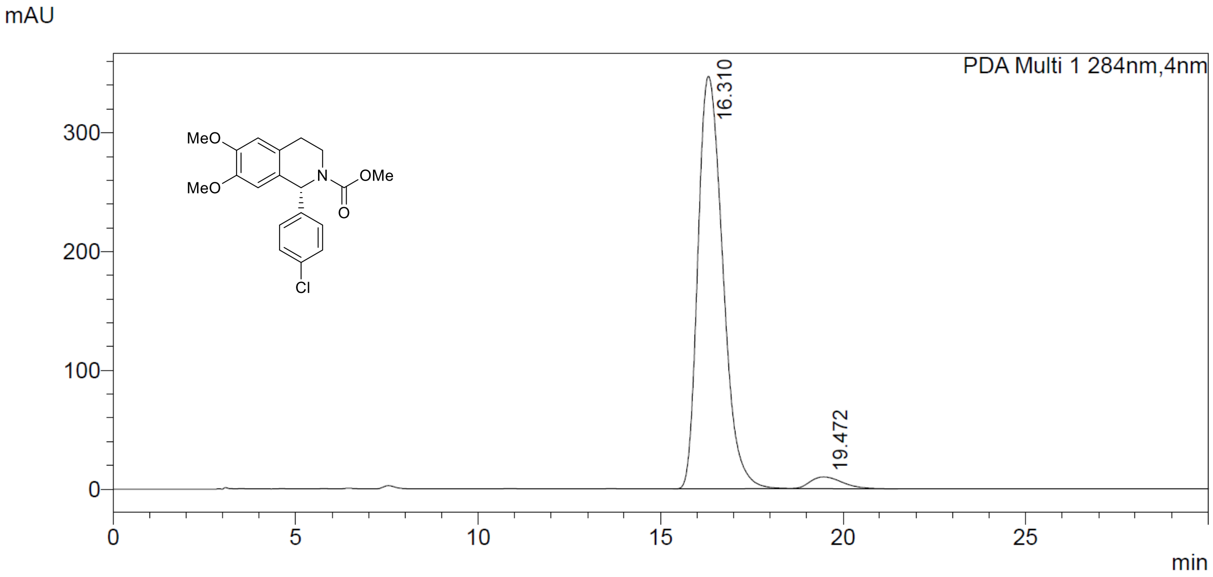

IA-3, *n*-heptane/i-PrOH 90:10, 298 K, 284 nm		
peak #	t_R / min	area / %
1	16.310	96.484
2	19.472	3.516

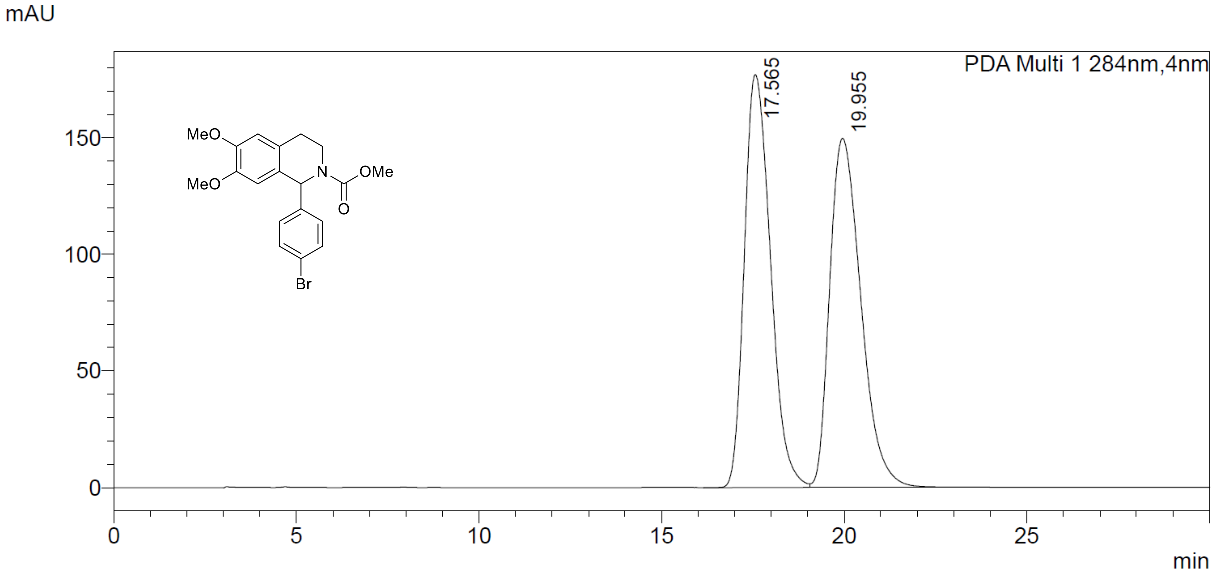

IA-3, *n*-heptane/i-PrOH 90:10, 298 K, 284 nm		
peak #	t_R / min	area / %
1	17.565	49.777
2	19.955	50.223

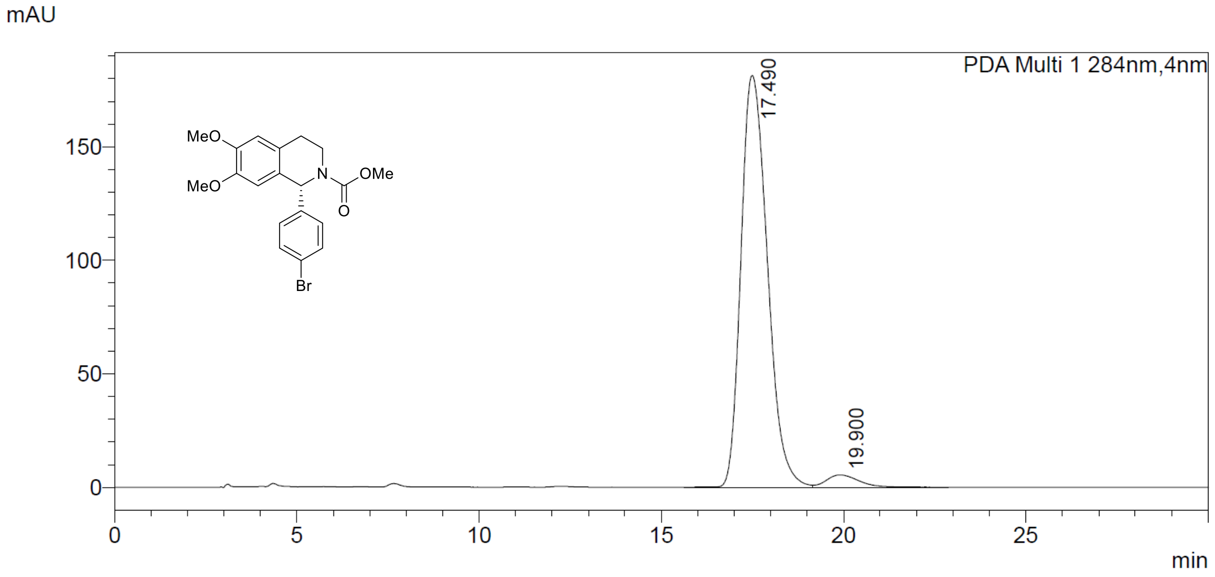

IA-3, *n*-heptane/i-PrOH 90:10, 298 K, 284 nm		
peak #	t_R / min	area / %
1	17.490	96.449
2	19.900	3.551

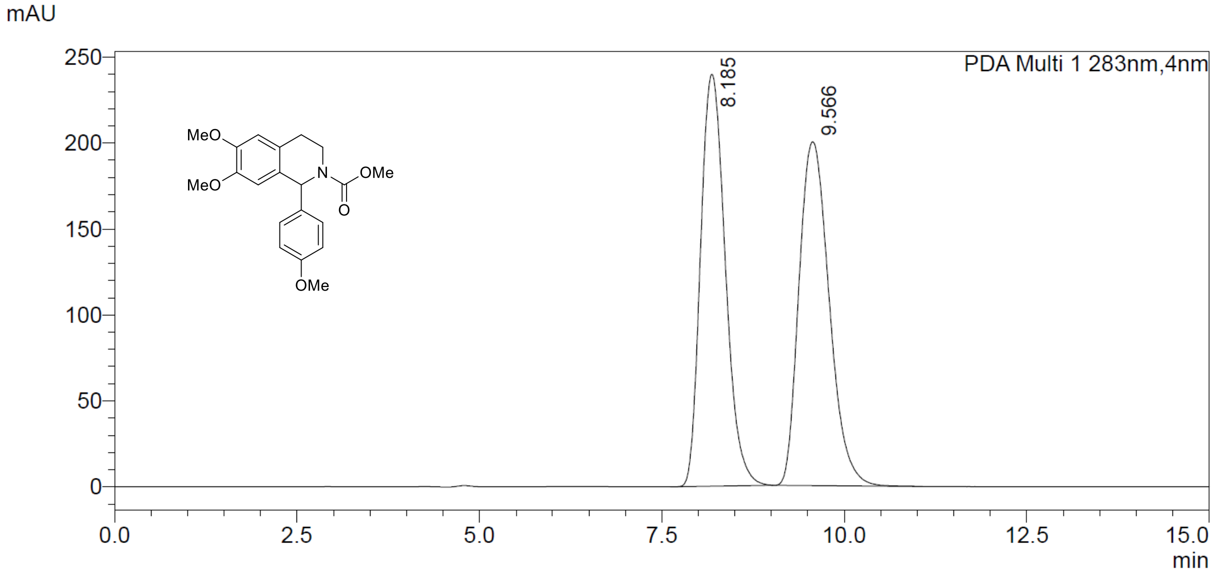

IA-3, *n*-heptane/i-PrOH 70:30, 298 K, 283 nm		
peak #	t_R / min	area / %
1	8.185	49.495
2	9.566	50.505

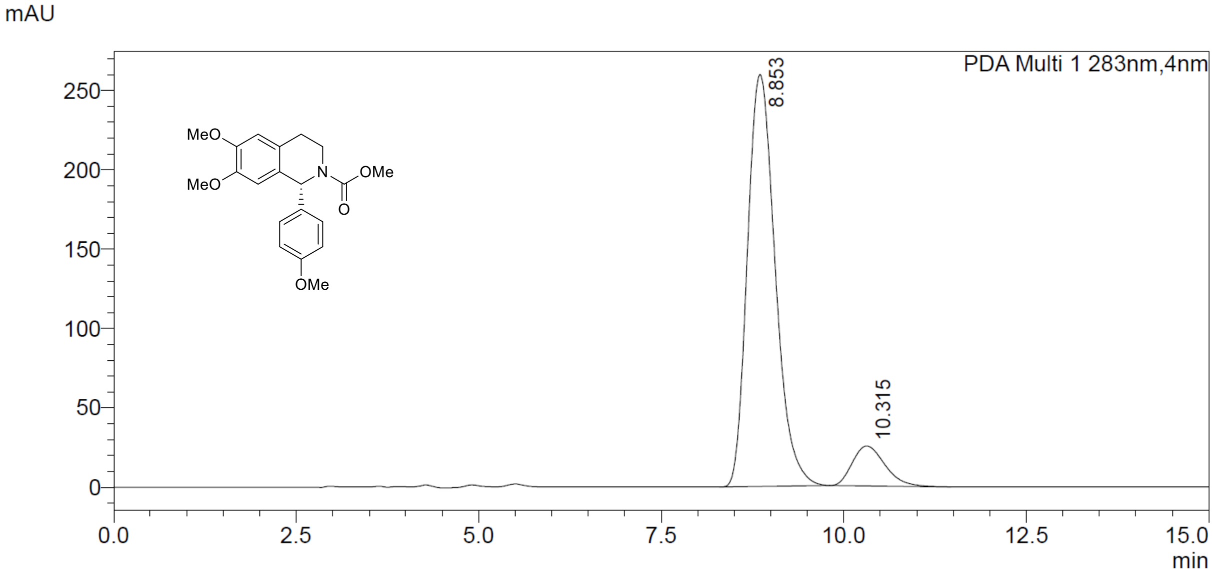

IA-3, *n*-heptane/i-PrOH 70:30, 298 K, 283 nm		
peak #	t_R / min	area / %
1	8.853	89.781
2	10.315	10.219

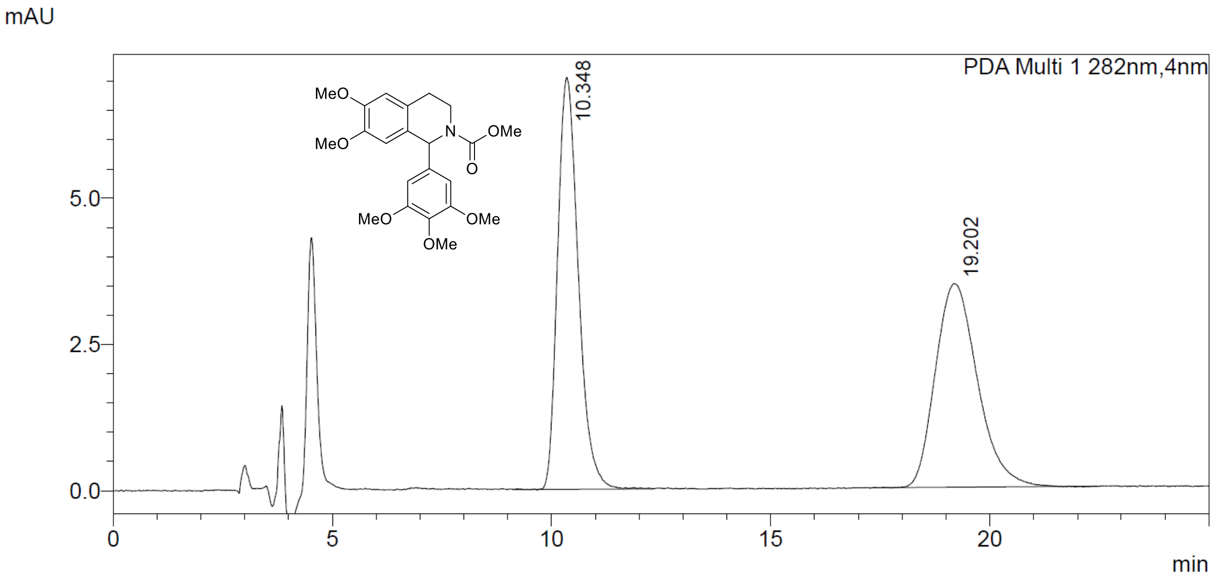

IA-3, *n*-heptane/i-PrOH 60:40, 298 K, 282 nm		
peak #	t_R / min	area / %
1	10.348	49.928
2	19.202	50.072

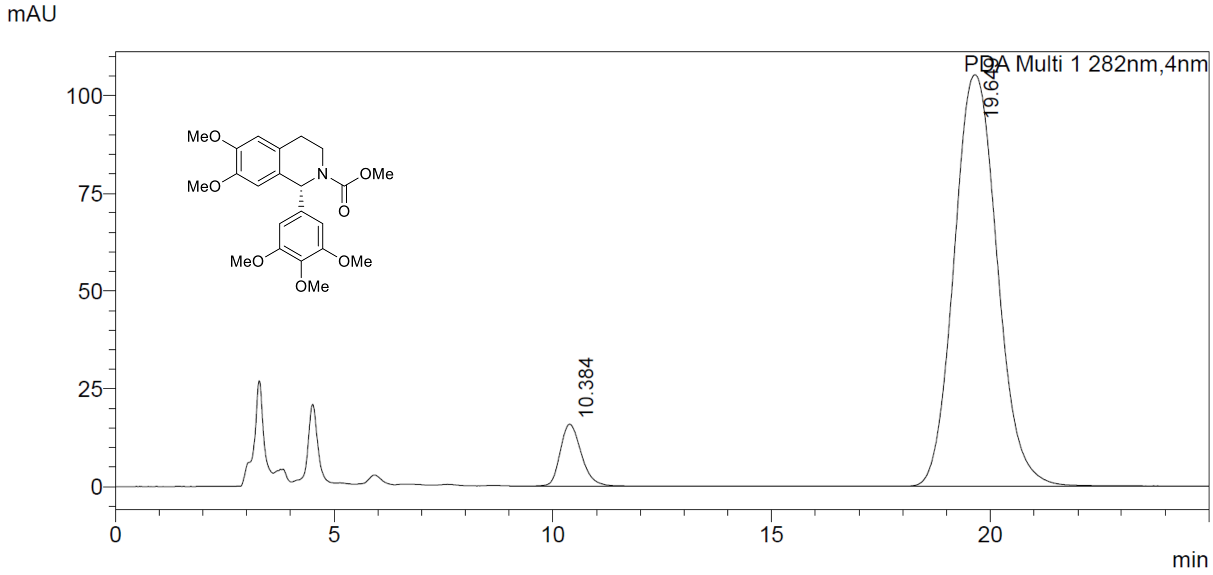

IA-3, *n*-heptane/i-PrOH 60:40, 298 K, 282 nm		
peak #	t_R / min	area / %
1	10.384	6.822
2	19.649	93.178

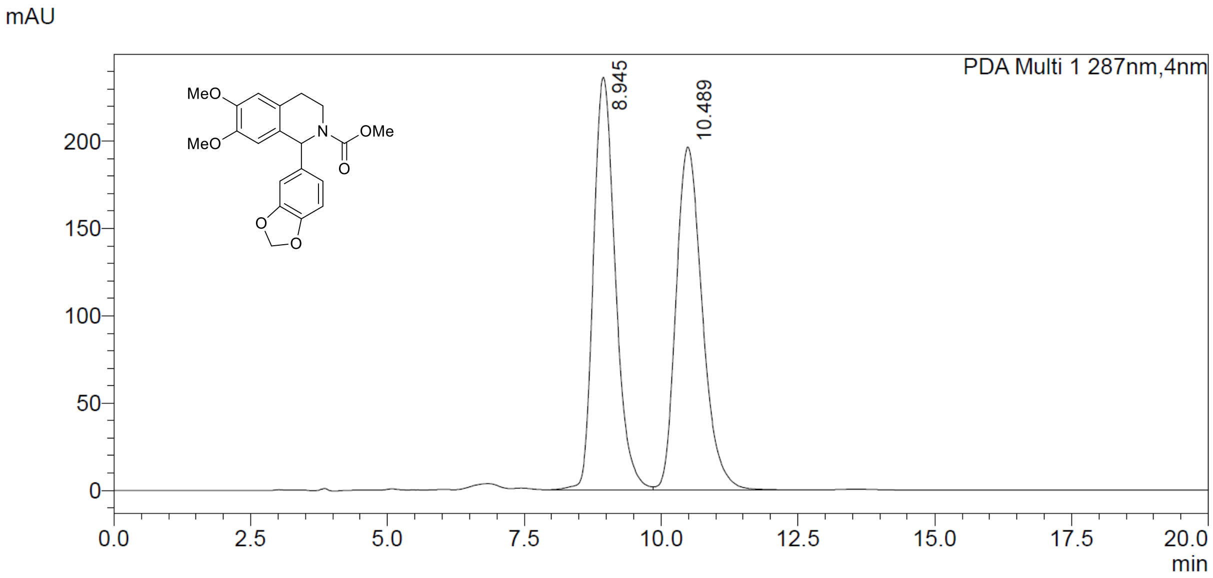

IA-3, *n*-heptane/i-PrOH 60:40, 298 K, 287 nm		
peak #	t_R / min	area / %
1	8.945	50.105
2	10.489	49.895

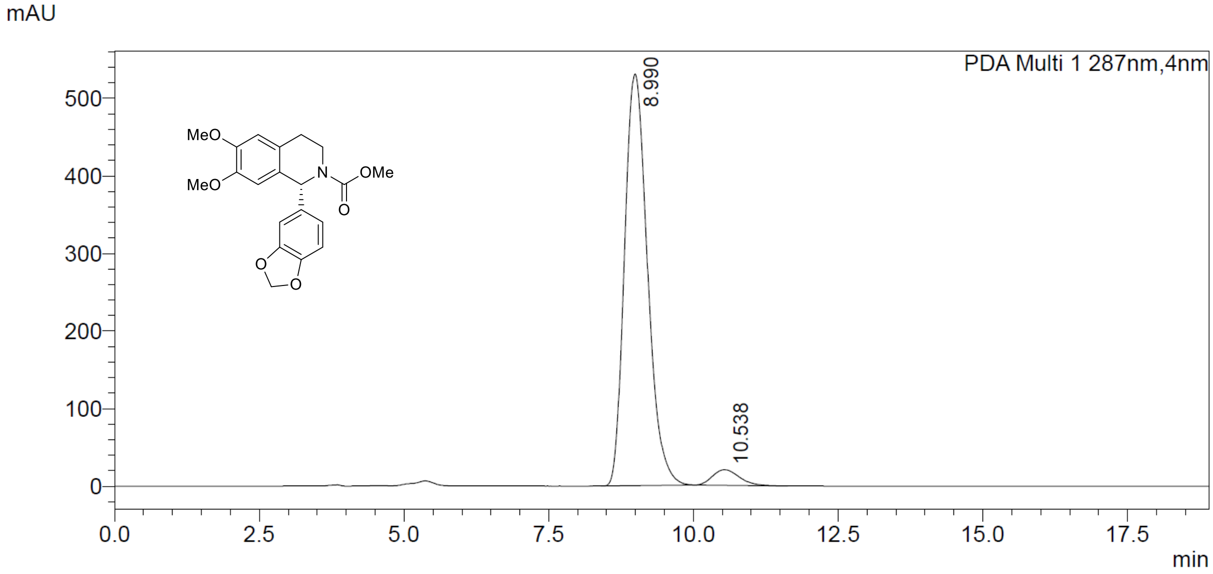

IA-3, *n*-heptane/i-PrOH 60:40, 298 K, 287 nm		
peak #	t_R / min	area / %
1	8.990	95.777
2	10.538	4.223

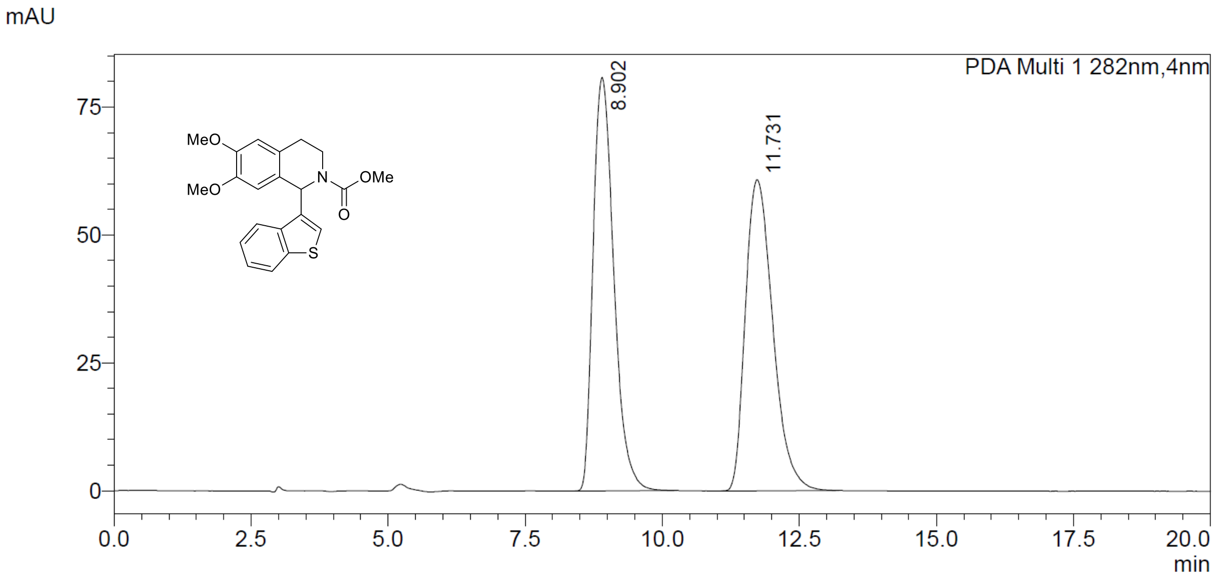

IA-3, *n*-heptane/i-PrOH 80:20, 298 K, 282 nm		
peak #	t_R / min	area / %
1	8.902	50.063
2	11.783	49.937

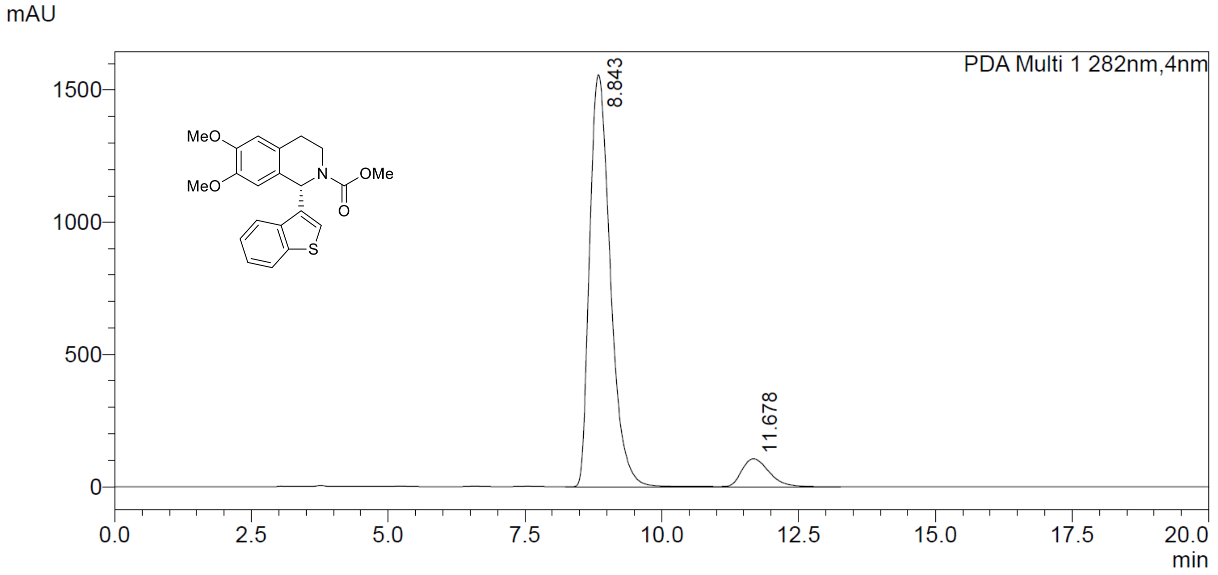

IA-3, *n*-heptane/i-PrOH 80:20, 298 K, 282 nm		
peak #	t_R / min	area / %
1	8.843	91.848
2	11.678	8.152

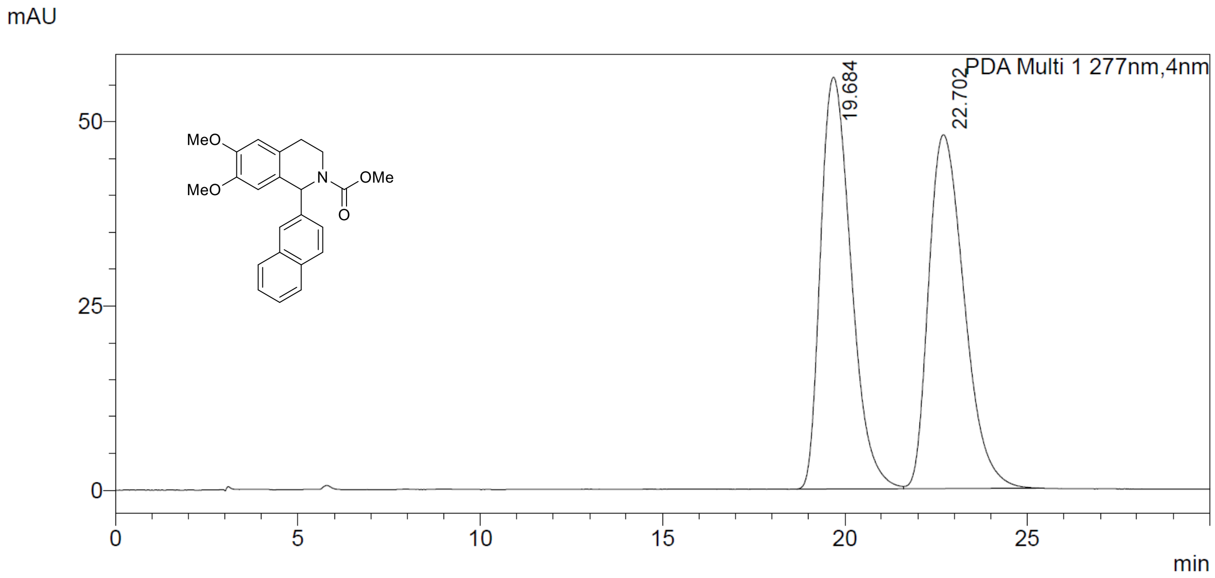

IA-3, *n*-heptane/i-PrOH 90:10, 298 K, 277 nm		
peak #	t_R / min	area / %
1	19.684	49.986
2	22.702	50.014

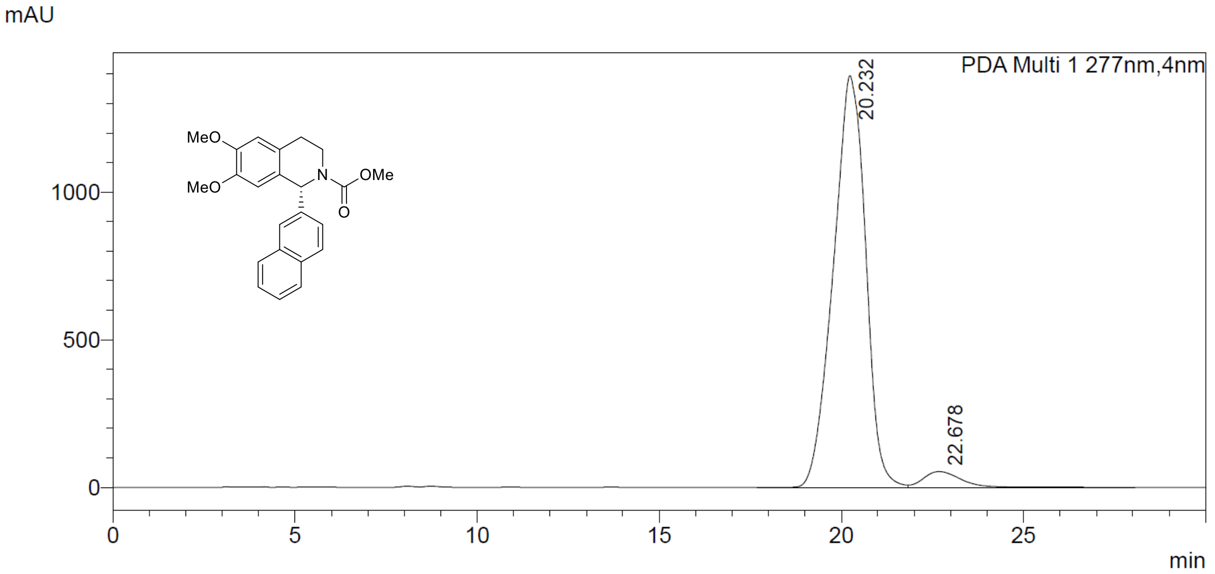

IA-3, *n*-heptane/i-PrOH 90:10, 298 K, 277 nm		
peak #	t_R / min	area / %
1	20.232	95.801
2	22.678	4.199

11. References

(1) Scharf, M. J.; List, B. A Catalytic Asymmetric Pictet–Spengler Platform as a Biomimetic Diversification Strategy toward Naturally Occurring Alkaloids. *J. Am. Chem. Soc.* **2022**, *144*, 15451–15456.

(2) Kütt, A.; Leito, I.; Kaljurand, I.; Sooväli, L.; Vlasov, V. M.; Yagupolskii, L. M.; Koppel, I. A. A Comprehensive Self-Consistent Spectrophotometric Acidity Scale of Neutral Brønsted Acids in Acetonitrile. *J. Org. Chem.* **2006**, *71*, 2829–2838.

(3) Kütt, A.; Tshepelevitsh, S.; Saame, J.; Lõkov, M.; Kaljurand, I.; Selberg, S.; Leito, I. Strengths of Acids in Acetonitrile. *Eur. J. Org. Chem.* **2021**, *2021*, 1407–1419.

(4) Kütt, A.; Rodima, T.; Saame, J.; Raamat, E.; Mäemets, V.; Kaljurand, I.; Koppel, I. A.; Garlyauskayte, R. Y.; Yagupolskii, Y. L.; Yagupolskii, L. M.; Bernhardt, E.; Willner, H.; Leito, I. Equilibrium Acidities of Superacids. *J. Org. Chem.* **2011**, *76*, 391–395.

(5) Kaupmees, K.; Tolstoluzhsky, N.; Raja, S.; Rueping, M.; Leito, I. On the Acidity and Reactivity of Highly Effective Chiral Brønsted Acid Catalysts: Establishment of an Acidity Scale. *Angew. Chem., Int. Ed.* **2013**, *52*, 11569–11572.

(6) Debrauwer, V.; Leito, I.; Lõkov, M.; Tshepelevitsh, S.; Parmentier, M.; Blanchard, N.; Bizet, V. Synthesis and Physicochemical Properties of 2-SF_5-(Aza)Indoles, a New Family of SF_5 Heterocycles. *ACS Org. Inorg. Au* **2021**, *1*, 43–50.

(7) Thiele, M.; Rose, T.; Lõkov, M.; Stadtfeld, S.; Tshepelevitsh, S.; Parman, E.; Opara, K.; Wölper, C.; Leito, I.; Grimme, S.; Niemeyer, J. Multifunctional Organocatalysts - Singly-Linked and Macrocyclic Bisphosphoric Acids for Asymmetric Phase-Transfer and Brønsted-Acid Catalysis. *Chem. Eur. J.* **2023**, *29*, e202202953.

(8) Mohebbati, N.; Sokolovs, I.; Woite, P.; Lõkov, M.; Parman, E.; Ugandi, M.; Leito, I.; Roemelt, M.; Suna, E.; Francke, R. Electrochemistry and Reactivity of Chelation-Stabilized Hypervalent Bromine(III) Compounds. *Chem. Eur. J.* **2022**, *28*, e202200974.

(9) Smallcombe, S. H.; Patt, S. L.; Keifer, P. A. WET Solvent Suppression and Its Applications to LC NMR and High-Resolution NMR Spectroscopy. *J. Magn. Reson. Ser. A* **1995**, *117*, 295–303.

(10) Maeda, S.; Harabuchi, Y.; Takagi, M.; Taketsugu, T.; Morokuma, K. Artificial Force Induced Reaction (AFIR) Method for Exploring Quantum Chemical Potential Energy Surfaces. *Chem. Rec.* **2016**, *16*, 2232–2248.

(11) Maeda, S.; Ohno, K.; Morokuma, K. Systematic Exploration of the Mechanism of Chemical Reactions: The Global Reaction Route Mapping (GRRM) Strategy Using the ADDF and AFIR Methods. *Phys. Chem. Chem. Phys.* **2013**, *15*, 3683–3701.

(12) Bannwarth, C.; Ehlert, S.; Grimme, S. GFN2-XTB - An Accurate and Broadly Parametrized Self-Consistent Tight-Binding Quantum Chemical Method with Multipole Electrostatics and Density-Dependent Dispersion Contributions. *J. Chem. Theory Comput.* **2019**, *15*, 1652–1671.

(13) Neese, F. The ORCA Program System. *Wiley Interdiscip. Rev. Comput. Mol. Sci.* **2012**, *2*, 73–78.

(14) Grimme, S.; Hansen, A.; Ehlert, S.; Mewes, J. M. R^2SCAN-3c: A “Swiss Army Knife” Composite Electronic-Structure Method. *J. Chem. Phys.* **2021**, *154*, 64103.

(15) Neese, F. Software Update: The ORCA Program System—Version 5.0. *Wiley Interdiscip. Rev. Comput. Mol. Sci.* **2022**, *12*, e1606.

(16) Harden, I.; Neese, F.; Bistoni, G. An Induced-Fit Model for Asymmetric Organocatalytic Reactions: A Case Study of the Activation of Olefins via Chiral Brønsted Acid Catalysts. *Chem. Sci.* **2022**, *13*, 8848–8859.

(17) Cossi, M.; Rega, N.; Scalmani, G.; Barone, V. Energies, Structures, and Electronic Properties of Molecules in Solution with the C-PCM Solvation Model. *J. Comput. Chem.* **2003**, *24*, 669–681.

(18) Mardirossian, N.; Head-Gordon, M. ωB97M-V: A Combinatorially Optimized, Range-Separated Hybrid, Meta-GGA Density Functional with VV10 Nonlocal Correlation. *J. Chem. Phys.* **2016**, *144*, 214110.

(19) Zheng, J.; Xu, X.; Truhlar, D. G. Minimally Augmented Karlsruhe Basis Sets. *Theor. Chem. Acc.* **2011**, *128*, 295–305.

(20) Weigend, F.; Ahlrichs, R. Balanced Basis Sets of Split Valence, Triple Zeta Valence and Quadruple Zeta Valence Quality for H to Rn: Design and Assessment of Accuracy. *Phys. Chem. Chem. Phys.* **2005**, *7*, 3297–3305.

(21) Lu, T.; Chen, Q. Independent Gradient Model Based on Hirshfeld Partition: A New Method for Visual Study of Interactions in Chemical Systems. *J. Comput. Chem.* **2022**, *43*, 539–555.

(22) Lu, T.; Chen, F. Multiwfn: A Multifunctional Wavefunction Analyzer. *J. Comput. Chem.* **2012**, *33*, 580–592.

(23) Humphrey, W.; Dalke, A.; Schulten, K. VMD: Visual Molecular Dynamics. *J. Mol. Graph.* **1996**, *14*, 33–38.

(24) Frisch, M. J.; Trucks, G. W.; Schlegel, H. B.; Scuseria, G. E.; Robb, M. A.; Cheeseman, J. R.; Scalmani, G.; Barone, V.; Petersson, G. A.; Nakatsuji, H.; Li, X.; Caricato, M.; Marenich, A. V.; Bloino, J.; Janesko, B. G.; Gomperts, R.; Mennucci, B.; Hratchian, H. P.; Ortiz, J. V.; Izmaylov, A. F.; Sonnenberg, J. L.; Williams-Young, D.; Ding, F.; Lipparini, F.; Egidi, F.; Goings, J.; Peng, B.; Petrone, A.; Henderson, T.; Ranasinghe, D.; Zakrzewski, V. G.; Gao, J.; Rega, N.; Zheng, G.; Liang, W.; Hada, M.; Ehara, M.; Toyota, K.; Fukuda, R.; Hasegawa, J.; Ishida, M.; Nakajima, T.; Honda, Y.; Kitao, O.; Nakai, H.; Vreven, T.; Throssell, K.; Montgomery, J. A. J.; Peralta, J. E.; Ogliaro, F.; Bearpark, M. J.; Heyd, J. J.; Brothers, E. N.; Kudin, K. N.; Staroverov, V. N.; Keith, T. A.; Kobayashi, R.; Normand, J.; Raghavachari, K.; Rendell, A. P.; Burant, J. C.; Iyengar, S. S.; Tomasi, J.; Cossi, M.; Millam, J. M.; Klene, M.; Adamo, C.; Cammi, R.; Ochterski, J. W.; Martin, R. L.; Morokuma, K.; Farkas, O.; Foresman, J. B.; Fox, D. J. Gaussian 16, Revision A.03. Gaussian, Inc., Wallingford CT 2016.

(25) Peng, Q.; Duarte, F.; Paton, R. S. Computing Organic Stereoselectivity-from Concepts to Quantitative Calculations and Predictions. *Chem. Soc. Rev.* **2016**, *45*, 6093–6107.

(26) Bickelhaupt, F. M.; Houk, K. N. Analyzing Reaction Rates with the Distortion/Interaction-Activation Strain Model. *Angew. Chem., Int. Ed.* **2017**, *56*, 10070–10086.
